# TransSurv: compatibility-aware transfer learning for target-cohort survival prediction

**DOI:** 10.1093/bib/bbag532

**Published:** 2026-10-27

**Authors:** Zheng Qu, Hongzhe Zhang, Katherine L Nathanson, Yi Fang, Hongzhe Li, Jing Wang

**Affiliations:** Department of Breast Surgical Oncology, National Cancer Center/National Clinical Research Center for Cancer/Cancer Hospital, Chinese Academy of Medical Sciences and Peking Union Medical College, 17 Panjiayuan Nanli, Chaoyang District, Beijing 100021, China; Department of Biostatistics, Epidemiology and Informatics, Perelman School of Medicine, University of Pennsylvania, 3600 Civic Center Boulevard, Philadelphia, PA 19104, United States; Department of Medicine, Perelman School of Medicine at the University of Pennsylvania, 421 Curie Boulevard, Philadelphia, PA 19104, United States; Department of Breast Surgical Oncology, National Cancer Center/National Clinical Research Center for Cancer/Cancer Hospital, Chinese Academy of Medical Sciences and Peking Union Medical College, 17 Panjiayuan Nanli, Chaoyang District, Beijing 100021, China; Department of Biostatistics, Epidemiology and Informatics, Perelman School of Medicine, University of Pennsylvania, 3600 Civic Center Boulevard, Philadelphia, PA 19104, United States; Department of Breast Surgical Oncology, National Cancer Center/National Clinical Research Center for Cancer/Cancer Hospital, Chinese Academy of Medical Sciences and Peking Union Medical College, 17 Panjiayuan Nanli, Chaoyang District, Beijing 100021, China

**Keywords:** transfer learning, survival analysis, Cox proportional hazards model, high-dimensional genomics, compatibility screening, cancer prognosis

## Abstract

Transcriptomic prognostic models are most useful for narrowly specified, event-limited target populations such as clinical-trial cohorts or stage-defined subtypes. Candidate auxiliary cohorts may carry related prognostic information but differ in disease context, assay platform, and population structure, so naive pooling—and transfer learning without compatibility screening—can erode target-cohort discrimination. We present TransSurv, a target-centered transfer-learning framework for high-dimensional Cox modeling that screens candidate auxiliary cohorts for empirical compatibility before borrowing, comes with non-asymptotic guarantees, and reports which auxiliary cohorts contributed. In the JAVELIN Renal 101 metastatic renal cell carcinoma trial—to our knowledge, the first transfer-learning survival evaluation with a randomized trial as the target—TransSurv raised held-out progression-free survival discrimination. It also achieved the highest mean discrimination in all five clinically defined target cohorts (kidney, ovarian, cervical, lung, breast) from The Cancer Genome Atlas (TCGA) and improved recurrence prediction in a cross-platform, cross-population Fudan University Shanghai Cancer Center triple-negative breast cancer analysis using TCGA and METABRIC auxiliaries.

## Introduction

Gene-expression prognostic models increasingly guide treatment and surveillance decisions in oncology. Their clinical utility is well established in early-stage hormone receptor–positive breast cancer, where prospective studies showed that genomic risk scores can spare many patients from chemotherapy while preserving benefit for higher-risk subgroups, findings that have since been incorporated into clinical guidance [1–4]. Yet the settings in which such models are most clinically useful are often narrowly defined, because outcome risk and treatment pathways are context-dependent. In these target populations, transcriptomic predictors are high-dimensional and strongly correlated, and modeling them stably within any single clinically specified target cohort is challenging—both because event counts may be limited and because predictors and outcomes are heterogeneous across patient populations.

One natural response to this data limitation is to augment the target cohort with external genomic studies. In principle, large public resources and prior disease-specific cohorts could stabilize estimation for clinically defined target populations. In practice, however, cohorts from these resources cannot simply be treated as additional samples from the target population for prognostic modeling. Across cancer types and stage groups, differences in molecular structure and outcome processes [5, 6] mean that even large pan-cancer repositories [7, 8] may not provide directly comparable observations for a narrowly defined target subgroup. Even within the same disease, differences in assay platform, preprocessing, case mix, and clinical management introduce systematic heterogeneity [9], and empirical studies have shown limited reproducibility of prognostic gene signatures across such shifts [10, 11]. Population structure creates a particularly consequential form of the same problem, as models developed predominantly in European-ancestry cohorts may not calibrate well in underrepresented groups [12–14]. These considerations suggest that the challenge is not simply to borrow more data, but to borrow selectively from related cohorts while protecting a pre-specified target population from harmful transfer, by which we mean transfer that yields worse target-cohort prediction than using the target data alone.

To address this problem, we present TransSurv, a transfer-learning framework for high-dimensional survival prediction in a target cohort that screens candidate auxiliary cohorts for compatibility before borrowing external gene-expression information. TransSurv is target-centered: baseline clinical effects are modeled separately within each cohort and are not borrowed across cohorts, whereas transfer is restricted to the gene-expression component. Rather than assuming that auxiliary cohorts match the target at every gene, the framework screens for empirical compatibility and borrows when shared prognostic structure outweighs cohort-specific differences. By design, the borrowing operation is also inspectable: TransSurv outputs a compatibility report together with cohort-resolved coefficient–deviation summaries that make explicit which auxiliary cohorts were retained and how much each contributed to the target-centered fit, providing the dataset-level transparency that has been emphasized in recent clinical-AI deployment guidance [15, 16].

TransSurv builds on a broader literature showing that borrowing across cohorts can improve survival prediction, although existing methods do so in different ways. Classical approaches combine cohort-specific fits or pool individual-level data across cohorts, with meta-analytic frameworks clarifying the tradeoff between efficiency and heterogeneity [17, 18]. More recent methods move beyond simple pooling by modeling what is shared across cohorts and what remains cohort-specific, a decomposition developed in high-dimensional integrative analyses that estimate shared and cohort-specific gene-expression effects jointly [19–22]. Transfer-learning approaches instead start from the target cohort and borrow from external sources while allowing target–auxiliary differences, a program developed for high-dimensional linear and generalized linear models [23, 24] that has recently reached time-to-event outcomes. Existing Cox-based transfer methods borrow from a designated source or from all supplied sources at once: Trans-Cox penalizes source–target discrepancies in coefficients and baseline hazards for a fixed registry source in a low-dimensional clinical model [25], CoxTL reweights designated source data against covariate and coefficient shift in moderate dimension [26], shared-support regularization pools all supplied cohorts under a common support [27], maximin aggregation transports site models robustly without target outcomes [28], and extensions reach interval-censored and cure settings [29]; meta-learners such as MetaSurv borrow shared representations across all cohorts [30]. What this literature does not address is prognostic modeling that faces two difficulties at once: transcriptome-scale dimension and many candidate auxiliary cohorts of unknown compatibility. The two difficulties compound each other. At this scale, compatibility cannot be assessed a priori and must instead be estimated from thousands of fitted coefficients. Heterogeneity, in turn, means that borrowing from the wrong cohorts can worsen target-cohort prediction. The central operational question is, therefore, which cohorts should be borrowed from at all. TransSurv treats compatibility as a quantity to be estimated and screens candidate auxiliary cohorts on fitted coefficient contrasts before any borrowing. The resulting target-centered fit carries non-asymptotic guarantees for both the post-screening estimator and the screening step, with rates that depend on the dimension only through *łog p* (Supplementary Theorems 1 and 2).

We hypothesized that, by selectively borrowing from empirically compatible auxiliary cohorts while excluding incompatible ones, TransSurv would improve target-cohort survival prediction more reliably than either target-only fitting or unscreened pooling. We tested this hypothesis in three complementary settings spanning a randomized clinical trial cohort, clinically defined target cohorts from The Cancer Genome Atlas (TCGA) across multiple disease contexts, and an independent cross-platform cohort. We began with the JAVELIN Renal 101 metastatic renal cell carcinoma trial cohort [31] as the target, using stage-stratified TCGA kidney renal clear cell carcinoma (KIRC) cohorts as candidate auxiliaries; to our knowledge, this is the first prospective randomized clinical trial cohort used as the target population for a transcriptomic transfer-learning survival method. We then assessed overall survival prediction across five clinically defined TCGA target cohorts spanning distinct disease and treatment contexts. Finally, we examined recurrence-free survival (RFS) prediction in a Fudan University Shanghai Cancer Center (FUSCC) triple-negative breast cancer cohort [32] using TNBC cohorts from TCGA and the Molecular Taxonomy of Breast Cancer International Consortium (METABRIC) as candidate auxiliaries, a cross-dataset setting with substantial population and measurement differences. In each application, we evaluated TransSurv against prespecified comparators using held-out discrimination metrics [C-index, time-dependent area under the receiver operating characteristic curve ( (AUC)], and we examined the fitted compatibility maps and coefficient–deviation summaries as a per-fit record of how auxiliary cohorts contributed to the target-centered fit. We complemented these analyses with new non-asymptotic guarantees and controlled-heterogeneity simulations that characterize the regimes in which compatibility-screened borrowing is expected to help. Open-source software for TransSurv, together with code to reproduce the analyses and simulations, is described in the Code availability statement.

## Results

### Overview of the TransSurv framework

Before turning to the empirical evaluations, we briefly summarize the TransSurv framework and the analysis workflow used throughout the study (Fig. 1). TransSurv is designed for clinically defined target cohorts when candidate auxiliary cohorts are available that may contain shared prognostic information. The central difficulty is that these candidate cohorts are heterogeneous: they may differ from the target in disease context, stage composition, assay platform, preprocessing pipeline, or population structure. TransSurv is therefore built around a selective-borrowing principle that evaluates auxiliary cohorts and borrows only when compatibility with the target is supported.

**Figure 1:**
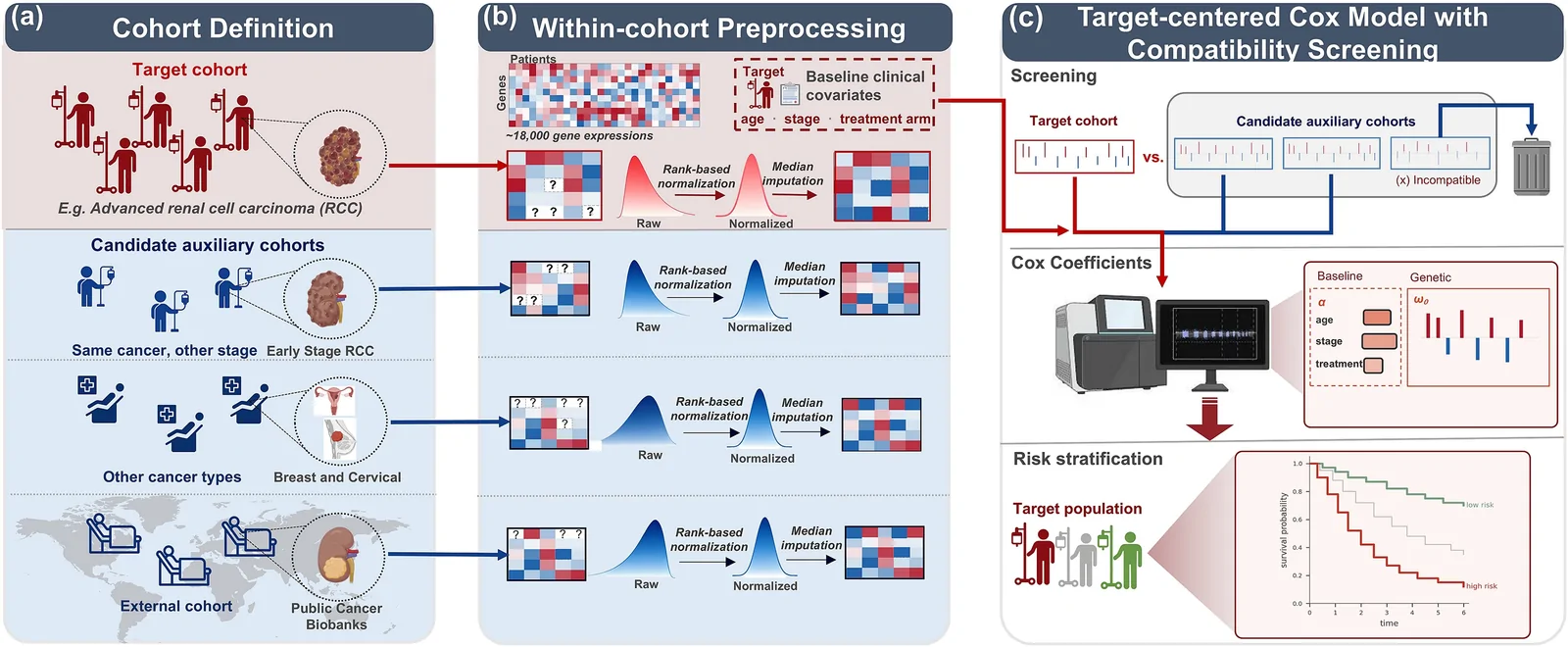
Overview of the TransSurv framework, in which candidate auxiliary cohorts are first defined relative to a clinically specified target cohort, then preprocessed separately within each cohort and aligned at the feature level, after which compatibility screening determines which auxiliary cohorts are retained for joint target-centered modeling. Created in BioRender. zgabf, J. (2026) https://BioRender.com/614cwyo.

The figure is best read from left to right. Panel (a) frames the study-design: a clinically specified target cohort is paired with multiple complementary candidate auxiliary cohorts that may offer prognostic information. In the applications considered here, these candidates may come from a related disease setting within the same cancer type, from other cancer types with potentially shared biology, or from independent external resources. This step defines a set of plausible borrowing candidates around a target cohort whose prediction performance is the primary object of interest.

Panel (b) emphasizes that borrowing is preceded by cohort-specific preprocessing. This step is essential because the cohorts entering TransSurv are not assumed to be measured on a common scale or to arise from a common population. Gene-expression features are therefore aligned across cohorts only after within-cohort normalization and imputation, so that downstream transfer captures shared prognostic structure despite differences in scale, missingness, or platform.

Panel (c) shows the central methodological step. Candidate auxiliary cohorts are screened for compatibility with the target, and only the retained cohorts enter the joint target-centered Cox fit. Within that fit, baseline clinical covariates remain target-specific, whereas borrowing is restricted to gene-expression effects, allowing shared prognostic structure to be reinforced while preserving cohort-specific correction where disagreement remains.

This workflow yields two outputs that are central to the results that follow: a fitted target-cohort survival model and an interpretable, compatibility-aware borrowing pattern across auxiliary cohorts. Formal model specification, tuning, implementation details, and statistical guarantees are provided in Section Materials and Methods and the Supplementary Information. We next evaluate the framework in three real-data settings spanning a clinical-trial target cohort, clinically defined TCGA target cohorts, and an independent cross-dataset TNBC cohort.

### TransSurv improves discrimination and within-arm risk stratification in a randomized renal cell carcinoma trial cohort

The lead application of this study is the most clinically stringent setting: prediction of progression-free survival (PFS) in the metastatic renal cell carcinoma cohort from the JAVELIN Renal 101 phase 3 trial [31]—a randomized cohort with treatment-standardized, protocol-defined endpoints and adjudicated follow-up—using stage-stratified (I–IV) TCGA KIRC cohorts as candidate auxiliary cohorts.

In held-out target patients, TransSurv showed higher discrimination than both target-only fitting and naive pooling across all reported metrics (Table 1). The mean C-index increased from *0.609* for both comparators to *0.652*, and the advantage persisted at both the 3-year and 6-year horizons. Naive pooling matched target-only at *0.609*, gaining nothing from the candidate auxiliary cohorts. MetaSurv also remained below TransSurv across all reported metrics in this setting. A baseline model using only the clinical covariates reached a C-index of *0.583 ± 0.030* (Supplementary Section 2.5). Within replications, TransSurv exceeded target-only fitting, naive pooling, and the clinical baseline in held-out C-index in 19 of 20 paired splits, MetaSurv in 18 of 20, and the $\ell _{2,1}$ comparator in 16 of 20.

**Table 1:** TransSurv prognostic modeling on the JAVELIN Renal 101 target cohort (PFS; 20 replications; mean *± * SD)

Target cohort	Metric	Target-only	Pooling	MetaSurv	$\ell _{2,1}$ transfer	TransSurv
JAVELIN Renal 101	C-index	*0.609 ± 0.034*	*0.609 ± 0.032*	*0.605± 0.041*	*0.641 ± 0.036*	$\mathbf{0.652 \pm 0.041}$
	AUC@3y	*0.655 ± 0.058*	*0.653 ± 0.059*	*0.630± 0.085*	*0.695 ± 0.061*	$\mathbf{0.698 \pm 0.066}$
	AUC@6y	*0.623 ± 0.046*	*0.622 ± 0.048*	*0.602± 0.061*	*0.672 ± 0.046*	$\mathbf{0.686 \pm 0.050}$

Bold indicates the best performance within each cohort-metric row, *† * indicates entries where a comparator exceeds TransSurv.

The gain in summary discrimination was also reflected in improved prognostic stratification within treatment arms. In a representative held-out split, the TransSurv risk score separated high- and low-risk patients more distinctly than the target-only model in both the sunitinib arm and the avelumab-plus-axitinib arm (Fig. 2).

**Figure 2:**
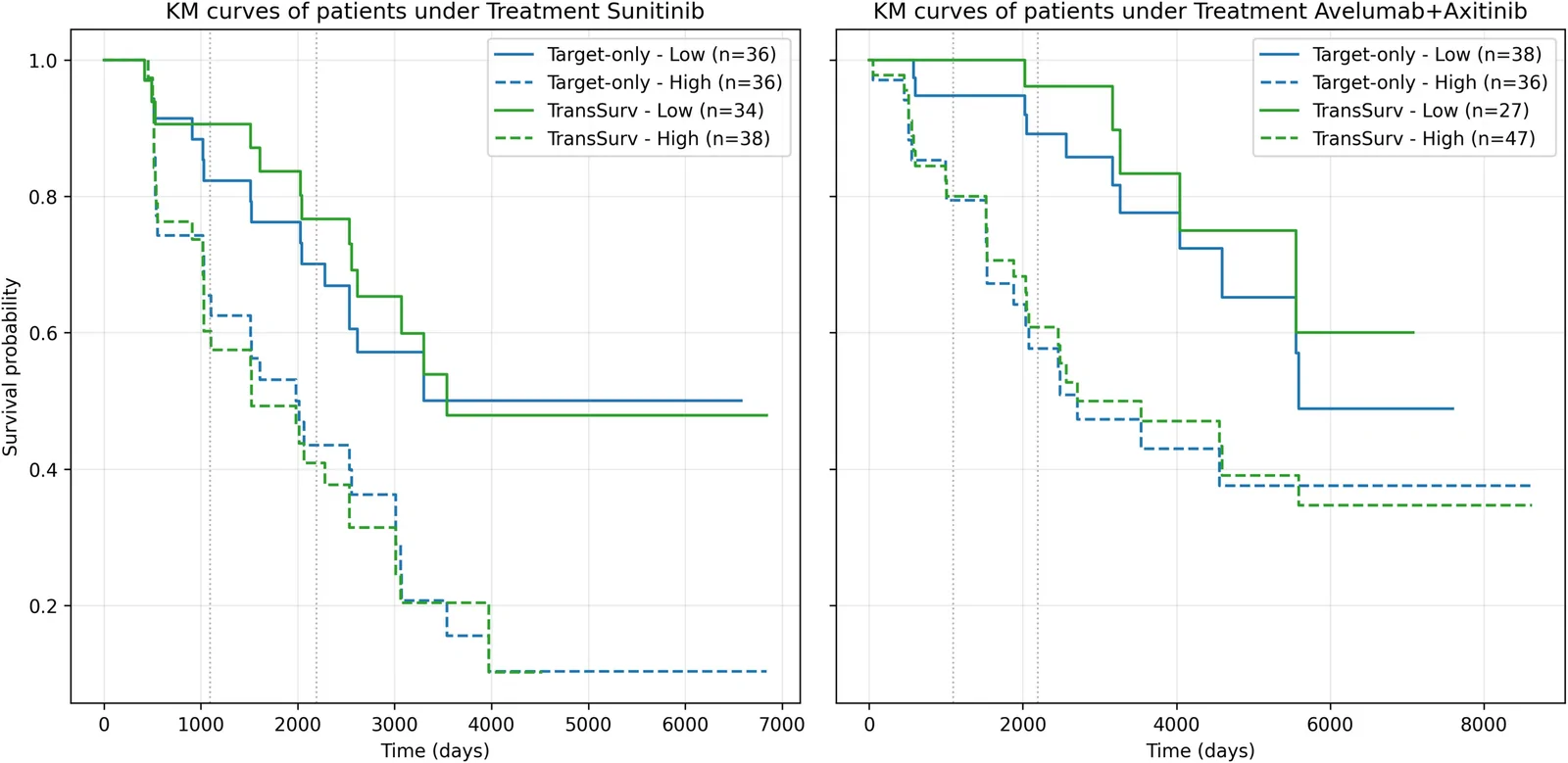
Treatment-arm-stratified Kaplan–Meier curves for low- and high-risk groups in a representative held-out split of the JAVELIN Renal 101 cohort, with target-only and TransSurv fits identified in each panel legend, solid and dashed lines denoting low- and high-risk groups, respectively, vertical dotted lines marking the prespecified 3-year and 6-year evaluation horizons, and low- and high-risk groups defined by *k*-means clustering with two clusters, applied to the predicted event probabilities at the prespecified horizons together with the model-derived risk score (Section Interpretability analyses).

Auxiliary-cohort selection was stable across resamples. Stage I KIRC was excluded in all 20 splits, whereas stages II–IV were retained. A representative coefficient-deviation heatmap showed stage II KIRC as the closest auxiliary cohort, with no visible deviations among the dominant target genes, whereas stages III and IV displayed only sparse deviations concentrated in less influential genes (Supplementary Fig. 1). Stage II was the closest auxiliary cohort in all 20 resamples. Together, these results indicate that TransSurv borrowed most strongly from the auxiliary cohort in closest empirical agreement with the target while allowing limited cohort-specific adjustments.

### TransSurv improves overall survival prediction across clinically defined TCGA target cohorts

We next asked whether the gain observed in the clinical-trial setting generalized across a broader set of clinically defined TCGA target populations. To anchor this breadth analysis in recognizable treatment contexts, we prespecified five TCGA target cohorts chosen to approximate relatively homogeneous disease settings across kidney, ovarian, cervical, lung, and breast cancers (abbreviated as KIRC, OV, CESC, LUAD, and BRCA). Their precise definitions are summarized in Supplementary Table 3. For each target, candidate auxiliary cohorts included the alternative stage-defined cohort within the same cancer type together with a broad panel of other TCGA cancer types, all subjected to the same compatibility-aware selection procedure.

Across the five clinically defined TCGA target cohorts, TransSurv achieved the highest mean C-index in every cohort (Table 2). The gain was most pronounced in KIRC and remained evident in BRCA, CESC, and LUAD, whereas improvement in OV was modest. The same pattern extended to time-dependent AUC at the cohort-specific evaluation horizons (Supplementary Table 1): TransSurv yielded the highest mean AUC in 8 of 10 target–horizon combinations, the exceptions being the earlier horizon in CESC, where target-only fitting was slightly better, and the later horizon in OV, where the $\ell _{2,1}$ comparator was. Taken together, these results show that compatibility-aware borrowing improves or preserves held-out discrimination across a diverse set of clinically defined TCGA populations rather than only in a single favorable disease setting. MetaSurv, which does not screen auxiliary cohorts for compatibility, fell below target-only in KIRC, LUAD, and OV. Within replications, TransSurv exceeded each comparator in Table 2 in held-out C-index in at least 18 of 20 paired splits in every target cohort; clinical-covariates-only baselines are reported in Supplementary Section 2.5.

**Table 2:** TransSurv prognostic modeling on TCGA (OS; 20 replications; mean *± * SD)

Cancer type	Metric	Target-only	MetaSurv	$\ell _{2,1}$ transfer	TransSurv
BRCA	C-index	*0.713± 0.079*	*0.751± 0.097*	*0.769± 0.091*	$\mathbf{0.774\pm 0.073}$
	AUC@$t_{1}$	*0.754± 0.100*	*0.765± 0.135*	*0.778± 0.079*	$\mathbf{0.820\pm 0.090}$
	AUC@$t_{2}$	*0.681± 0.127*	*0.738± 0.108*	*0.722± 0.093*	$\mathbf{0.767\pm 0.138}$
CESC	C-index	*0.682± 0.138*	*0.687± 0.124*	*0.677± 0.100*	$\mathbf{0.715\pm 0.102}$
	AUC@$t_{1}$	$\mathbf{0.698\pm 0.126}$ ^†^	*0.595± 0.165*	*0.614± 0.111*	*0.677± 0.155*
	AUC@$t_{2}$	*0.740± 0.187*	*0.549± 0.253*	*0.700± 0.125*	$\mathbf{0.741\pm 0.200}$
KIRC	C-index	*0.688± 0.053*	*0.638± 0.088*	*0.697± 0.068*	$\mathbf{0.736\pm 0.060}$
	AUC@$t_{1}$	*0.684± 0.067*	*0.640± 0.096*	*0.713± 0.082*	$\mathbf{0.725\pm 0.080}$
	AUC@$t_{2}$	*0.645± 0.062*	*0.650± 0.127*	*0.734± 0.075*	$\mathbf{0.744\pm 0.077}$
LUAD	C-index	*0.590± 0.072*	*0.509± 0.099*	*0.600± 0.052*	$\mathbf{0.615\pm 0.082}$
	AUC@$t_{1}$	*0.625± 0.087*	*0.532± 0.129*	*0.665± 0.074*	$\mathbf{0.676\pm 0.110}$
	AUC@$t_{2}$	*0.705± 0.142*	*0.535± 0.099*	*0.635± 0.058*	$\mathbf{0.711\pm 0.126}$
OV	C-index	*0.589± 0.060*	*0.554± 0.042*	*0.591± 0.061*	$\mathbf{0.592\pm 0.061}$
	AUC@$t_{1}$	*0.667± 0.100*	*0.594± 0.088*	*0.665± 0.049*	$\mathbf{0.670\pm 0.101}$
	AUC@$t_{2}$	*0.594± 0.114*	*0.559± 0.084*	$\mathbf{0.629\pm 0.084}$ ^†^	*0.598± 0.116*

AUC is computed at cohort-specific horizons $t_{1}$ and $t_{2}$ (Supplementary Table 1). Bold indicates the best performance within each cohort-metric row, $^\dagger $indicates comparator entries that exceed TransSurv.

The target-centered borrowing architecture visualized in Fig. 3 shows that knowledge borrowing was not diffuse or indiscriminate: across all five targets, the dominant target-associated signals were largely preserved, whereas auxiliary-cohort deviations remained localized to a smaller set of genes or program-level sectors. This pattern was strongest in KIRC and BRCA, where the target-centered structure remained visually coherent after transfer, and weakest in OV, where the target signal was more fragmented and the performance gain was correspondingly modest. The main implication is that TransSurv is not merely selecting cohorts that improve prediction; it is recovering a target-centered prognostic architecture in which compatible auxiliary cohorts reinforce dominant target signals while leaving residual disagreement confined to limited auxiliary-specific corrections. Detailed per-target reference versions of the coefficient–deviation heatmaps are provided in Supplementary Section 2.4.2.

**Figure 3:**
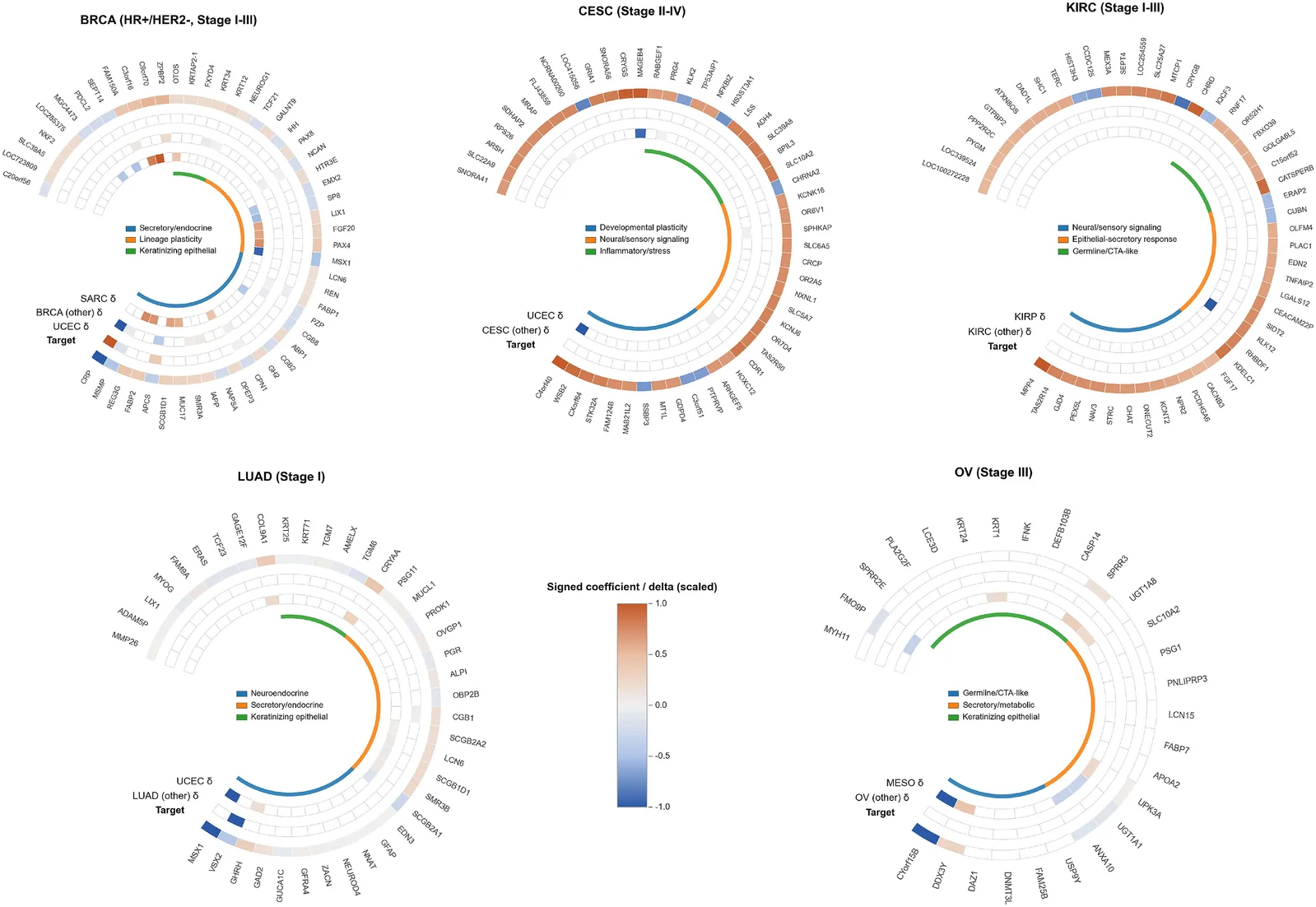
Target-centered borrowing architectures across the five clinically defined TCGA target cohorts, with each panel summarizing the target coefficient pattern together with retained auxiliary-cohort deviations in a representative fit and highlighting where dominant target-associated signals are preserved and where cohort-specific correction remains localized.

In every TCGA target, the alternative stage-defined cohort within the same cancer type was retained, providing a consistent within-cancer compatibility anchor before broader cross-cancer borrowing was considered. Beyond that anchor, Fig. 4 shows that between-cancer compatibility was sparse, asymmetric, and strongly target-dependent. A small number of cross-cancer cohorts emerged repeatedly as compatible for each target—most prominently KIRP for KIRC and UCEC for CESC—whereas OV displayed a broader but weaker compatibility pattern, in keeping with its more modest gain in held-out discrimination. The target-specific reference profiles in Supplementary Section 3 place these retained cohorts against the full background of screened-out cohorts and show the across-replication variability of the compatibility estimates, making clear that the main-text landscapes are condensed summaries of a broader compatibility ordering rather than selected examples. Read together with the borrowing architectures in Fig. 3, these compatibility landscapes show that the cohorts most often supporting transfer were specific disease contexts that repeatedly aligned with each target’s prognostic structure.

**Figure 4:**
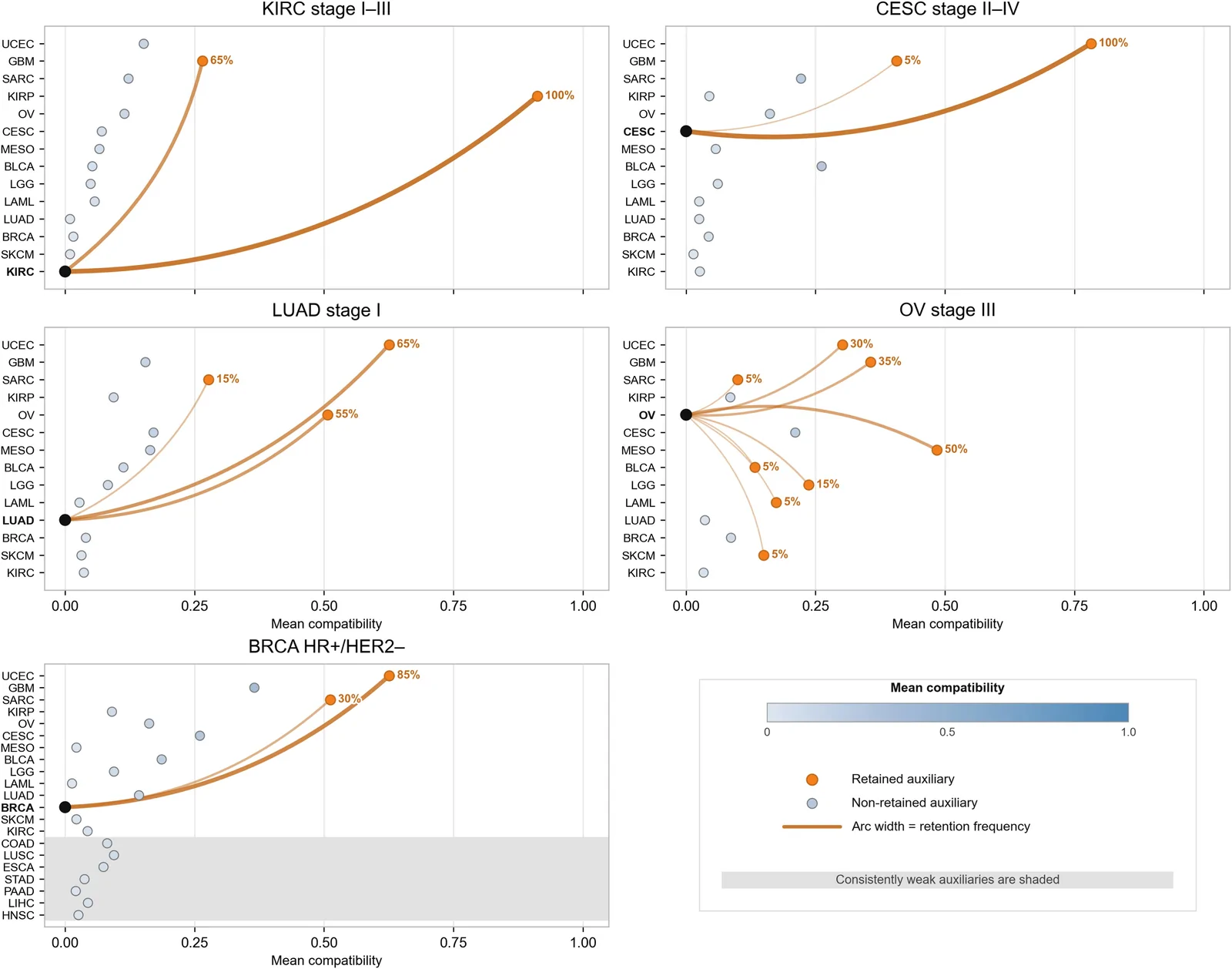
Compatibility landscapes across the five clinically defined TCGA target cohorts, in which points summarize the replication-averaged compatibility of candidate auxiliary cohorts with each target, retained auxiliaries are highlighted, and arc width indicates retention frequency across replications.

### TransSurv transfers across independent TNBC datasets

We finally evaluated whether TransSurv remained beneficial in a clinically matched cross-dataset TNBC setting, using the FUSCC TNBC cohort as the target and TNBC cohorts from TCGA and METABRIC as candidate auxiliaries. This was the most measurement-heterogeneous application in the paper, and therefore the one with the largest covariate-distribution shift: FUSCC and TCGA contributed sequencing-based expression profiles, whereas METABRIC contributed microarray-derived expression. To accommodate this asymmetry, we used auxiliary-specific deviation penalties with the TCGA contrast penalized fourfold more strongly than the METABRIC contrast, allowing larger cohort-specific correction for the more distinct METABRIC auxiliary. TransSurv again achieved the highest mean C-index for predicting RFS, increasing discrimination from 0.700 for target-only fitting and 0.703 for naive pooling to 0.727, while MetaSurv reached 0.657 and the $\ell _{2,1}$-regularized transfer comparator 0.692 (Table 3). Performance at the early 0.5-year horizon was similar across methods, with a small advantage for naive pooling, but TransSurv provided the highest discrimination at 1.5 years (0.778 versus 0.725 for target-only). Within replications, TransSurv exceeded each comparator in held-out C-index in at least 18 of 20 paired splits, and a clinical-covariates-only baseline (age and stage) reached a C-index of *0.673 ± 0.064* (Supplementary Section 2.5).

**Table 3:** TransSurv prognostic modeling on the FUSCC TNBC target cohort (RFS; 20 replications; mean *± * SD)

Target cohort	Metric	Target-only	Pooling	MetaSurv	$\ell _{2,1}$ transfer	TransSurv
FUSCC TNBC	C-index	*0.700± 0.056*	*0.703 ± 0.079*	*0.657 ± 0.039*	*0.692 ± 0.054*	$\mathbf{0.727\pm 0.047}$
	AUC@0.5y	*0.788± 0.147* ^†^	$\mathbf{0.812 \pm 0.165}$ ^†^	*0.754 ± 0.095*	*0.709 ± 0.060*	*0.778± 0.150*
	AUC@1.5y	*0.725± 0.091*	*0.714 ± 0.138*	*0.676 ± 0.062*	*0.690 ± 0.109*	$\mathbf{0.778\pm 0.093}$

Bold indicates the best performance within each cohort-metric row, $^\dagger $indicates entries where a comparator exceeds TransSurv.

Both TCGA TNBC and METABRIC TNBC were retained in all 20 resamples. The corresponding coefficient–deviation heatmap in Supplementary Section 2.4.3 showed a clear asymmetry between the two auxiliaries: the TCGA deviation remained highly sparse and near zero across most dominant target genes, whereas the METABRIC deviation was broader and less sparse. This pattern is consistent with the stronger measurement mismatch of METABRIC and with the lighter penalty placed on its deviation term, indicating that TransSurv preserved shared TNBC prognostic signal while allowing substantially larger cohort-specific correction where cross-platform disagreement remained.

The FUSCC application also exercises TransSurv in a setting where the target cohort is of distinct geographic and ancestral origin from both auxiliary cohorts, indicating that the framework remains applicable when the target population is under-represented in TCGA-scale resources.

## Discussion

In the JAVELIN Renal 101 randomized trial cohort, TransSurv improved held-out PFS discrimination over both target-only fitting and unscreened pooling. To our knowledge, this is the first prospective randomized clinical trial cohort in which a transcriptomic transfer-learning survival method has been evaluated. The same compatibility-aware borrowing strategy improved held-out target-cohort discrimination in five clinically defined TCGA target cohorts spanning kidney, ovarian, cervical, lung, and breast cancers (Section TransSurv improves overall survival prediction across clinically defined TCGA target cohorts) and in an independent cross-platform FUSCC TNBC cohort using TCGA and METABRIC auxiliaries (Section TransSurv transfers across independent TNBC datasets). Together, these findings support the central premise of the framework: auxiliary cohorts need not be fully exchangeable with the target to be useful, but compatibility must be learned rather than assumed.

Across these applications, the empirical gains were heterogeneous rather than uniform. The clearest applied advantage appeared in the JAVELIN Renal 101 trial cohort, where TransSurv improved discrimination over both target-only fitting and unscreened pooling and yielded clearer within-arm prognostic separation. Across the five TCGA targets, TransSurv achieved the highest mean C-index in every cohort (Table 2), with the magnitude of improvement varying by target. In FUSCC TNBC, TransSurv improved overall discrimination and the longer-horizon AUC, while short-term metrics were similar across methods. The across-replication variability of the held-out estimates remained non-negligible in several settings, which is expected in clinically narrow, event-limited target cohorts and should temper interpretation of small numerical differences between methods.

The compatibility summaries and coefficient–deviation heatmaps suggest that borrowing was highly structured. In the JAVELIN Renal 101 analysis, stage I KIRC was consistently excluded, whereas stages II–IV were consistently retained, with stage II showing the strongest compatibility with the target across resamples. Across the TCGA targets, borrowing was again selective: the alternative stage-defined cohort within the same cancer type was stably retained, and cross-cancer borrowing was sparser and target-specific. For example, KIRP was consistently retained for the KIRC target, supporting the view that closely related renal subtypes can share substantial prognostic structure. The recurrent appearance of UCEC among the higher-compatibility auxiliary cohorts is also biologically plausible, as pan-gynecologic studies have shown that endometrial tumors span multiple molecular clusters and can align with ovarian serous-like disease rather than occupying a single isolated niche. By contrast, the OV IIIC target showed the most dispersed and unstable compatibility profile, consistent with its comparatively modest performance gain. The apparent compatibility between OV and LUAD should therefore be interpreted cautiously, as it is more likely to reflect recurring stromal, immune, or EMT-associated programs than genuine prognostic compatibility. In the FUSCC TNBC analysis, the fitted heatmap showed a clear auxiliary asymmetry: the TCGA deviation remained comparatively sparse, whereas the METABRIC deviation was broader and less sparse. This is consistent with the stronger cross-platform mismatch of METABRIC and with the asymmetric penalty calibration used in that application, which allowed larger cohort-specific correction for METABRIC while preserving shared TNBC signal where alignment remained.

Beyond their descriptive value, the compatibility maps and coefficient–deviation heatmaps produced by TransSurv constitute an inspectable borrowing-disclosure artifact: a per-fit, dataset-level record of which auxiliary cohorts were borrowed from, by how much, and on which genes the borrowing operated. This record is produced by construction whenever a TransSurv risk score is reported, providing the kind of dataset-level provenance that recent clinical-AI deployment guidance emphasizes [15, 16]. Within the record, the repeated retention of a small subset of auxiliary cohorts argues against indiscriminate borrowing, which can lead to harmful transfer, while the cohort-specific contrasts visible even among retained cohorts argue against naive pooling. The broad concordance between the fitted borrowing pattern and established disease relationships further suggests that the retained cohorts are biologically plausible matches, not merely statistical conveniences. The components of the compatibility report are mapped to specific Good Machine Learning Practice principles in Supplementary Section 8.

Two non-asymptotic results characterize when TransSurv is expected to help. The oracle-rate theorem (Supplementary Theorem 1) shows that compatible auxiliary cohorts reduce target-cohort estimation error at the high-dimensional rate $s\log p/(n_{0}+n_{\mathcal{A}})$, up to a residual-mismatch term governed by an explicit covariate-shift factor $C_\Sigma (\mathcal{A})$ that measures the mismatch between auxiliary and target information matrices, so the gain from borrowing is largest when auxiliary cohorts are abundant and their underlying structure is close to the target’s. Covariate shift across cohorts is thus not assumed away but enters the guarantee as a quantified cost. The screening-consistency theorem (Supplementary Theorem 2) shows that the fitted-contrast diagnostic recovers a nonempty oracle transferable subset under stated separation and event-information conditions, so the screening step reliably identifies the compatible cohorts when at least some are genuinely compatible. Controlled-heterogeneity simulations (Supplementary Section 5) corroborate both regimes, including under covariate shift: the borrowing gain attenuates but persists as design heterogeneity increases, and in a pure-shift arm at *p = 20 000*, in which cohorts differ only in their covariate distributions, borrowing remains beneficial (Supplementary Section 5.3).

In all analyses, our aim was to evaluate whether the TransSurv pipeline improves target-cohort prediction under a common and fair analysis protocol. The emphasis was therefore on the added value of compatibility screening and cohort-specific deviations within a target-centered borrowing framework, rather than on extracting the last possible increment of predictive accuracy from each application. The TCGA analyses likely represent a more favorable transfer environment than many routine cross-institution applications, because predictor measurement, preprocessing, and endpoint definition are comparatively aligned; they should therefore be interpreted primarily as proof of concept for borrowing across clinically defined targets and cancer types. The compatibility plots and coefficient–deviation heatmaps are descriptive summaries of fitted prognostic structure under the current target-centered Cox specification and feature representation; they help explain why TransSurv borrows from some cohorts and not others, but they do not, by themselves, establish shared biological mechanism. Calibration was not a primary endpoint of this study, because absolute calibration depends not only on the estimated gene-expression effects but also on the target baseline hazard and on treatment, follow-up, and censoring processes that differed across the datasets and were not fully harmonized.

Several limitations remain. First, most of the empirical evidence comes from repeated held-out splits of the target cohort rather than from many fully independent external target cohorts, so external generalizability remains only partially assessed; the JAVELIN Renal 101 application partly mitigates this by providing a randomized prospective trial cohort as the target. Second, the TCGA and FUSCC analyses should be interpreted as retrospective demonstrations of the TransSurv pipeline rather than as deployment-ready clinical prognostic tools, because detailed treatment, surgery, and calendar-time covariates were not uniformly available across studies. Third, finite-sample variation across the 20 replications indicates that across-split instability remains substantial in some targets, especially when cohorts are clinically narrow and event-limited. Finally, the current implementation relies on a Cox model with baseline predictors and linear gene effects; non-proportional hazards, time-varying treatment effects, competing risks, and richer multimodal structure are not yet modeled explicitly. Each of these defines a natural next step, alongside evaluation in additional target cohorts of varying sample size and clinical context, and methods that better accommodate cross-platform measurements. More broadly, the target-centered screening logic should in principle extend to other time-to-event endpoints and to other molecular settings where features can be aligned across cohorts.

Overall, these results establish selective, compatibility-aware transfer as a practical framework for target-cohort survival modeling whenever candidate auxiliary cohorts may contribute compatible prognostic information. The combination of a target-centered Cox specification, an explicit compatibility-screening step, an inspectable disclosure of borrowing, and formal non-asymptotic guarantees positions TransSurv for translational groups that have a small clinically defined target cohort and access to TCGA-scale auxiliary resources: the same pipeline that produces the held-out risk score also produces the disclosure a downstream user can use to decide whether and how to use it. Rather than treating auxiliary cohorts as either fully exchangeable with the target or unusable for borrowing, TransSurv learns when and how to borrow, turning heterogeneous auxiliary cohorts from a source of bias into a source of information for more reliable prognostic modeling in clinically defined oncology populations.

## Materials and methods

### Study design

All analyses followed the same target-centered study design. For each application, one cohort was designated as the target cohort, defined as the population in which prognostic performance was ultimately assessed, and one or more external cohorts were designated as candidate auxiliary cohorts. Performance was always evaluated only in held-out target patients. Within the TransSurv framework, baseline clinical covariates remained target-specific, whereas borrowing was restricted to gene-expression effects from retained auxiliary cohorts. The overall workflow is introduced in Fig. 1; the subsections below specify the cohort definitions, preprocessing steps, target-centered Cox specification, comparators, and evaluation protocol used in each application. Across these applications, the study develops and evaluates target-cohort survival-prediction models using within-target held-out data rather than external cohorts, and is reported in accordance with the TRIPOD+AI reporting guideline for multivariable prediction models [33]; the completed checklist accompanies this submission.

### Cohorts definitions and study endpoints

Throughout, a dataset denotes the parent resource, whereas a cohort denotes an analysis unit defined within a dataset by cancer type and additional clinical restrictions. Within each application, the target cohort is the cohort of primary interest, candidate auxiliary cohorts are the cohorts considered for borrowing, and retained auxiliary cohorts are those kept by the compatibility screening; these role terms are used in this fixed sense throughout. We considered three applications chosen to test clinical realism, breadth across disease contexts, and transfer across independent datasets. The target cohorts, study endpoints, and candidate auxiliary cohorts for each application are summarized in Supplementary Table 2.

For the clinical-trial application, the target cohort was the metastatic renal cell carcinoma population from JAVELIN Renal 101, with PFS as the endpoint. Baseline clinical variables and progression outcomes were obtained from the supplementary clinical dataset (Supplementary Table S11), and gene-expression measurements were obtained from the supplementary TPM expression table (Supplementary Table S13) accompanying the trial report [31]. Candidate auxiliary cohorts were the stage-stratified (I–IV) TCGA KIRC cohorts.

For the TCGA breadth analysis, RNA-seq gene-expression profiles and curated clinical annotations were obtained from the NCI Genomic Data Commons, and overall survival outcomes were taken from a harmonized TCGA clinical outcome resource [7, 8]. Five target cohorts were prespecified across kidney, ovarian, cervical, lung, and breast cancers (Supplementary Table 3), prioritizing three criteria: a treatment pathway expected to be homogeneous within each cohort, an established clinical role for gene-expression prognostic tools in the cancer type, and a common, high-burden patient population. The stage restrictions serve the first, anchoring each cohort to a shared standard-of-care pathway so that a prognostic score does not conflate prognosis with treatment choice. The second is supported by guideline-endorsed expression assays in hormone receptor–positive breast cancer [1, 4, 34] and by validated prognostic signatures in clear-cell renal cell carcinoma [35], lung adenocarcinoma [36], and high-grade serous ovarian cancer [37]. For each target, candidate auxiliary cohorts consisted of (i) alternative clinically defined subcohorts within the same cancer type and (ii) one auxiliary cohort for each of the remaining TCGA cancer types.

For the cross-dataset application, the target cohort was the FUSCC TNBC cohort, restricted to ER/PR/HER2-negative disease according to the published receptor-status criteria [32]. Published FUSCC resources provided gene-expression measurements and baseline clinical covariates [32, 38], whereas recurrence outcomes were obtained directly from the study investigators and were not part of the public transcriptomic release. Candidate auxiliary cohorts were TNBC cohorts constructed from TCGA and METABRIC, with receptor-status definitions aligned to the target analysis. This application intentionally combined sequencing-based target and auxiliary transcriptomes (FUSCC and TCGA) with a microarray-derived auxiliary cohort (METABRIC), thereby introducing substantially stronger measurement heterogeneity than in the TCGA-only analyses. METABRIC molecular and clinical data were obtained from the established resource and follow-up releases [39, 40].

### Predictor definition and cohort-specific preprocessing

Predictors comprised prespecified baseline clinical covariates together with high-dimensional gene-expression features. Baseline clinical covariates were application-specific and remained target-specific throughout the transfer procedure. These included age, sex, treatment arm, and the binary PDL1FL signature for JAVELIN Renal 101; age and pathologic stage for the TCGA target-cohort analyses, with menopausal status added for BRCA and age only for OV; and age and stage for the FUSCC TNBC analysis. Categorical baseline covariates were represented using indicator variables.

Gene-expression predictors were defined by aligning gene identifiers across the target cohort and its candidate auxiliary cohorts within each application, and retaining only the intersected feature set shared by all cohorts entering that analysis. This yielded *18,166* aligned gene-expression features for the JAVELIN Renal 101 analysis, *20,531* for the TCGA target-cohort analyses, and *16,618* for the FUSCC–TCGA–METABRIC TNBC analysis. Cohort sizes and event counts are summarized in Supplementary Section 2.3.

Preprocessing was then performed separately within each cohort. Continuous predictors, including gene-expression measurements and numeric baseline covariates, were transformed feature-wise within cohort using a rank-based inverse-normal transformation. Specifically, if $r_{ij}$ denotes the within-cohort rank of feature *j* for subject *i* among *n* subjects, then the transformed value was 


\begin{align*} & \tilde{x}_{ij} = \Phi^{-1}\!\left\{ \mathrm{clip}\!\left(\frac{r_{ij}+0.5}{n},\, \varepsilon,\, 1-\varepsilon\right) \right\}\kern-2pt , \end{align*}

where $\Phi ^{-1}$ is the standard normal quantile function and $\varepsilon =10^{-6}$ prevents numerical infinities at the boundaries. This monotone transformation preserves within-cohort ordering while reducing sensitivity to heavy tails, outliers, and study-specific scale differences. Applied feature-wise within each cohort, the transformation also makes the marginal distribution of every gene essentially identical across cohorts by construction, so the covariate shift remaining after preprocessing is confined to the dependence structure among genes; this residual shift is what the statistical guarantees quantify (Supplementary Assumption 4 and Theorem 1). A sensitivity comparison with within-cohort z-score normalization is reported in Supplementary Section 4.1. Missing continuous predictor values were imputed by within-cohort medians; a sensitivity analysis using a cross-predictor imputation strategy is reported in Supplementary Section 4.2. Genes with zero variance in the relevant training data were removed before model fitting.

To avoid information leakage, all preprocessing quantities for the target cohort were estimated using only the target training split and were then applied unchanged to the corresponding held-out target test split. Candidate auxiliary cohorts were preprocessed within cohort before screening and model fitting, because they contributed information only through the training-side transfer routine.

### Target-centered transfer, compatibility screening and final model fitting

Within the TransSurv framework, prognosis is modeled through a target-centered Cox specification with prespecified baseline clinical covariates and high-dimensional gene-expression predictors. Baseline clinical effects remain target-specific, whereas borrowing across cohorts is restricted to the gene-expression component. The aim is to improve estimation of the target genomic effect by borrowing selectively from auxiliary cohorts whose fitted prognostic structure is sufficiently compatible with the target.

Index the target cohort by *0*, and let $T_{i}^{(0)}$, $B_{i}$ and $X_{i}$ denote the survival time, baseline clinical covariates and aligned gene expressions vector for target patient *i*. We model the target survival probability $S^{(0)}(t \mid B_{i}, X_{i})$ through a Cox proportional hazard model [41], shown by Equation (1). 


(1) \begin{align*} \begin{split} S^{(0)}(t \mid B_{i}, X_{i}) &= \Pr\bigl(T_{i}^{(0)}> t \mid B_{i}, X_{i}\bigr) = \exp\!\left\{ -\int_{0}^{t} \lambda^{(0)}(u \mid B_{i}, X_{i})\,du \right\}\kern-2pt , \\ \lambda^{(0)}(t \mid B_{i}, X_{i}) &= \lambda^{(0)}_{0}(t)\exp\!\left(B_{i}^\top \alpha^{(0)} + X_{i}^\top \omega_{0}\right) \end{split}\end{align*}

The target survival model is therefore determined by the baseline hazard $\lambda ^{(0)}_{0}(t)$, the target-specific clinical coefficients $\alpha ^{(0)}$, and the target gene-expression effect $\omega _{0}$. For each candidate auxiliary cohort *k*, we allow the baseline hazard to remain cohort-specific, but write the gene-expression effect relative to the target as 


\begin{align*} & \omega^{(k)} = \omega_{0} + \delta_{k}, \end{align*}

where the $\delta _{k}$ are *contrast* vectors that capture the cohort-specific deviations from the target gene-expression effect. We assume both $\omega _{0}$ and the $\delta _{k}$ to be sparse, so that most prognostic gene-expression effects are zero and only a subset of genes requires cohort-specific correction. Figure 5a illustrates this decomposition schematically.

**Figure 5:**
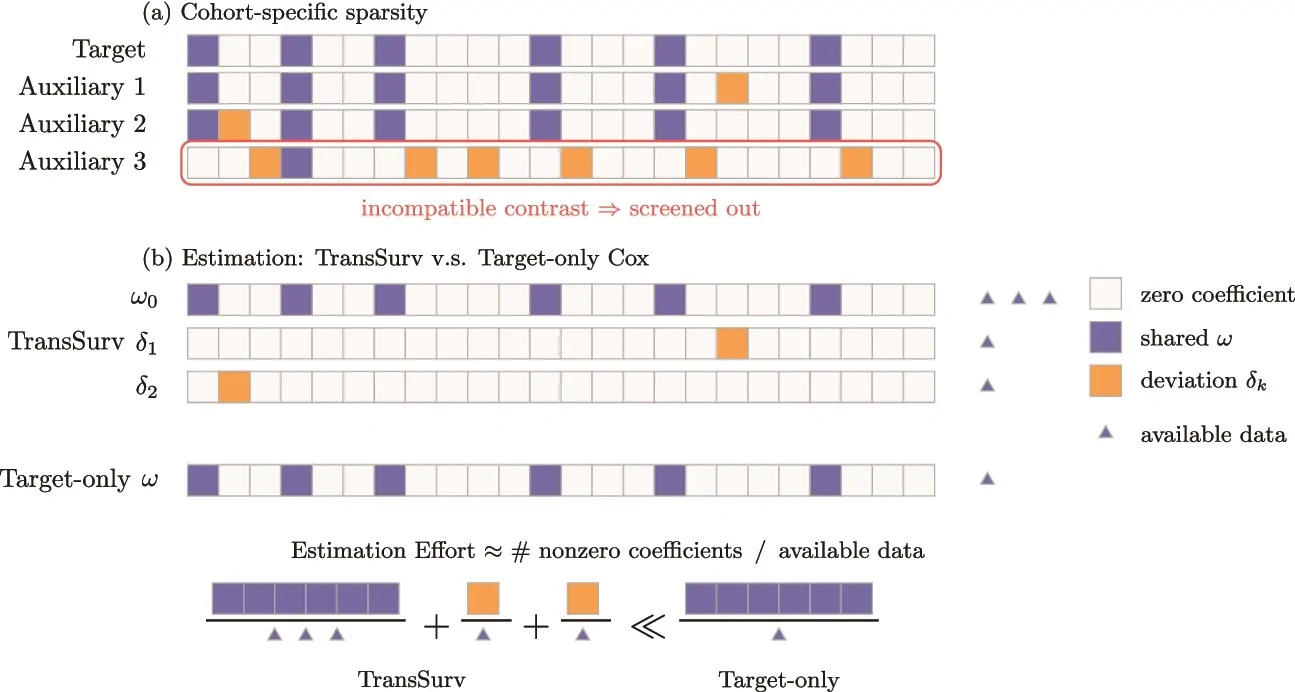
Schematic illustration of target-centered borrowing in TransSurv, in which squares mark nonzero shared target effects, auxiliary-specific deviations or zero coefficients (see key): (a) example of shared and cohort-specific gene-expression effects across one target cohort and three candidate auxiliary cohorts, where compatible auxiliary cohorts share most target-associated nonzero effects whereas an auxiliary cohort with large contrast is screened out; (b) intuition for post-screening joint fitting, where each triangle represents the data available from one cohort and the shared target-centered effect $\omega _{0}$ is estimated using event information from three cohorts, whereas the contrast terms $\delta _{k}$ are much sparser and estimated only where auxiliary-specific correction is needed, so that the joint effort required to estimate $\omega _{0}$ and $\delta _{k}$ remains smaller than that needed for target-cohort-only estimation.

The next step of TransSurv is compatibility screening. For each candidate auxiliary cohort *k*, a pilot fit yields an estimated contrast vector $\hat{\delta }_{k}$. We then define the fitted contrast size 


\begin{align*} &D_{k} = \|\hat{\delta}_{k}\|_{1}.\end{align*}

Smaller values of $D_{k}$ indicate closer agreement with the target in fitted gene–survival relations. Candidate auxiliary cohorts are ranked by $D_{k}$, from smallest to largest, and the retained set $\hat{\mathcal{A}}$ consists of the first *K* cohorts in this ranking. The count *K* is chosen on the target training data, increasing along the ranking for as long as the training-side selection criterion improves. Cutting the ranked list at *K* is the same as thresholding $D_{k}$ at its *K*th smallest value, so the procedure searches over the same threshold rules analyzed in the theory, with their unknown constants replaced by a single interpretable tuning parameter. Under the conditions of Supplementary Proposition 1, the ranking places every oracle-compatible cohort ahead of every incompatible one with probability tending to one, so the nested sets it induces contain the oracle set. Figure 5a illustrates this compatibility-screening step as well.

Given the retained auxiliary cohorts and the target cohort, the target-centered Cox model estimates $\omega _{0}$ and the corresponding set of contrasts $\{\delta _{k}\}$ by minimizing the summed cohort-specific Cox partial losses, 


\begin{align*} (\hat{\omega}_{0},\{\hat{\delta}_{k}, \hat{\alpha}^{(k)}\}_{k\in \hat{\mathcal{A}}}) =& \, \arg\min_{\omega_{0},\{\delta_{k}, \alpha^{(k)}\}} \left\{ \ell_{0}(\alpha^{(0)}, \omega_{0}) + \sum_{k\in \hat{\mathcal{A}}}\ell_{k}(\alpha^{(k)}, \omega_{0}+\delta_{k}) \right. \\ & \left. + \lambda_\omega\|\omega_{0}\|_{1} + \sum_{k\in\hat{\mathcal{A}}}\lambda_{\delta,k}\|\delta_{k}\|_{1} \right\}\kern-2pt , \end{align*}

where $\ell _{k}$ is the cohort-specific Cox partial loss function. We add $\ell _{1}$ regularization to the target gene-expression effects $\omega _{0}$ [42] and each auxiliary-specific deviation $\delta _{k}$, which encodes the sparseness assumption. Here $\lambda _\omega $ controls sparsity of the shared target-centered gene-expression effect, and $\lambda _{\delta ,k}$ controls the extent of cohort-specific correction for auxiliary cohort *k*. These auxiliary-specific penalties were allowed to differ across cohorts when the expected auxiliary–target mismatch differed materially. In the FUSCC TNBC application, we penalized the TCGA deviation fourfold more strongly than the METABRIC deviation, that is, $\lambda _{\delta ,\mathrm{TCGA}} = 4\,\lambda _{\delta ,\mathrm{METABRIC}}$, so that the more distinct METABRIC auxiliary could accommodate larger cohort-specific correction. The target clinical coefficients $\alpha ^{(0)}$ are estimated only from the target cohort and are left unregularized, as they represent a small set of target-specific clinical variables with dense effects.

Figure 5b illustrates the benefit of post-screening joint fitting. The shared target gene-expression effect is estimated using event information from both the target and the retained auxiliary cohorts, whereas the contrast terms are typically much sparser and therefore easier to estimate. The same representation also clarifies why compatibility screening is essential: when an auxiliary cohort shows large systematic disagreement with the target, the corresponding contrast $\delta _{k}$ becomes less sparse and the gain from borrowing is reduced.

In our analysis, all model tuning decisions were made using only the target training split. Technical details, statistical guarantees, and implementation details for the target-centered Cox model are provided in Supplementary Sections 1, 6, and 7.

### Comparator models

TransSurv was compared against prespecified baselines chosen to represent no borrowing, unscreened borrowing, and contemporary multi-study and transfer-learning survival methods. The *target-only* comparator was an $\ell _{1}$-penalized Cox model fitted using only the target training split, with the same baseline clinical covariates, aligned gene-expression predictors, preprocessing steps, and target-side tuning protocol as TransSurv. As a sensitivity analysis, the target-only comparator was additionally refit in the JAVELIN and FUSCC applications on all post-quality-control target-cohort genes rather than the intersected gene set; the refit changed its held-out C-index by *-0.001* in JAVELIN and *+0.016* in FUSCC, and TransSurv retained its advantage in both (Supplementary Section 4.3). When the target and auxiliary cohorts shared a directly matched disease context, we additionally evaluated *naive-pooling*, in which the target cohort and all candidate auxiliary cohorts were combined into a single penalized Cox model without compatibility screening.

We also compared against *MetaSurv* as a representative multi-study survival baseline [30], which does not screen auxiliary cohorts for target-compatibility before borrowing. MetaSurv was implemented using the software provided by the original authors through their public repository. The same target cohort, candidate auxiliary cohorts, preprocessing, and held-out target evaluation protocol were used as in the corresponding TransSurv analysis. Hyperparameter tuning followed the strategy described in the original MetaSurv paper, while the candidate hyperparameter set was adapted in a minimal way to reflect the size of the target training split in each application. These adjustments were limited to avoiding settings that were infeasible given the available training-split sample size and did not alter the underlying MetaSurv model or training procedure.

We additionally compared against the shared-support $\ell _{2,1}$-regularized Cox transfer model of Li *et al.* [27] as a Cox-based transfer baseline defined at transcriptome scale, which couples source and target coefficients through a row-wise $\ell _{2,1}$ penalty and likewise applies no compatibility screening. Because it is a single-source design, the within-disease auxiliary cohorts served as its source domain in each application (Supplementary Section 7.3). The comparator was implemented in our own code following the published formulation, and the same preprocessing, splits, and held-out target evaluation protocol were used as in the corresponding TransSurv analysis, with the penalty scale tuned per split on the target training data.

### Held-out evaluation

All real-data evaluations followed the same target-cohort protocol. For each application, we generated 20 random 80%/20% train–test splits of the target cohort. All data-adaptive steps were restricted to the target training split, including target-side preprocessing, auxiliary-cohort screening, retained-set selection, tuning, and model fitting. Performance was evaluated only in held-out target patients and summarized as mean *± * s.d. across replications.

Discrimination was assessed using the concordance index (C-index) and the time-dependent AUC at two prespecified cohort-specific horizons. Exact evaluation horizons for the TCGA and FUSCC analyses are listed in Supplementary Table 1; the JAVELIN horizons were 3 and 6 years. These horizons were chosen to reflect clinically relevant landmark times at which risk discrimination is commonly assessed. In the JAVELIN application, within-arm risk stratification was examined separately; details are given in the interpretability analyses subsection.

### Interpretability analyses

To summarize how transfer operated in fitted models, we used two complementary displays of the estimated target-centered gene-expression structure. For compact main-text visualization, we constructed radial coefficient–deviation plots from representative target train–test splits using the 50 genes with the largest absolute target-cohort coefficients. To aid interpretation, these genes were grouped into broad lineage- and biologic-state–associated programs, guided by pan-cancer cell-of-origin structure and compact hallmark-style expression programs [5, 43]. Within each display, the target-cohort gene-expression effects $\hat{\omega }_{0}$ and the auxiliary-specific deviation terms $\hat{\delta }_{k}$ were shown jointly so that dominant target-associated signals and localized cohort-specific corrections could be read together. More detailed rectangular coefficient–deviation heatmaps, using the 100 genes with the largest absolute target-cohort coefficients or all nonzero target-cohort genes when fewer than 100 were selected, were retained in the Supplementary Information as reference versions.

We also summarized auxiliary-cohort compatibility across replications using the fitted contrast magnitudes $D_{k}$ together with auxiliary retention frequencies. In the main text, these quantities were displayed as compact compatibility landscapes that emphasize the recurrent compatibility structure among retained auxiliary cohorts. The Supplementary Information retains the corresponding target-specific compatibility profiles for all candidate auxiliary cohorts, including screened-out cohorts, and reports the across-replication variability of the compatibility estimates as mean *± * SD over the 20 replications.

For the JAVELIN Renal 101 application, we additionally examined within-arm prognostic stratification. For each fitted model, low- and high-risk groups were defined by k-means clustering (*k=2*) applied to predicted event probabilities at the prespecified evaluation horizons together with the model-derived risk score, and Kaplan–Meier curves were then plotted separately within each treatment arm for a representative held-out split.

**Key Points**

- TransSurv screens candidate auxiliary cohorts for empirical compatibility before borrowing, so incompatible external data does not erode target-cohort discrimination; non-asymptotic guarantees formalize when compatible cohorts help and when screening protects the target fit.
- Borrowing is target-centered: clinical effects stay cohort-specific, transfer is restricted to gene-expression effects, and retained auxiliary cohorts may carry sparse cohort-specific deviations.
- TransSurv improved or preserved held-out discrimination over target-only fitting, naive pooling, MetaSurv, and an $\ell _{2,1}$-regularized Cox transfer model across three transfer regimes: a randomized clinical trial (JAVELIN Renal 101), five clinically defined TCGA cohorts, and a cross-platform triple-negative breast cancer analysis.
- TransSurv reports which auxiliary cohorts were retained and how much each contributed, making the source and extent of borrowing visible.

## Supplementary Material

TransSurv_SI_bbag532

## Data Availability

The JAVELIN Renal 101 supplementary clinical and gene-expression data used in this study are available with the original publication [31]. TCGA molecular and clinical data are publicly available through the NCI Genomic Data Commons, and harmonized TCGA survival outcomes are available through the TCGA clinical outcome resource [7, 8]. METABRIC molecular and clinical data are available through the published METABRIC resources [39, 40]. Published FUSCC TNBC transcriptomic measurements and baseline clinical covariates are available through the original FUSCC publications [32, 38]. The FUSCC recurrence outcomes used in this study were obtained directly from the study investigators and are not part of the public transcriptomic release; these data are therefore not publicly redistributed as part of this work. Data-requirement notes for obtaining or reconstructing the publicly available analysis datasets are provided with the analysis-replay code described below. All analyses were conducted in Python 3.12.3. The analysis code fixes random seeds and records the tuning parameters, model settings, and preprocessing steps used in each application. The target-centered Cox model implementation and simulation code are available as a standalone Python package at https://anonymous.4open.science/r/tc_cox-E316/. The analysis-replay code reproducing the JAVELIN Renal 101, TCGA, and FUSCC TNBC analyses and manuscript figures is available at https://anonymous.4open.science/r/tccox-paper-replay-4F4E/.
