# Supplementary material for: TransSurv: compatibility-aware transfer learning for target-cohort survival prediction
Source: Brief Bioinform. 2026 Oct 27;27(5):bbag532. doi: 10.1093/bib/bbag532 (PMC13710770; doi:10.1093/bib/bbag532)
Supplement: Supplementary file 1 [file transsurv_si_bbag532.pdf]

Supplementary Information for
TransSurv: compatibility-aware transfer learning for target-cohort survival prediction

Note to reviewers and editors: changes made in this revision are shown in blue. Each substantive change is preceded by a short bracketed label printed in blue, and the point-by-point response cites these labels, so any change can be located by searching this file for the label given in the response. Labels are shared with the revised manuscript. Revised supplementary sections, tables, and figures are cited by number instead. This note and all colour markup will be removed at acceptance.

1 Technical details of the target-centered Cox model in TransSurv

This section gives the technical formulation of the target-centered Cox model used by TransSurv and the practical screening rule used to retain compatible auxiliary cohorts. To keep the notation focused, we write the model only for the transferred high-dimensional covariate block X. Low-dimensional unpenalized baseline covariates can be added additively, as noted in the solver remarks, without changing the target-centered decomposition or the screening logic below.

Assume that there are $K+1$ study populations, consisting of one *target* cohort ($k = 0$) and K candidate *auxiliary* cohorts ($k = 1, \ldots, K$). Cohort k provides i.i.d. observations

$$\{(T_i^{(k)}, \Delta_i^{(k)}, X_i^{(k)})\}_{i=1}^{n_k},$$

where $T_i^{(k)}$ is the observed follow-up time, $\Delta_i^{(k)} \in \{0,1\}$ is the event indicator, and $X_i^{(k)} \in \mathbb{R}^p$ is the aligned covariate vector. Each cohort follows a Cox proportional hazards model

$$\lambda^{(k)}(t \mid X^{(k)}) \;=\; \lambda_0^{(k)}(t)\, \exp\{(X^{(k)})^\top \omega^{(k)*}\}, \tag{1}$$

where $\lambda_0^{(k)}$ is an unspecified cohort-specific baseline hazard and $\omega^{(k)*} \in \mathbb{R}^p$ is the cohort-specific log-hazard ratio vector. Thus, both baseline risk and covariate distribution are allowed to vary across cohorts. We write $\Sigma^{(k)} = \mathrm{Cov}(X^{(k)})$ for the cohort-specific covariance matrix; the statistical-guarantees section allows heterogeneous $\Sigma^{(k)}$ under regularity conditions. Throughout, covariates are understood to be centered within cohort. Our target is the target-cohort coefficient vector $\omega^{(0)*}$.

Target-centered joint estimation TransSurv is target-centered in the sense that each auxiliary-cohort coefficient is written as the target coefficient plus a cohort-specific deviation,

$$\delta^{(k)*} := \omega^{(k)*} - \omega^{(0)*}, \qquad k = 1, \ldots, K.$$

If $\delta^{(k)*} = 0$, then auxiliary cohort k shares the target log-hazard ratio exactly, and pooled fitting is efficient. If $\delta^{(k)*}$ is large, naive pooling can bias the target estimate. The role of TransSurv is

therefore twofold: first, identify a subset of compatible auxiliary cohorts; second, fit a joint estimator that borrows the shared signal while allowing sparse cohort-specific correction. In the technical development below, this compatible subset is denoted by the *transferable set* $C \subseteq \{1, \ldots, K\}$. Figure 5 in the main text illustrates this decomposition: panel (a) shows the target-centered split of each cohort coefficient into a shared target effect plus a sparse cohort-specific contrast and depicts compatibility screening, while panel (b) sketches the post-screening joint estimation in which the contrasts are absorbed into sparse correction terms.

Condition on a fixed transferable set C. The corresponding target-centered Cox estimator solves

$$(\widehat{\omega}_0, \{\widehat{\delta}^{(k)}\}_{k \in C}) \in \arg \min_{\omega_0, \{\delta^{(k)}\}_{k \in C}} \left\{ \ell_0(\omega_0) + \sum_{k \in C} \ell_k\big(\omega_0 + \delta^{(k)}\big) + \lambda_0 \|\omega_0\|_1 + \sum_{k \in C} \lambda_k \, \|\delta^{(k)}\|_1 \right\}, \qquad (2)$$

where λ_0 controls sparsity in the shared target-centered coefficient and λ_k controls the size of the auxiliary-specific correction for cohort k. The cohort-specific negative Cox partial log-likelihood is

$$\ell_k(\omega) \;=\; -\frac{1}{n_k} \sum_{i=1}^{n_k} \Delta_i^{(k)} \left\{ X_i^{(k)\top} \omega - \log \sum_{j=1}^{n_k} Y_j^{(k)}(T_i^{(k)}) \, \exp\big(X_j^{(k)\top} \omega\big) \right\}, \qquad (3)$$

with $Y_j^{(k)}(t) = 1\{T_j^{(k)} \geq t\}$ the at-risk indicator.

The objective in (2) is jointly convex in $(\omega_0, \{\delta^{(k)}\}_{k \in C})$. Its interpretation is straightforward: the shared vector ω_0 borrows information across the target and retained auxiliary cohorts, whereas the sparse deviations $\delta^{(k)}$ absorb residual cohort-specific disagreement and thereby protect the target estimate from harmful transfer. When retained auxiliary cohorts are close to the target, their fitted deviations are shrunk toward zero; when limited disagreement remains, it is represented by sparse corrections rather than forced into a pooled coefficient. The formal rate guarantee for this estimator is given later in Theorem 1. In particular, that theorem makes precise the favorable regime in which the leading estimation term behaves as if the effective sample size were approximately

$$n_0 + \sum_{k \in C} n_k,$$

up to explicit terms reflecting residual auxiliary–target mismatch and cohort heterogeneity.

Compatibility screening In practice the transferable set C is unknown. TransSurv therefore estimates compatibility from an initial joint fit over the target cohort and all candidate auxiliary cohorts. For each candidate auxiliary cohort k, define the fitted *contrast size*

$$D_k \;:=\; \|\widehat{\delta}^{(k)}\|_1$$

and the *information proxy*

$$I_k \;:=\; \frac{1}{n_k} \sum_{i=1}^{n_k} \Delta_i^{(k)},$$

the observed event fraction in cohort k. Small D_k indicates that the fitted auxiliary effect is close to the target-centered effect after regularization, whereas I_k excludes cohorts that are too event-poor to contribute stable transfer even if they appear compatible.

The practical screening-and-refitting routine is summarized in Algorithm 1.

Algorithm 1 Auxiliary-cohort screening for the target-centered Cox model

Require: Datasets $\{(T^{(k)}, \Delta^{(k)}, X^{(k)})\}_{k=0}^{K}$; penalties $\lambda_0 \asymp \sqrt{\log p/n_0}$ and $\{\lambda_k \asymp \sqrt{\log p/n_k}\}_{k=1}^{K}$; thresholds $c_1 > 0$, $t_2 \in (0,1)$

Ensure: Final coefficients $\widehat{\omega}_0$ and $\{\widehat{\delta}^{(k)}\}_{k\in\widehat{C}}$; per-cohort coefficients $\widehat{\omega}^{(k)} = \widehat{\omega}_0 + \widehat{\delta}^{(k)}$

$C \leftarrow \{1, 2, \ldots, K\}$ ▷ initialize candidate auxiliary cohorts

Initial fit: solve (2) on $\{0\} \cup C$ to obtain $(\widehat{\omega}_0, \{\widehat{\delta}^{(k)}\}_{k\in C})$

for $k \in C$ **do**

  $D_k \leftarrow \|\widehat{\delta}^{(k)}\|_1, \quad I_k \leftarrow n_k^{-1} \sum_{i=1}^{n_k} \Delta_i^{(k)}$

end for

$\widehat{C} \leftarrow \{\, k \in C \, : \, D_k \le c_1(\lambda_0 + \lambda_k) \ \wedge \ I_k \ge t_2 \,\}$

Refit: solve (2) on $\{0\} \cup \widehat{C}$ with the same $(\lambda_0, \{\lambda_k\})$ to obtain the final $(\widehat{\omega}_0, \{\widehat{\delta}^{(k)}\}_{k\in\widehat{C}})$

return $\widehat{\omega}_0$, $\{\widehat{\delta}^{(k)}\}_{k\in\widehat{C}}$, and $\{\widehat{\omega}^{(k)}\}_{k\in\{0\}\cup\widehat{C}}$

The threshold c_1 compares the fitted contrast size to the combined stochastic scale of the shared fit and the cohort-specific fit. Requiring

$$D_k \le c_1(\lambda_0 + \lambda_k)$$

therefore retains auxiliary cohorts whose estimated deviation is no larger than the effective noise floor of the problem. The threshold t_2 plays a complementary role: even a cohort that appears compatible contributes little if it contains too few observed events to support stable borrowing. The asymptotic justification of this screening rule is given in Theorem 2.

In applications, c_1 and t_2 are best viewed as theoretical devices rather than user-facing tuning parameters. A convenient implementation is to rank candidate auxiliary cohorts by D_k and treat the number of retained cohorts as a tuning parameter selected on the target training split, increasing the count along the ranking while the training-side selection criterion improves. **[E8]** Cutting the ranked list at any retained count is the same as thresholding D_k at the corresponding order statistic, so the retained-count search ranges over exactly the thresholding rules; under the conditions of Proposition 1, the event-gated ranking places every oracle-compatible cohort ahead of every incompatible one with probability tending to one, so this nested search space contains the oracle set. The finite-sample behavior of these ideas is examined in Section 5, where compatible auxiliary cohorts reduce estimation error and fitted contrast magnitudes more reliably separate compatible from incompatible cohorts as sample size increases.

2 Additional figures and supporting results

2.1 AUC horizons by cohort

We selected cohort-specific AUC horizons (t_1, t_2) to align with standard clinical landmark reporting and to avoid evaluation in the extreme right tail with few patients at risk. For locally advanced cervical cancer and resectable stage I lung adenocarcinoma, we used 3- and 5-year OS landmarks that are commonly reported in definitive chemoradiation and surgical studies [Lorusso et al., 2024, McCormack et al., 2024, Guerrera et al., 2015]. For HR+/HER2– breast cancer and stage I–III RCC, we extended the second horizon to 6 years to reflect persistent medium-term risk in long-term follow-up studies and population survival summaries [Pan et al., 2017, Sparano et al., 2018, National Cancer Institute, Surveillance Research Program, 2025]. For stage IIIC ovarian cancer and TNBC RFS, we used 2/4 years and 0.5/1.5 years, respectively, to bracket the typical median

survival or early-relapse window reported in frontline trials and recurrence-pattern studies [Tewari et al., 2019, Dent et al., 2007].

Supplementary Table 1: Cohort-specific AUC horizons used for target cohorts in Table 2 and the FUSCC cohort.

Target cohort	t_1 (years)	t_2 (years)
BRCA HR+/HER2–, Stage I–III	*3*	*6*
CESC Stage IIB–IVA	*3*	*5*
KIRC Stage I–III	*3*	*6*
LUAD Stage I	*3*	*5*
OV Stage IIIC	*2*	*4*
FUSCC TNBC	*0.5*	*1.5*

2.2 Study design summary

Table 2 summarizes the target cohorts, study endpoints, and candidate auxiliary cohorts used in the three real-data applications of TransSurv.

Supplementary Table 2: Target cohorts, study endpoints, and candidate auxiliary cohorts in the TransSurv applications.

Target cohort definition	Outcome	Candidate auxiliary cohorts
JAVELIN Renal 101 metastatic renal cell carcinoma cohort	PFS	Stage-stratified (I–IV) TCGA kidney renal clear cell carcinoma (KIRC) cohorts
Five clinically defined TCGA target cohorts in Table 3	OS	(i) alternative clinically defined subcohorts within the same cancer type; and (ii) one auxiliary cohort for each of the remaining TCGA cancer types
FUSCC TNBC target cohort	RFS	TNBC cohorts from TCGA and METABRIC

2.3 Basic cohort summaries

The tables in this subsection summarize precise target cohort definitions for the TCGA application, together with cohort sizes and event counts for the target and candidate auxiliary cohorts entering each real-data application. They justify the target cohort selection logic for the TCGA application and provide the sample-size and event-information context for model-building/ held-out performance results in the main text and for the auxiliary-cohort screening step.

Supplementary Table 3: Clinical context of the five prespecified TCGA target cohorts used in the breadth analysis.

Cancer type	Target cohort restriction (TCGA)	Typical primary treatment context (reference)
KIRC	Clear-cell RCC; stage I–III	Nephrectomy for localized disease Powles et al. [2024]
OV	High-grade serous ovarian carcinoma; stage IIIC	Cytoreductive surgery with platinum–taxane chemotherapy Armstrong et al. [2022]
CESC	Cervical squamous cell carcinoma and endocervical adenocarcinoma; stage IIB–IVA	Definitive chemoradiation with weekly cisplatin Abu-Rustum et al. [2023]
LUAD	Lung adenocarcinoma; stage I	Surgery as primary therapy for stage I disease Postmus et al. [2017]
BRCA	HR+/HER2– breast cancer; stage I–III	Surgery with endocrine therapy, chemotherapy guided by clinical/genomic risk Sparano et al. [2018]

Supplementary Table 4: JAVELIN Renal 101 metastatic renal cell carcinoma (PFS): cohort sizes and event counts.

Group	Cohort name	N_{total}	N_{event}
Target	JAVELIN Renal 101 cohort	726	358
TCGA KIRC auxiliary	Stage I	293	31
	Stage II	68	16
	Stage III	139	58
	Stage IV	101	87

Supplementary Table 5: TCGA target cohorts (OS): cohort sizes and event counts.

Target cohort	N_{total}	N_{event}
KIRC Stage I–III	500	119
OV Stage IIIC	246	153
CESC Stage IIB–IVA	139	36
LUAD Stage I	307	75
BRCA HR+/HER2–, Stage I–III	477	50

Supplementary Table 6: TCGA candidate auxiliary cohort sizes and event counts.

Auxiliary cohort	N_{total}	N_{event}
BRCA (alternate)	713	136
CESC (alternate)	163	37
LUAD (alternate)	260	136
KIRC (alternate)	103	82
OV (alternate)	61	31
BLCA	425	189
COAD	480	107
ESCA	190	80
GBM	168	135
HNSC	566	253
KIRP	305	46
LAML	173	114
LGG	533	136
LIHC	392	143
LUSC	549	246
MESO	87	74
PAAD	180	95
SARC	265	99
SKCM	427	198
STAD	424	160
UCEC	555	91

Supplementary Table 7: FUSCC TNBC (RFS): cohort sizes and event counts

Dataset	N_{total}	N_{event}	Stage	N_{stage}	$N_{\text{stage event}}$
Metabric	230	94	I	68	19
			II	135	55
			III	27	20
TCGA	171	30	I	32	3
			II	112	13
			III	27	14
FUSCC	357	49	I	90	4
			II	217	25
			III	50	20

2.4 Coefficient–deviation heatmaps under TransSurv framework

To illustrate how TransSurv borrows across cohorts, we show coefficient–deviation heatmaps from representative fitted models for each target cohort. In each heatmap, columns correspond to the 100 genes with the largest absolute target-cohort coefficients in the displayed split. The top row shows the estimated target-cohort gene-expression effects, and each subsequent row shows the auxiliary-specific deviation term for a retained auxiliary cohort relative to the target. Colors indicate signed coefficients after within-figure rescaling to $[-1, 1]$, and white indicates coefficients estimated as zero. These plots are descriptive summaries of the fitted transfer structure rather than formal

gene-ranking results.

2.4.1 JAVELIN Renal 101 cohort

The figure below summarizes the fitted transfer structure for the JAVELIN Renal 101 trial cohort with stage-stratified TCGA KIRC auxiliaries.

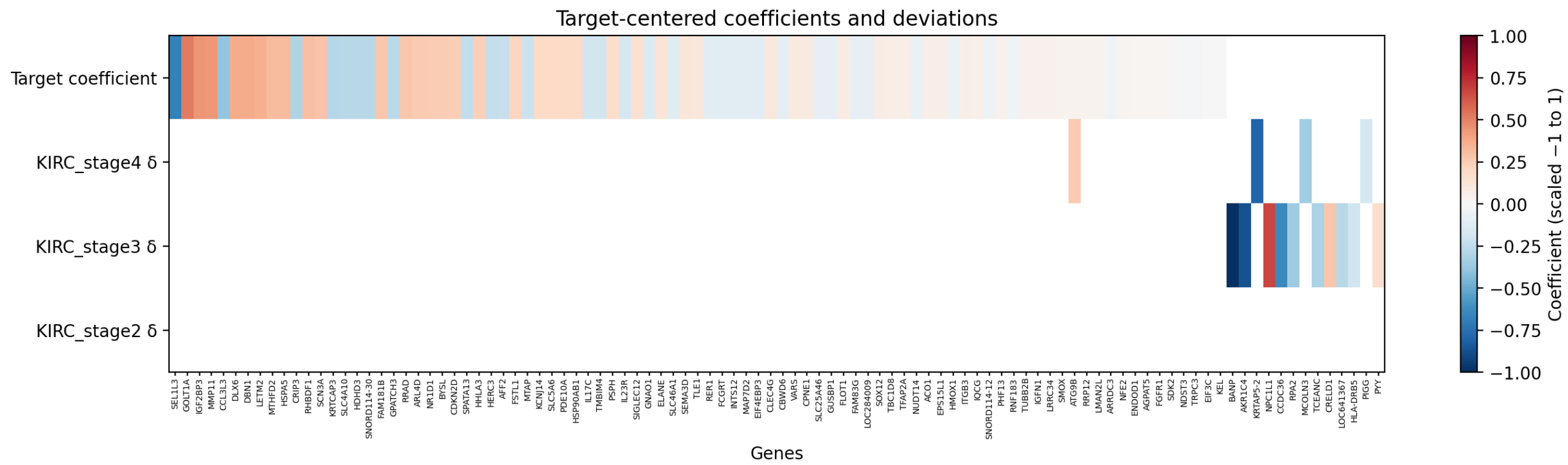

Supplementary Figure 1: Representative coefficient-deviation heatmap for the JAVELIN Renal 101 target cohort. Columns show the 100 genes with the largest absolute target-cohort coefficients. The top row shows estimated target-cohort gene effects; subsequent rows show auxiliary-specific deviations relative to the target. White indicates coefficients/deviations estimated as zero.

2.4.2 TCGA Cohorts

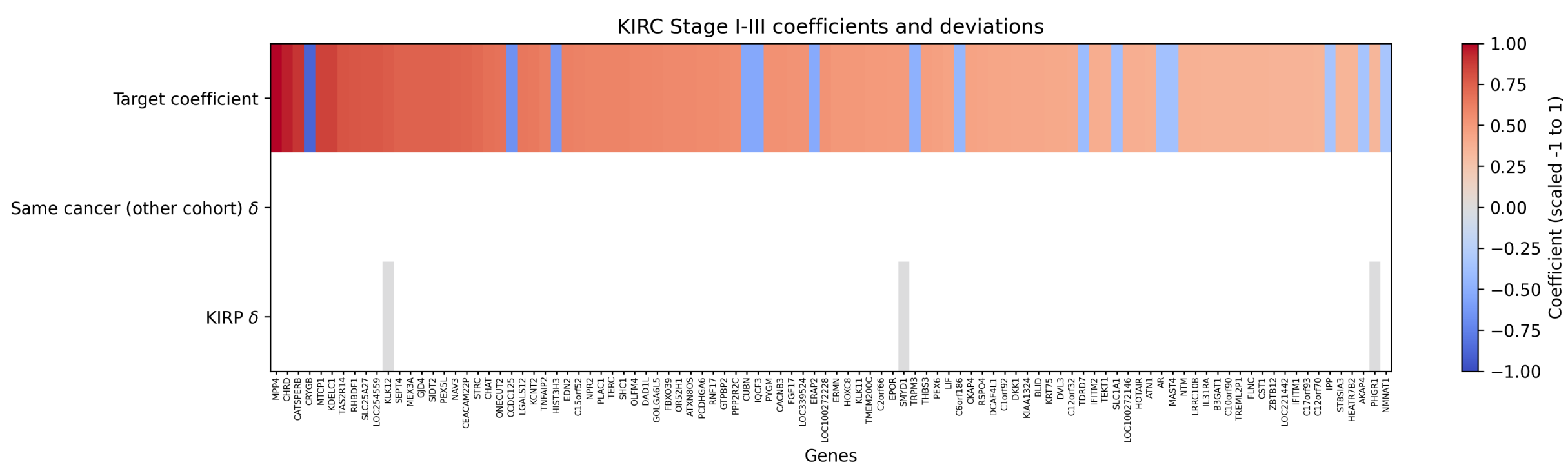

Supplementary Figure 2: Representative TransSurv coefficient–deviation heatmap for the KIRC stage I–III target cohort.

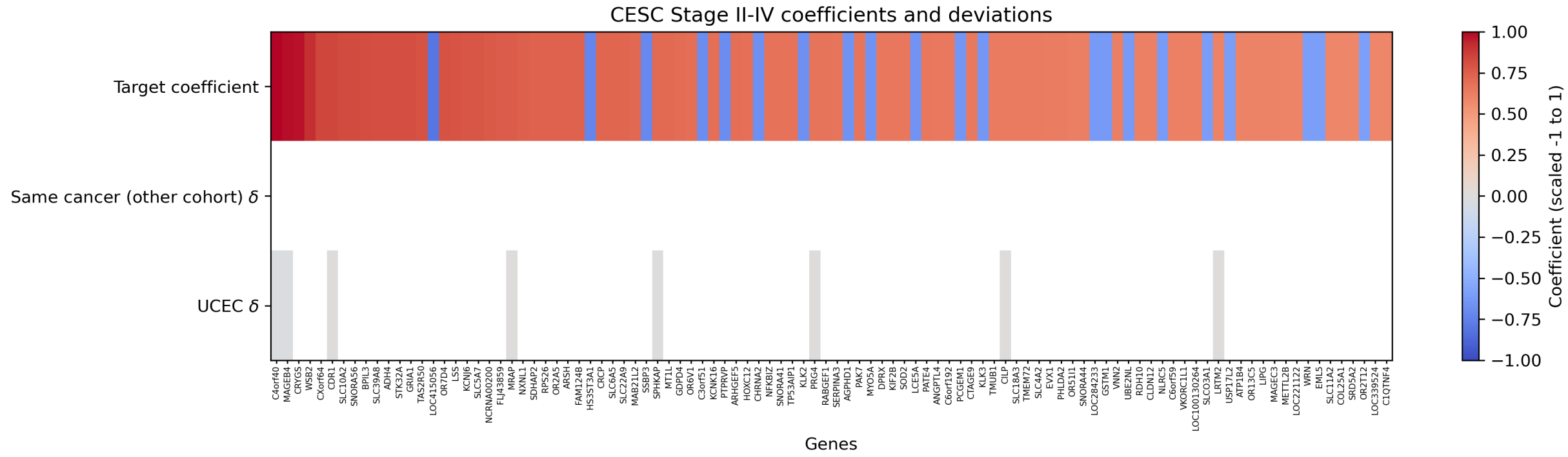

Supplementary Figure 3: Representative TransSurv coefficient–deviation heatmap for the CESC stage IIB–IVA target cohort.

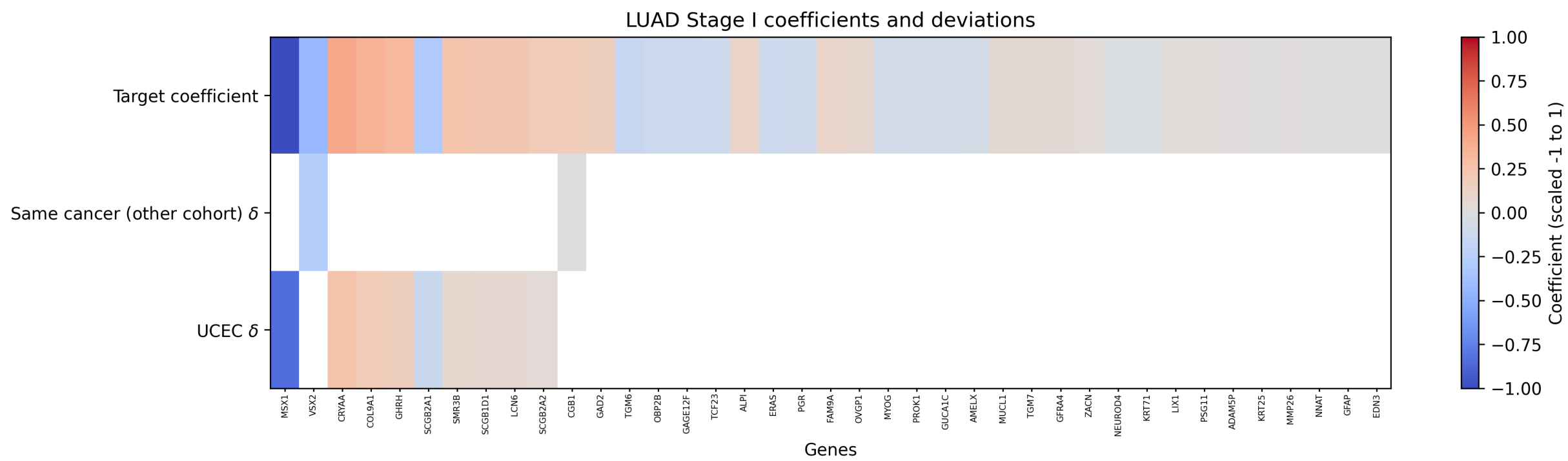

Supplementary Figure 4: Representative TransSurv coefficient–deviation heatmap for the LUAD stage I target cohort.

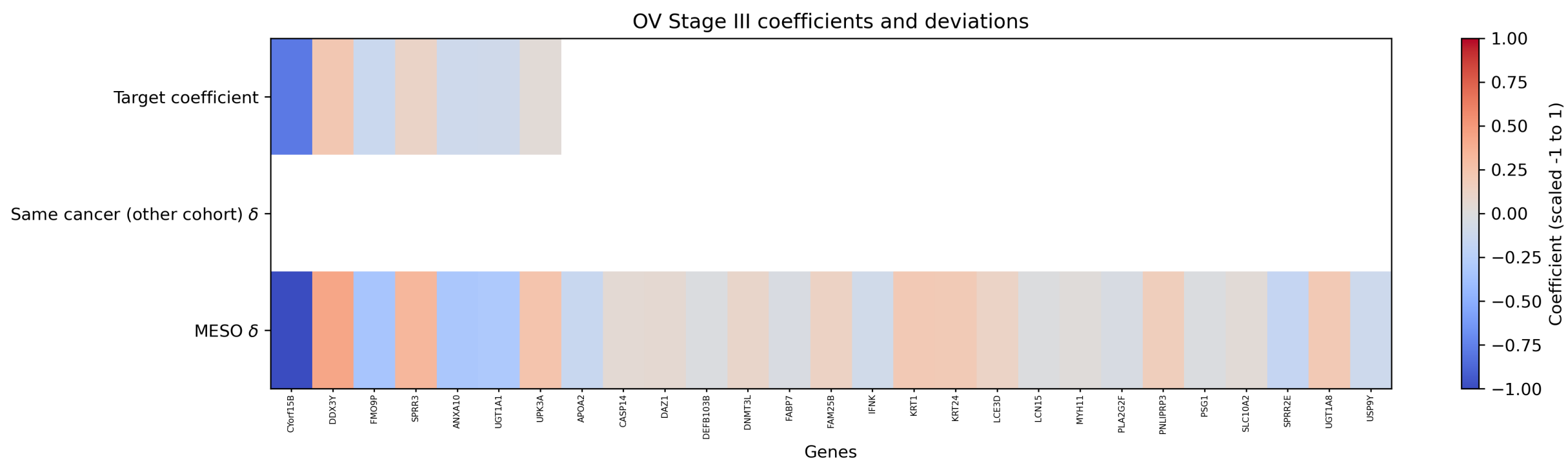

Supplementary Figure 5: Representative TransSurv coefficient–deviation heatmap for the OV stage IIIC target cohort.

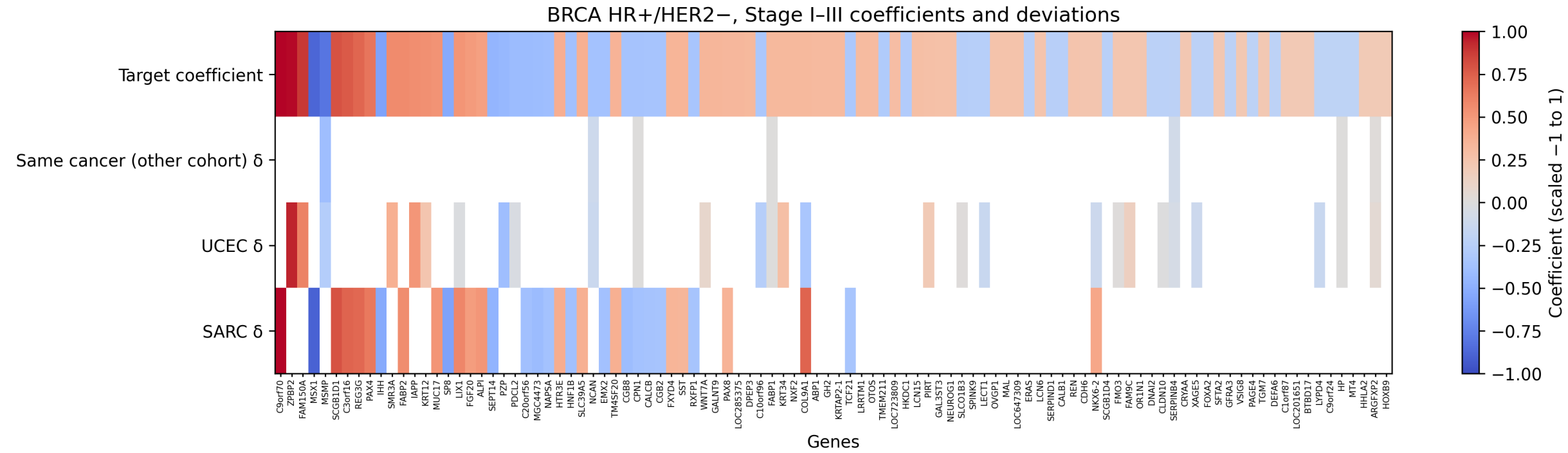

Supplementary Figure 6: Representative TransSurv coefficient–deviation heatmap for the BRCA HR+/HER2−, stage I–III target cohort.

2.4.3 FUSCC TNBC cohort

The figure below summarizes the fitted transfer structure for the FUSCC TNBC target cohort. Because the auxiliary cohorts come from independent TNBC datasets, this heatmap provides a descriptive view of how much of the dominant target signal is shared across cohorts and where cohort-specific adjustments remain.

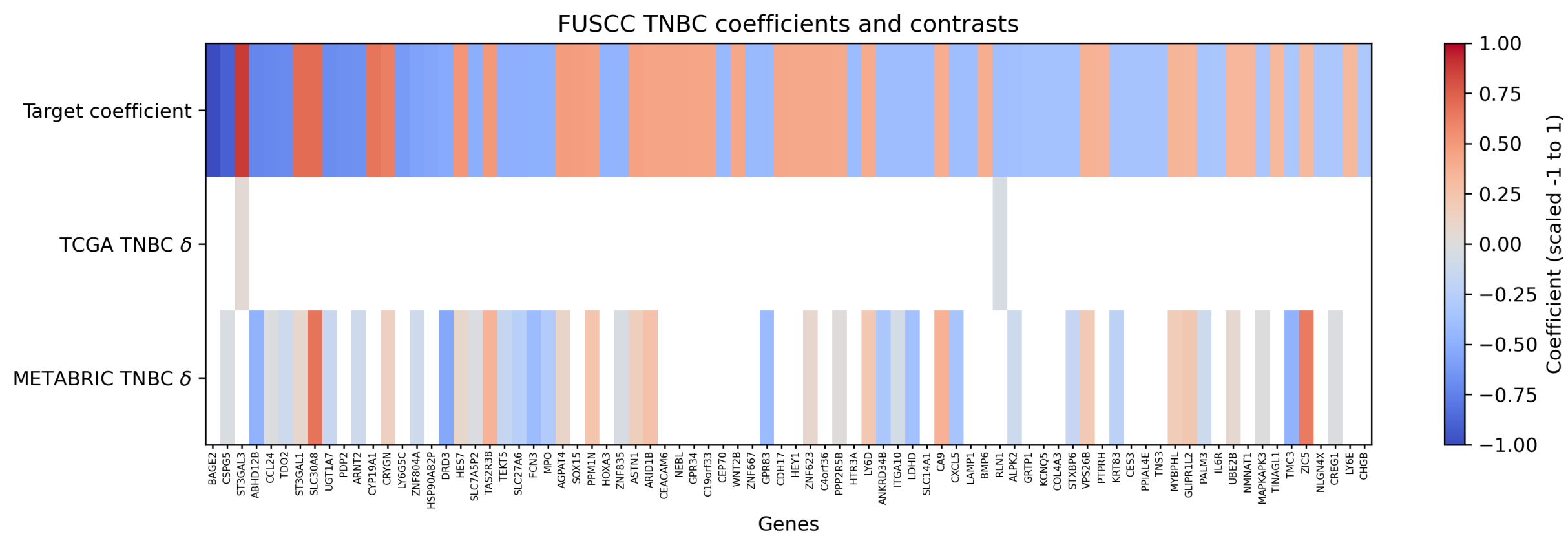

Supplementary Figure 7: Representative TransSurv coefficient–deviation heatmap for the FUSCC TNBC target cohort. Columns correspond to the 100 genes with the largest absolute target-cohort coefficients in the displayed split. The top row shows estimated target-cohort gene-expression effects, and subsequent rows show auxiliary-specific deviation terms for the retained TCGA TNBC and METABRIC TNBC cohorts relative to the target. Colors indicate signed coefficients after within-figure rescaling to $[-1, 1]$, and white indicates coefficients estimated as zero. The TCGA deviation remains highly sparse, whereas the METABRIC deviation is broader and less sparse, consistent with stronger correction under cross-platform transfer.

2.5 Clinical-covariates-only baselines

[E1] To quantify the added value of gene expression beyond routine clinical information, we fit a Cox model using only each application's prespecified baseline clinical covariates—age, sex, treatment arm, and the binary PDL1FL signature in JAVELIN Renal 101; age and pathologic stage in the

TCGA targets, with menopausal status added for BRCA and age only for OV; and age and stage in FUSCC TNBC—on the same 20 target train–test splits used throughout, evaluating in held-out target patients (Supplementary Table 8). The baseline reaches a C-index of 0.583 ± 0.030 in JAVELIN Renal 101 (versus 0.652 for TransSurv) and 0.673 ± 0.064 in FUSCC TNBC (versus 0.727). Across the five TCGA targets, TransSurv exceeds the clinical-only baseline in every cohort, with C-index margins of approximately 0.04 to 0.08 in BRCA, CESC, KIRC, and OV and a smaller margin in the stage I LUAD cohort (0.615 versus 0.600), where TransSurv also attains the higher AUC at both horizons.

Supplementary Table 8: Clinical-covariates-only baseline performance in the three applications (20 replications; mean ± SD). AUC horizons are 3 and 6 years for JAVELIN Renal 101 and the cohort-specific horizons of Supplementary Table 1 otherwise.

Target cohort	C-index	AUC@t_1	AUC@t_2
JAVELIN Renal 101	0.583 ± 0.030	0.617 ± 0.059	0.588 ± 0.050
FUSCC TNBC	0.673 ± 0.064	0.764 ± 0.070	0.715 ± 0.122
BRCA HR+/HER2−, Stage I–III	0.707 ± 0.064	0.748 ± 0.057	0.691 ± 0.117
CESC Stage IIB–IVA	0.636 ± 0.073	0.675 ± 0.103	0.704 ± 0.103
KIRC Stage I–III	0.661 ± 0.105	0.600 ± 0.120	0.675 ± 0.128
LUAD Stage I	0.600 ± 0.025	0.612 ± 0.048	0.694 ± 0.071
OV Stage IIIC	0.554 ± 0.050	0.652 ± 0.066	0.555 ± 0.090

3 TCGA cohort compatibility summaries

To complement the coefficient–deviation heatmaps, this section summarizes cross-cancer compatibility patterns for each TCGA target cohort using replication-averaged fitted contrast magnitudes together with retention frequencies. These plots are descriptive rather than inferential: they show which auxiliary cohorts were repeatedly close to the target under the fitted model and help explain the borrowing patterns discussed in Results Section 2.3.

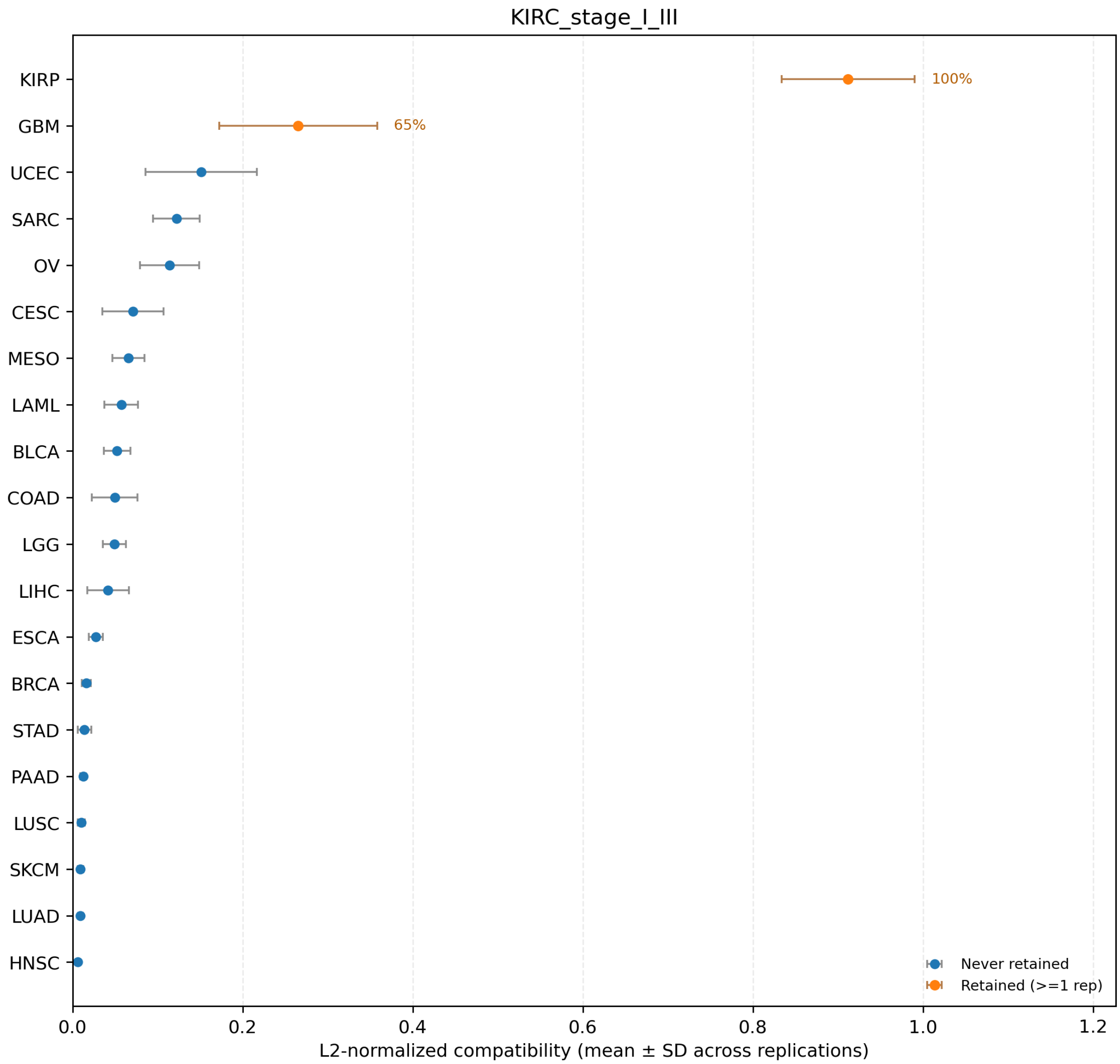

Supplementary Figure 8: Replication-averaged auxiliary-cohort compatibility profile for the KIRC stage I–III target cohort. Orange points denote cohorts retained in at least one replication; blue points denote cohorts never retained. Error bars show mean ± SD across replications.

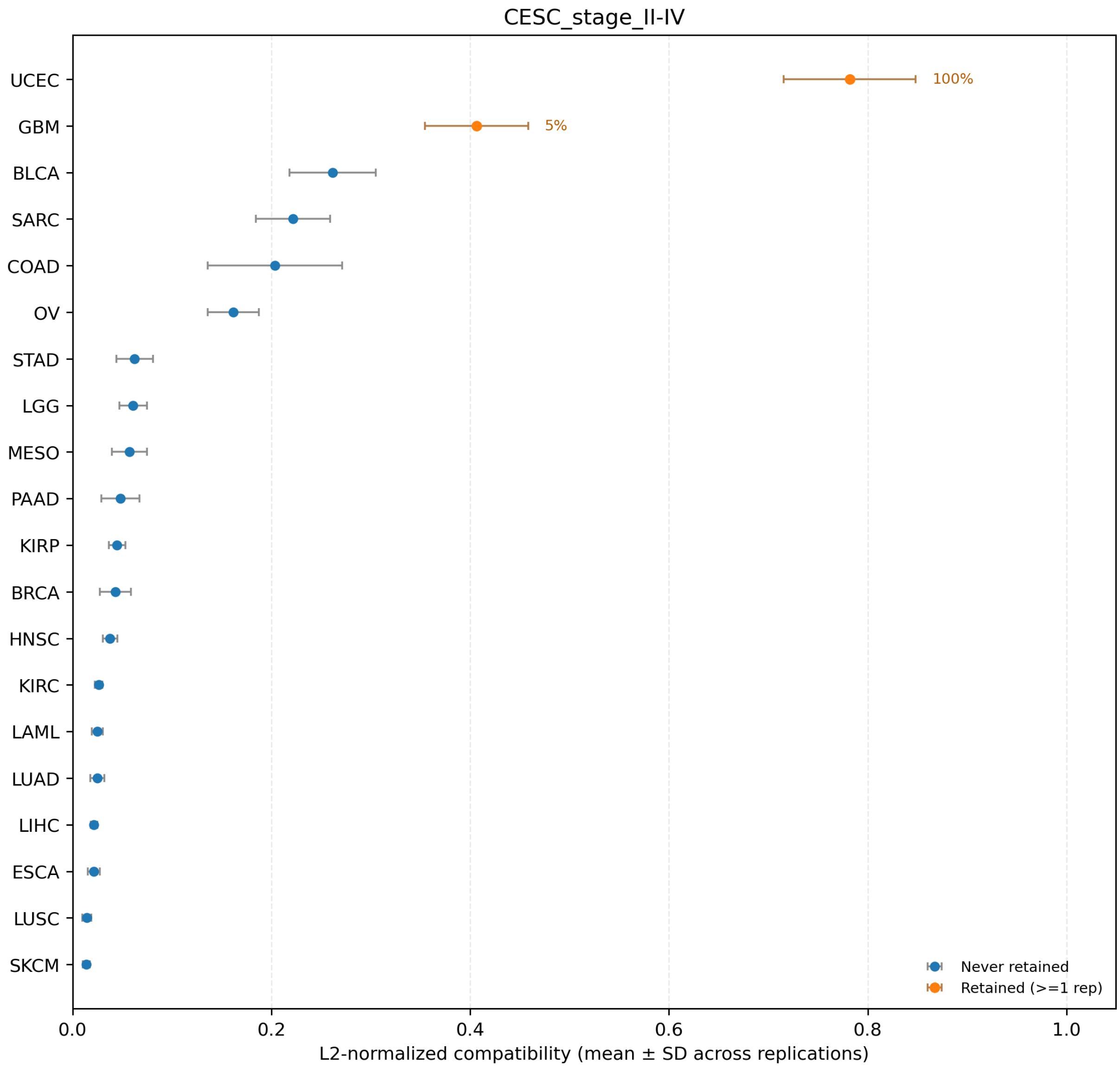

Supplementary Figure 9: Replication-averaged auxiliary-cohort compatibility profile for the CESC stage IIB–IVA target cohort. Orange points denote cohorts retained in at least one replication; blue points denote cohorts never retained. Error bars show mean $\pm$ SD across replications.

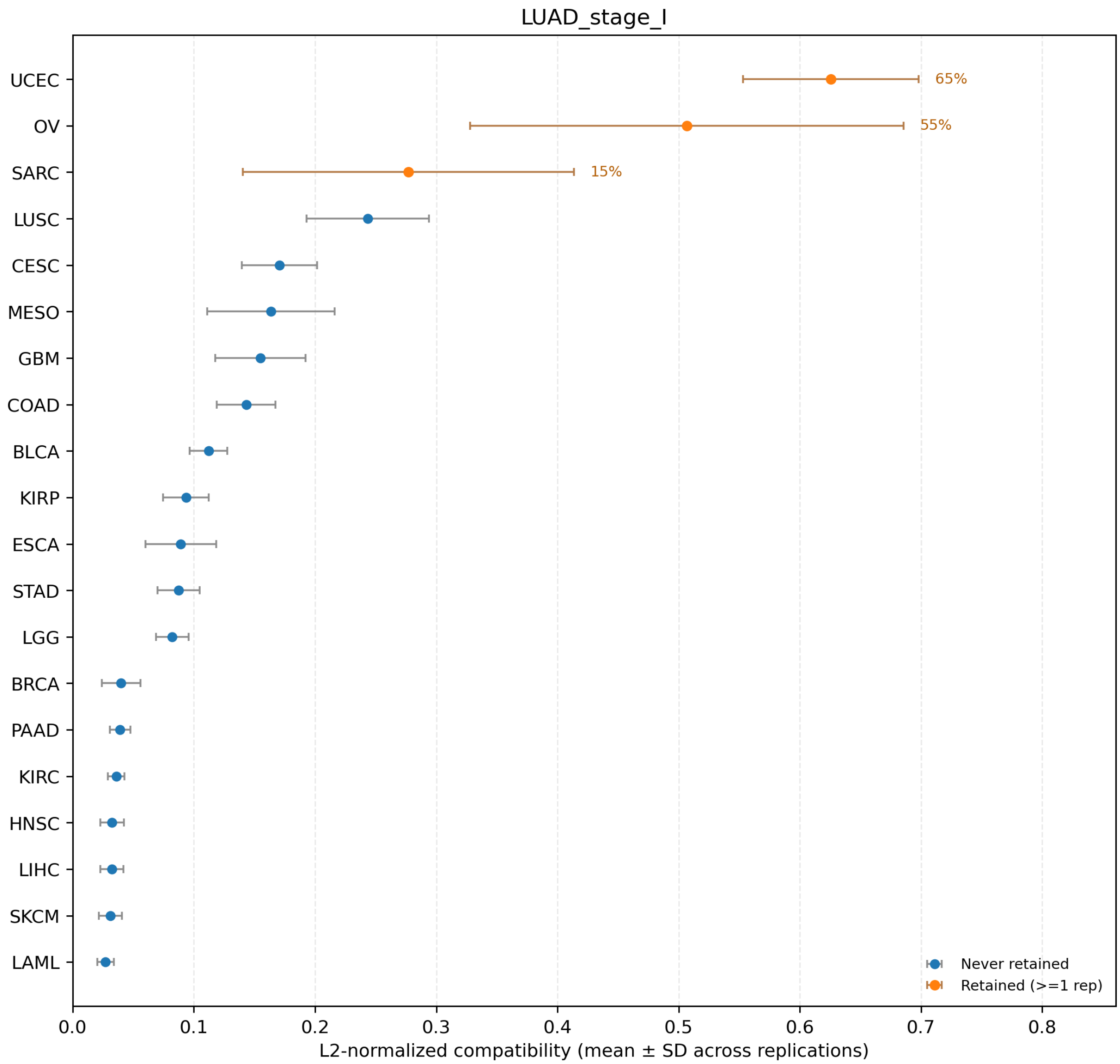

Supplementary Figure 10: Replication-averaged auxiliary-cohort compatibility profile for the LUAD stage I target cohort. Orange points denote cohorts retained in at least one replication; blue points denote cohorts never retained. Error bars show mean $\pm$ SD across replications.

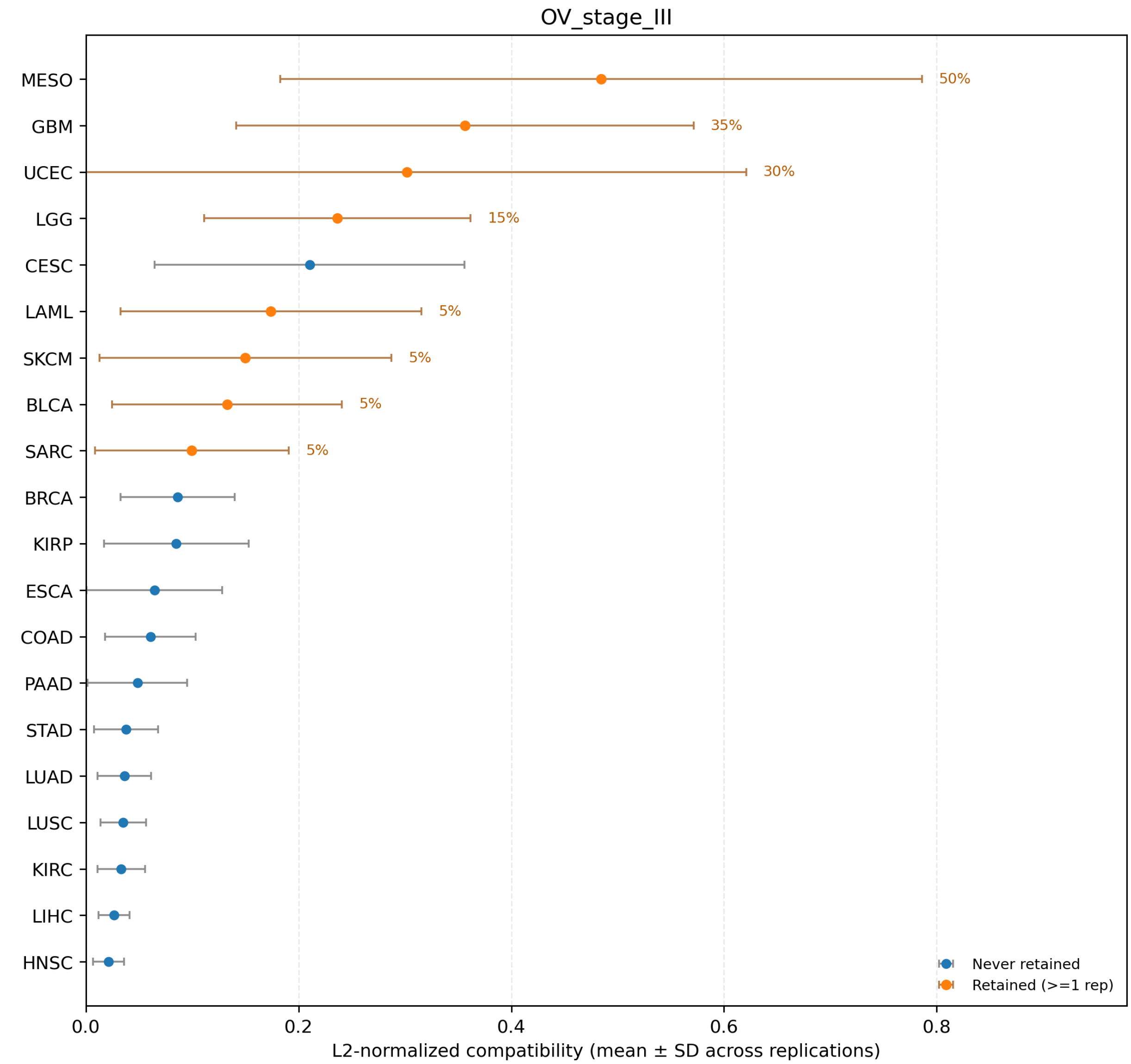

Supplementary Figure 11: Replication-averaged auxiliary-cohort compatibility profile for the OV stage IIIC target cohort. Orange points denote cohorts retained in at least one replication; blue points denote cohorts never retained. Error bars show mean $\pm$ SD across replications.

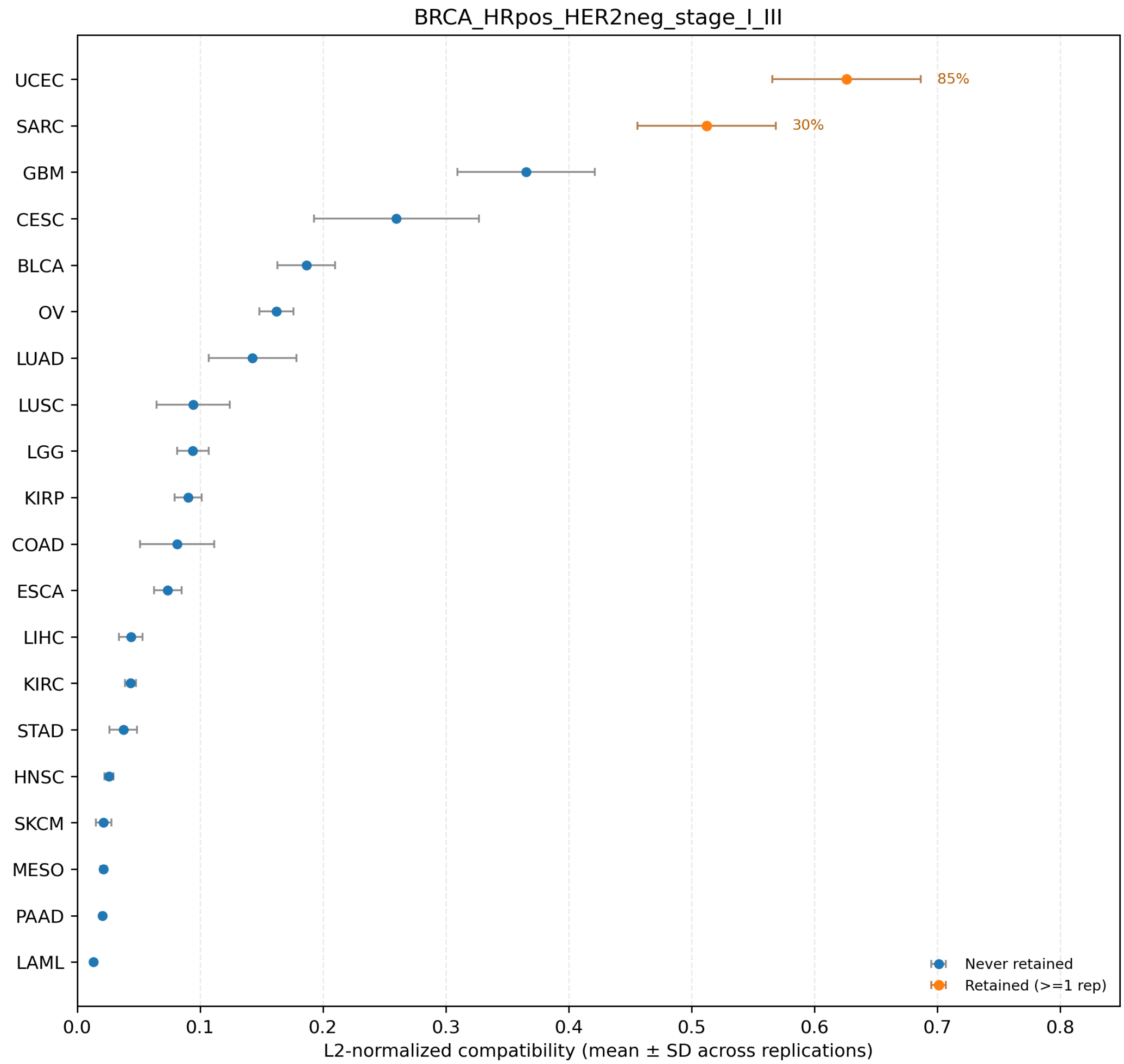

Supplementary Figure 12: Replication-averaged auxiliary-cohort compatibility profile for the BRCA HR+/HER2− stage I–III target cohort. Orange points denote cohorts retained in at least one replication; blue points denote cohorts never retained. Error bars show mean ± SD across replications.

4 Sensitivity analyses for the real-data applications

This section examines the robustness of the real-data results to three analysis choices: the normalization transform, the imputation strategy, and the gene-set restriction imposed on the target-only comparator. Each analysis reuses the corresponding application's train–test splits, tuning protocol, and held-out evaluation exactly as in the primary analyses, so that observed differences are attributable to the single choice being varied.

4.1 Sensitivity to the preprocessing transform

[E2] We repeated the JAVELIN Renal 101 and FUSCC TNBC analyses with feature-wise within-cohort z-score normalization in place of the rank-based inverse-normal transformation (INT), holding the splits, tuning protocols, and all other settings fixed. These are the two applications whose cohorts span assay platforms or projects, where the two transforms differ most; the TCGA cohorts share a single batch-corrected expression matrix, so the two transforms nearly coincide there, and the two settings examined here are therefore the conservative stress tests. Supplementary Table 9 reports the paired change in held-out C-index, $\Delta = C(\text{z-score}) - C(\text{INT})$, for each method. No refit model improves under z-scoring (MetaSurv and the $\ell_{2,1}$ comparator were not rerun). In JAVELIN, the clinical, target-only, and pooling fits are essentially unchanged ($|\Delta| \leq 0.002$) while TransSurv's borrowing gain shrinks ($\Delta = -0.012$), and TransSurv remains ahead of the refit comparators; in the cross-platform FUSCC analysis, every refit model drops by 0.021–0.034, and TransSurv still leads them under both preprocessings. This is the behavior expected if within-cohort INT removes cross-cohort differences in the marginal distribution of each gene by construction, confining residual covariate shift to the dependence structure among genes.

Supplementary Table 9: Change in held-out C-index when within-cohort z-score normalization replaces the rank-based inverse-normal transformation ($\Delta = C(\text{z-score}) - C(\text{INT})$; 20 replications, paired on identical splits).

Application	Method	ΔC-index (z-score − INT)
JAVELIN Renal 101	Clinical baseline	0.000
	Target-only	−0.001
	Naive pooling	−0.002
	TransSurv	−0.012
FUSCC TNBC	Target-only	−0.021
	Naive pooling	−0.034
	TransSurv	−0.025

4.2 Missingness exposure and imputation sensitivity

[E3] Missingness plays a limited role in the three applications, and only the TCGA analyses involve imputation. In the raw TCGA pan-cancer expression matrix (20,531 genes × 11,069 samples), 1.75% of gene–sample entries are missing; missing entries are imputed within cancer type by the gene median, never across cancer types, on the shared matrix before the stage-restricted cohorts are defined. The JAVELIN Renal 101 target expression matrix is complete (0 of 22,955 genes with missing values), and the FUSCC pipeline drops the 1,201 gene columns containing missing values rather than imputing them.

To assess sensitivity to the imputation choice, we repeated the complete five-target TCGA analysis with within-cancer-type k-nearest-neighbor imputation ($k = 10$) in place of the median, holding the seeds, splits, and all tuning parameters fixed, and compared paired per-split performance (Supplementary Table 10). Switching from median to k-nearest-neighbor imputation changes the mean TransSurv C-index by at most 0.003 in every target. Target-side changes appear only in the two targets whose own design matrices contain missing entries (LUAD and OV). The ordering of the refit models is unchanged everywhere, so the reported conclusions are insensitive to the imputation strategy.

Supplementary Table 10: Change in held-out C-index when within-cancer-type k-nearest-neighbor imputation ($k = 10$) replaces within-cancer-type median imputation in the TCGA analyses (ΔC = KNN − median, averaged over the 20 paired splits). Entries of 0.000* are exactly zero: those target designs contain no missing entries, so the target-only fits are identical under either imputer.

Target cohort	ΔC, TransSurv	ΔC, target-only
BRCA HR+/HER2−, Stage I–III	0.000	0.000*
CESC Stage IIB–IVA	+0.003	0.000*
KIRC Stage I–III	+0.001	0.000*
LUAD Stage I	+0.001	−0.021
OV Stage IIIC	+0.001	−0.003

4.3 Sensitivity of the target-only comparator to the gene-set restriction

[E9] In the JAVELIN Renal 101 and FUSCC TNBC applications, all borrowing methods operate on the gene set common to the target and candidate auxiliary cohorts (18,166 and 16,618 genes, respectively), whereas a target-only model could in principle exploit every gene measured in the target cohort. To verify that this restriction does not handicap the target-only comparator, we refit it on all post-quality-control target-cohort genes (22,955 in JAVELIN; 55,656 in FUSCC) under the identical resampling and tuning protocol (Supplementary Table 11). The full-gene refit changes the held-out C-index by −0.001 in JAVELIN and +0.016 in FUSCC, and TransSurv retains the highest held-out discrimination in both applications; the TCGA cohorts all draw on the same 20,531-gene panel, so the intersection removes nothing and no refit is required there. The reported conclusions are therefore insensitive to restricting the target-only comparator to the intersected gene set.

Supplementary Table 11: Change in held-out C-index of the target-only comparator when it is refit on all post-quality-control target-cohort genes in place of the intersected gene set (ΔC = full − intersected, averaged over the 20 paired splits; both arms use the same resampling and tuning protocol within each application).

Application	Genes, full vs. intersected	ΔC, target-only
JAVELIN Renal 101	22,955 vs. 18,166	−0.001
FUSCC TNBC	55,656 vs. 16,618	+0.016

5 Simulations under controlled heterogeneity

These simulations are designed to clarify the two mechanisms underlying TransSurv under controlled heterogeneity. First, we study the estimation benefit of the joint target-centered Cox fit when auxiliary cohorts are compatible with the target. Second, we examine whether fitted contrast magnitudes can separate compatible from incompatible auxiliary cohorts in finite samples. Together, these experiments support the target-centered transfer logic developed in the technical and theoretical sections.

5.1 Target-centered Cox model reduces estimation error under compatible transfer

To study the estimation behavior of the target-centered Cox component of TransSurv under controlled heterogeneity, we generated one target cohort and K candidate auxiliary cohorts from a

Cox proportional hazards model with log-risk $\eta = x^\top \omega$. For each cohort, covariates were drawn as $x \sim N(0, \Sigma_k)$ with Toeplitz covariance $\Sigma_k = (\rho_k^{|j-j'|})_{j,j'=1}^p$. The target cohort used baseline correlation $\rho_0 = 0.3$, whereas auxiliary cohorts used $\rho_k \sim \mathrm{Unif}(\rho_0 - \rho_\pm, \rho_0 + \rho_\pm)$, so that $\rho_\pm$ controls the magnitude of covariate shift across cohorts. Event times were generated from an exponential-baseline Cox model with baseline hazard 0.1 and independent exponential censoring hazard 0.08. To reflect the preprocessing used in the real-data analyses, we additionally imposed MCAR missingness at rate MCAR $\in \{0, 0.1, 0.2\}$ and then performed within-cohort median imputation before fitting.

We fixed $p = 200$ and generated a sparse target coefficient vector $\omega^\star$ with support size $s = 12$. Auxiliary-cohort coefficient were generated as $\omega^{(k)} = \omega^\star + \delta^{(k)}$, where each contrast $\delta^{(k)}$ is sparse, with support size $s_\delta = \lfloor s/2 \rfloor$ and moderate nonzero magnitudes. This places the experiment in the compatible-transfer regime studied in Theorem 1: heterogeneity enters through sparse coefficient contrasts together with controlled covariance shift. We compared a target-only ℓ_1-penalized Cox model with the joint target-centered Cox fit, using penalty levels $\lambda_\omega \asymp \sqrt{\log(p)/(n_0 + n_{\mathcal{A}})}$ and $\lambda_{\delta,k} \asymp \sqrt{\log(p)/n_k}$ as suggested by the theory. This experiment is intended to isolate the estimation gain once auxiliary cohorts are retained; the protection against harmful transfer is examined separately in Section 5.2.

Figure 13 reports the improvement in estimation mean-squared error,

$$\Delta\mathrm{MSE} = \|\widehat{\omega}_{\mathrm{target}} - \omega^\star\|_2^2 - \|\widehat{\omega}_{\mathrm{TL}} - \omega^\star\|_2^2,$$

as a function of the target sample size n_0, with $K = 5$ auxiliary cohorts and $n_k = n_0$ for each auxiliary cohort in this grid. Across all panels, ΔMSE is positive, showing that target-centered borrowing through the joint target-centered Cox fit improves estimation accuracy even under moderate covariate shift and missingness. The pattern across n_0 is consistent with Theorem 1: once finite-sample remainder terms become less dominant, the leading estimation term is governed by the effective sample size $n_0 + n_{\mathcal{A}}$ rather than n_0 alone. Increasing $\rho_\pm$ weakens the gain by increasing design heterogeneity, and increasing MCAR weakens the gain by reducing the effective information in the covariates after imputation. Neither effect removes the advantage of compatible transfer in this regime. Overall, this experiment shows that, when auxiliary cohorts are compatible with the target up to sparse deviations, the joint target-centered Cox fit delivers the expected reduction in estimation error predicted by the theory.

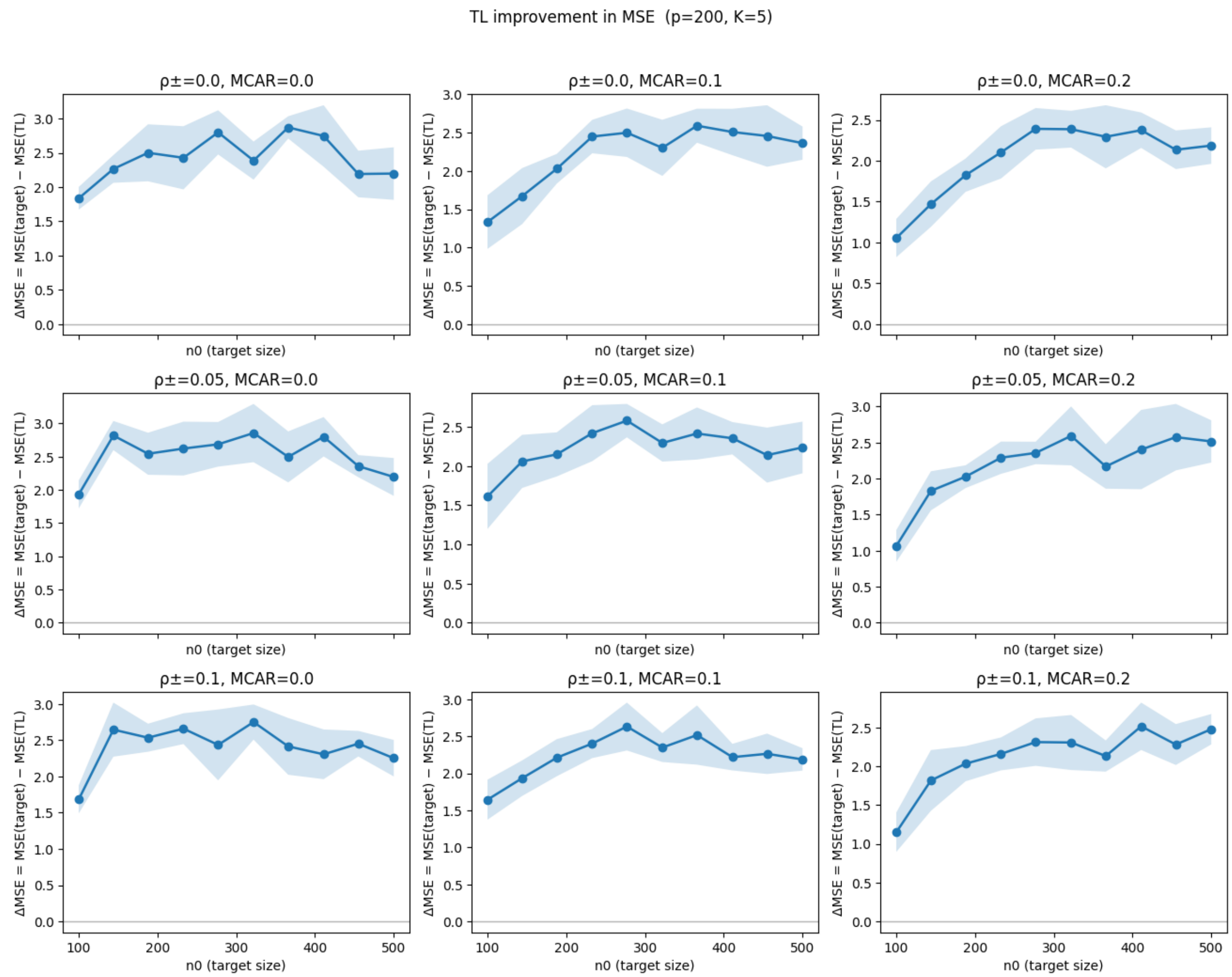

Supplementary Figure 13: Target-centered Cox model reduces estimation error under compatible transfer. Positive values of ΔMSE indicate lower estimation error than target-only fitting, with gains attenuated but not eliminated by stronger covariate shift ($\rho_{\pm}$) and missingness (MCAR).

5.2 Contrast-based screening separates compatible and incompatible auxiliary cohorts

To examine whether fitted contrast magnitudes separate compatible from incompatible auxiliary cohorts in finite samples, we generated data with a mixture of transferable and non-transferable auxiliary cohorts. Transferable auxiliary cohorts shared the target coefficient vector, whereas a fixed subset of non-transferable cohorts was contaminated by an added sparse contrast on the active set, producing materially different regression effects. We varied the target sample size n_0, the per-cohort sample size n_k, and a severity parameter controlling the magnitude of the contamination. We then fit a pilot model and summarized the resulting fitted deviations $\widehat{\delta}^{(k)}$.

For this diagnostic experiment, we used a simple elbow rule applied to the fitted deviation magnitudes $\|\widehat{\delta}^{(k)}\|_\infty$ to visualize separation between compatible and incompatible auxiliary cohorts. This is best viewed as a finite-sample screening diagnostic rather than as an exact replica of the deployed retained-set selection rule in the main analysis. The purpose here is not to optimize the

final retained set, but to show that fitted contrasts become more informative about auxiliary-cohort compatibility as signal-to-noise ratio improves.

Figure 14 shows that screening performance improves in the expected directions. Increasing n_k sharpens estimation of the cohort-specific contrasts and therefore improves separation between near-zero deviations from compatible auxiliary cohorts and larger deviations from contaminated cohorts. Increasing contamination severity widens the same gap, which raises specificity and, when the elbow cutoff is not overly conservative, also improves sensitivity. The dependence on n_0 is consistent with Theorem 2: when the target cohort is small, the pilot fit is noisier and the fitted contrasts are less stable; as n_0 increases, the target-centered component stabilizes and the separation becomes more structurally detectable. Overall, this experiment provides finite-sample support for the main screening principle of TransSurv: when auxiliary–target contrasts are sparse and sufficiently separated, fitted deviation magnitudes can help identify auxiliary cohorts that are useful for borrowing while excluding cohorts that are likely to induce harmful transfer.

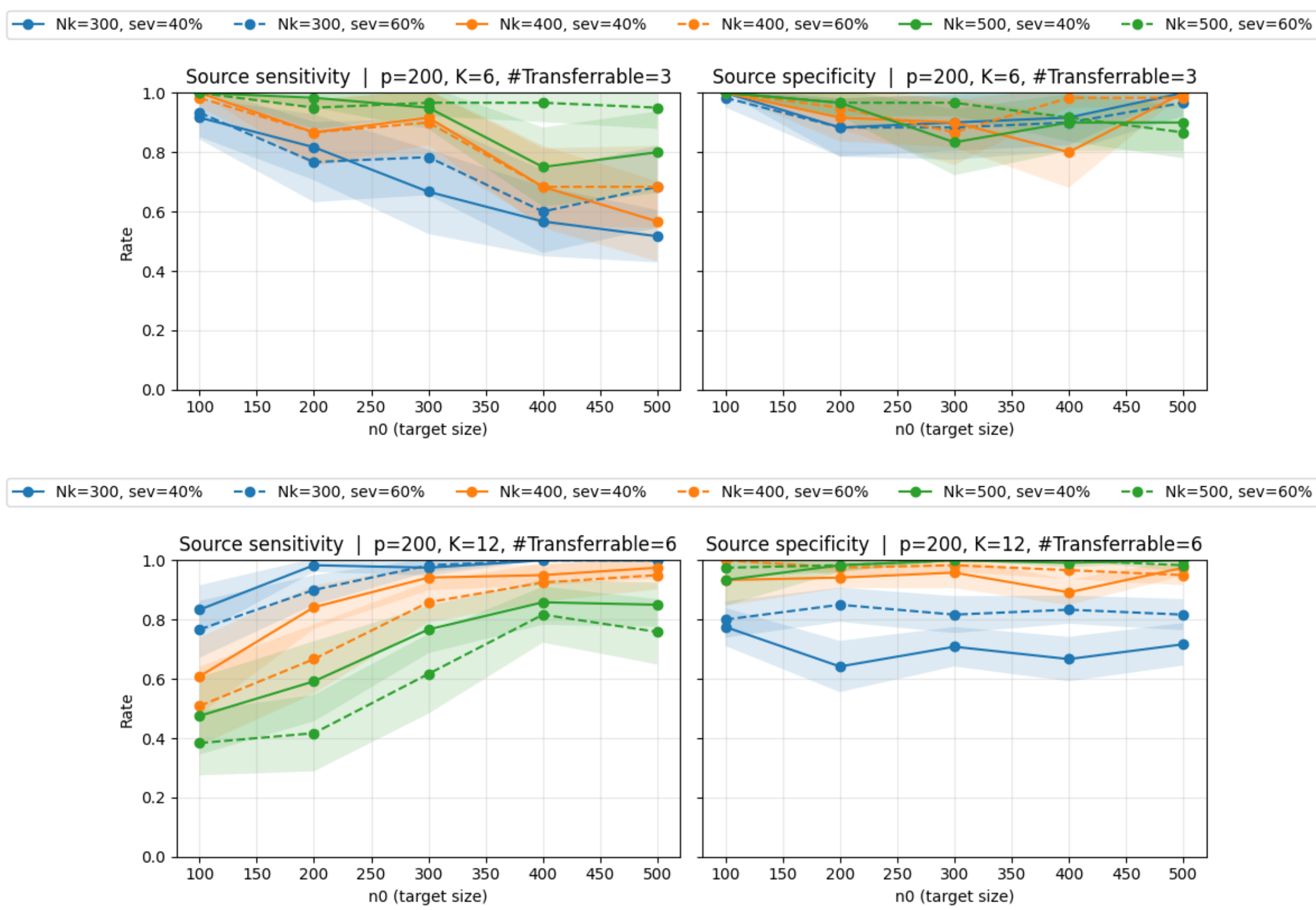

Supplementary Figure 14: Contrast-based screening more reliably separates compatible from incompatible auxiliary cohorts as target and auxiliary sample sizes increase and contamination becomes stronger. Panels show sensitivity and specificity of the fitted-contrast diagnostic across simulation settings.

[E5] In the deployed workflow, candidates are ranked by D_k and the retained count is selected on the target training split by increasing it along the ranking while the training-side selection criterion improves (Section 1, Proposition 1); the separation documented here is the quantity on

which that rule operates.

5.3 Companion simulations at transcriptome dimensionality ($p = 20{,}000$)

[E4] We repeated the estimation design of Section 5.1 and the screening design of Section 5.2 at $p = 20{,}000$, the scale of the real-data applications, keeping the same sample sizes and support sizes and recalibrating the penalty-rate constants per dimension on separate calibration replicates. Both findings are preserved (Figures 15 and 16): borrowing reduces estimation error in every arm, again attenuated but not eliminated by design-covariance heterogeneity and missingness, and the contrast-based screen retains high sensitivity and specificity throughout. This is what the theory predicts, since the rates of Section 6 depend on the dimension only through $\log p$.

A further arm isolates covariate shift by setting all coefficient contrasts to zero while varying the cohort covariances $\rho_{\pm}$: borrowing improves held-out discrimination at every target size, with no negative transfer, in line with the role of $C_{\Sigma}(\mathcal{A})$ in Theorem 1. As at $p = 200$, these experiments demonstrate consistency behavior under controlled conditions; they are not comparisons with competing methods, and we do not expect the consistency regime to be reached in finite real-data cohorts.

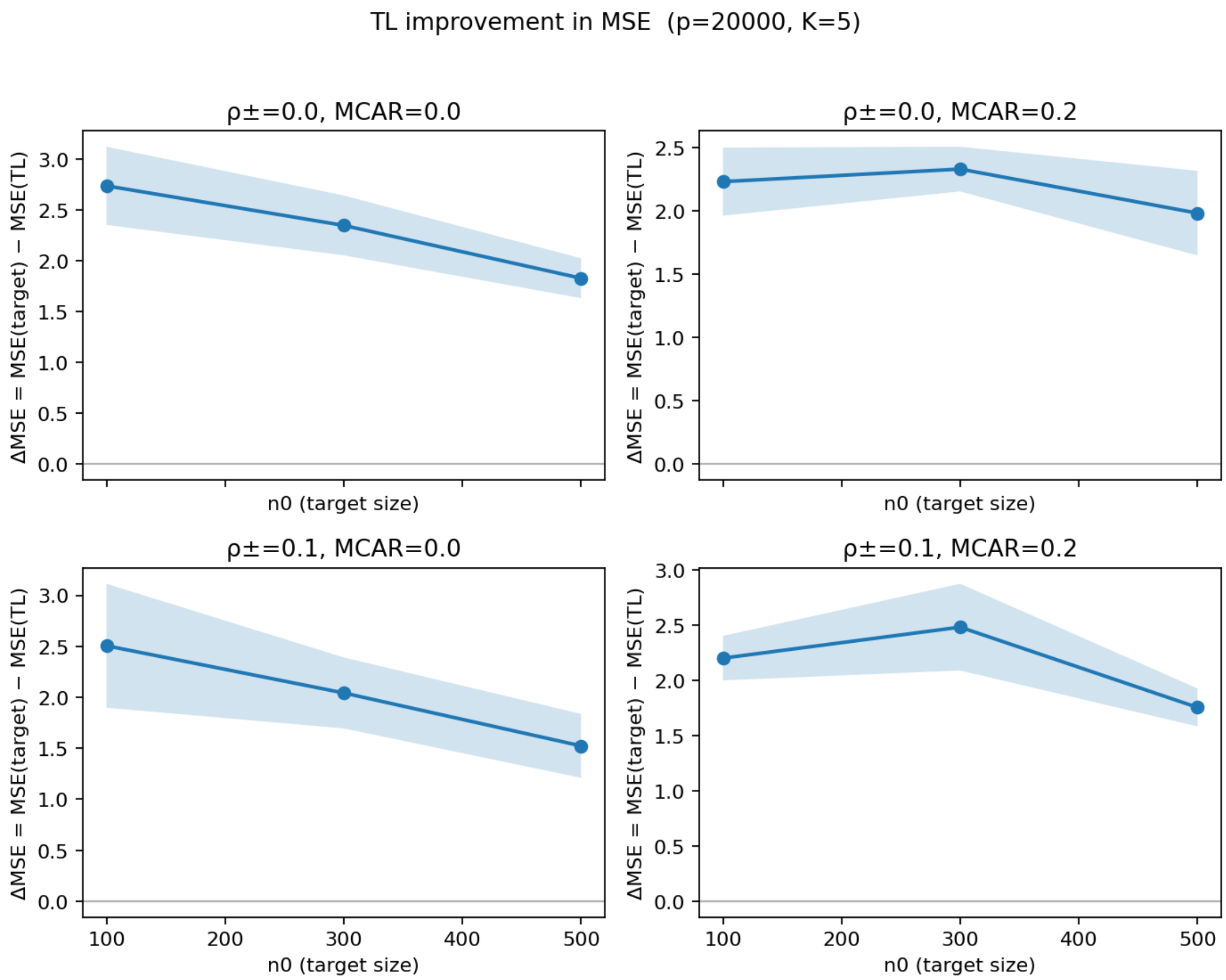

Supplementary Figure 15: Estimation simulations at $p = 20{,}000$ ($K = 5$): improvement in estimation error from target-centered borrowing (ΔMSE as in Figure 13; positive values favor transfer) by target sample size n_0, design-covariance heterogeneity $\rho_{\pm}$, and missingness (MCAR). Ribbons show mean $\pm\, 1.96{\cdot}\mathrm{SE}$ across replicates.

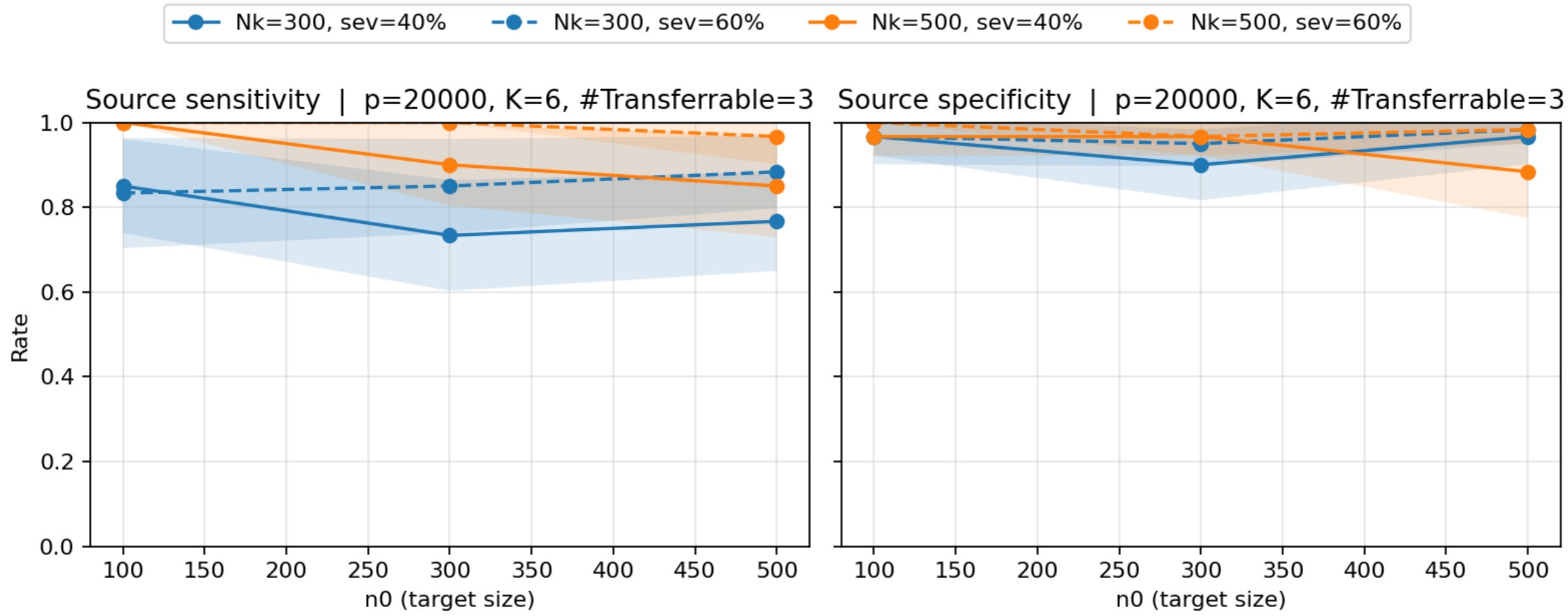

Supplementary Figure 16: Screening simulations at $p = 20{,}000$ ($K = 6$ candidate auxiliary cohorts, three transferable): sensitivity (left) and specificity (right) of the contrast-based screen by target size n_0, auxiliary size N_k, and contamination severity.

6 Statistical Guarantees for TransSurv

Overview and proof roadmap

In this section we formalize the target-centered transfer model underlying TransSurv, introduce the technical assumptions, and establish guarantees for the target-centered Cox estimator and its practical auxiliary-screening rule. First, Theorem 1 gives oracle-rate bounds for the joint transfer-learning Cox estimator when the transferable set is known, allowing auxiliary–target coefficient differences of radius $h = h_{n_0,p} \geq 0$ and covariate shift measured by the heterogeneity factor $C_\Sigma(\mathcal{A})$. The result shows that the target coefficient enjoys the leading high-dimensional rate $s \log p/(n_0 + n_{\mathcal{A}})$, up to additional terms reflecting residual auxiliary–target mismatch.

Theorem 2 then shows that the practical contrast-based screening rule consistently recovers a nonempty oracle transferable subset under the stated separation and event-information conditions. Together, these results justify the main TransSurv design principle: compatible auxiliary cohorts can be screened and then jointly incorporated to improve target-cohort estimation while controlling harmful transfer.

Throughout, the analysis permits study-specific baseline hazards and heterogeneous covariate distributions across studies under Assumption 4. All guarantees are derived in the high-dimensional regime with $s \log p/(n_0 + n_{\mathcal{A}}) = o(1)$ and bounded or sub-Gaussian covariates.

6.1 Model setup, notation, and assumptions for TransSurv

We observe a *target* cohort $k = 0$ with i.i.d. tuples $(T_i^{(0)}, \Delta_i^{(0)}, X_i^{(0)})$, where T is the observed time, $\Delta \in \{0, 1\}$ indicates event, and $X \in \mathbb{R}^p$ are covariates. For each auxiliary cohort $k \in [K] := \{1, \ldots, K\}$ we observe $(T_i^{(k)}, \Delta_i^{(k)}, X_i^{(k)})$, $i = 1, \ldots, n_k$. Denote n_0 the target size and n_k the k-th auxiliary size; $n_{\mathcal{S}} = \sum_{k \in \mathcal{S}} n_k$ for any index set $\mathcal{S}$. The true target coefficient is $\omega^{(0)*} \in \mathbb{R}^p$ with sparsity $s = \|\omega^{(0)*}\|_0$, and similarly each auxiliary cohort has coefficient $\omega^{(k)} \in \mathbb{R}^p$. We define the (sparse) parameter shift $\delta^{(k)} := \omega^{(k)} - \omega^{(0)*}$. Throughout, p may exceed n_0 and $\sum_k n_k$. For cohort

k, let $\ell_k(\omega)$ be the *average* negative partial loglikelihood for Cox (Breslow ties):

$$\ell_k(\omega) = \frac{1}{n_k}\sum_{i=1}^{n_k}\Big\{ -\Delta_i^{(k)} X_i^{(k)\top}\omega + \Delta_i^{(k)} \log\Big(\sum_{j:T_j^{(k)} \ge T_i^{(k)}} e^{X_j^{(k)\top}\omega}\Big)\Big\}.$$

Here the risk-set sum $\sum_{j:T_j^{(k)} \ge T_i^{(k)}}$ is computed using only observations from study k. The counting process formulation underlying the Cox partial likelihood is developed in Andersen and Gill [1982] and the monograph by Fleming and Harrington [1991]. Because each ℓ_k is defined using study k's observations alone, the study-specific baseline hazard $\lambda_0^{(k)}(t)$ cancels in the partial likelihood ratio at each event time. Consequently, the theory accommodates heterogeneous baseline hazards across studies without modification; this is the same principle underlying stratified Cox models [Andersen and Gill, 1982, Fleming and Harrington, 1991]. Let $U_k(\omega) = \nabla \ell_k(\omega)$ and $H_k(\omega) = \nabla^2 \ell_k(\omega)$ denote score and local information. For an index set $\mathcal{C} \subseteq [K]$, define the pooled objective and average information

$$\ell_{\mathcal{C}}(\omega) = \frac{1}{n_0 + n_{\mathcal{C}}} \sum_{k\in\{0\}\cup\mathcal{C}} n_k\,\ell_k(\omega), \qquad \bar{H}_{\mathcal{C}}(\omega) = \frac{1}{n_0 + n_{\mathcal{C}}} \sum_{k\in\{0\}\cup\mathcal{C}} n_k\,H_k(\omega).$$

For a radius $h > 0$ and $q \in [0, 1]$, define the oracle transferable set

$$\mathcal{A}_q(h) := \big\{k \in [K] :\ \|\delta^{(k)}\|_q \le h\big\}.$$

Below we write $\mathcal{A} \equiv \mathcal{A}_1(h)$ unless stated.

Covariate shift & sparse compatibility. We allow heterogeneous covariate distributions across studies. Let $\mathcal{N}(\omega, r) := \{b \in \mathbb{R}^p :\ \|b - \omega\|_1 \le r\}$, and define restricted eigenvalues for target and pooled designs:

$$\phi_{\min}^{(0)}(s) := \inf_{v\neq 0,\ \|v\|_0 \le s} \frac{v^\top H_0(\omega^*) v}{\|v\|_2^2}, \qquad \phi_{\min}^{(\mathcal{A})}(s) := \inf_{v\neq 0,\ \|v\|_0 \le s} \frac{v^\top \bar{H}_{\mathcal{A}}(\omega^*) v}{\|v\|_2^2}.$$

We also quantify covariance heterogeneity via

$$C_\Sigma(\mathcal{A}) \ := \ 1 + \max_{1\le j\le p}\ \max_{k\in\mathcal{A}}\ \big\|e_j^\top \big(H_k(\omega^*) - H_0(\omega^*)\big)\,\bar{H}_{\mathcal{A}}(\omega^*)^{-1}\big\|_1.$$

Intuitively, $C_\Sigma(\mathcal{A})$ is small when auxiliary and target cohort information matrices are similar along sparse directions. All results below hold under $C_\Sigma(\mathcal{A}) = O(1)$, and we will explicitly track its effect. We pose the following assumptions on the data generation machanisms to control the data sampling, covariance shifts and coefficient contrasts.

Assumption 1 (Data, censoring, and tails)**.** *(i) (Independent sampling) Within each study, observations are i.i.d. For each study k, let $T^{(k)*}$ denote the true (latent) event time and $C^{(k)}$ the censoring time. Conditional independent censoring holds: $C^{(k)} \perp\!\!\!\perp T^{(k)*} \mid X^{(k)}$. The observed follow-up time is $T^{(k)} = \min(T^{(k)*}, C^{(k)})$ and the event indicator is $\Delta^{(k)} = \mathbf{1}\{T^{(k)*} \le C^{(k)}\}$. We assume $\inf_t P(C^{(k)} \ge t \mid X^{(k)}) \ge c_G > 0$ for some constant c_G. (ii) (Bounded covariates) $\|X^{(k)}\|_\infty \le K_X$ a.s., or sub-Gaussian tails with parameter σ_X. (iii) (Baseline) The true hazard follows a Cox model in each study with coefficient $\omega^{(k)}$ and baseline hazards bounded away from 0 and ∞ on $[0, \tau]$.*

Assumption 2 (Sparsity and transferable set)**.** *The target is s-sparse, $s\log p/(n_0+n_{\mathcal{A}})=o(1)$. For each $k\in\mathcal{A}$, $\|\delta^{(k)}\|_1\le h$ with $h\sqrt{\log p/n_0}=o(1)$. Non-informative auxiliaries need not satisfy sparsity.*

Assumption 3 (Restricted strong convexity (RSC))**.** *There exist constants $\kappa_0,\kappa_{\mathcal{A}}>0$ such that with probability $1-o(1)$,*

$$\begin{aligned} v^\top H_0(\omega)v &\ge \kappa_0\,\|v\|_2^2 && \text{for all } \omega\in\mathcal{N}(\omega^*,r_0),\ v\in\mathbb{R}^p \text{ with } \|v_{S^c}\|_1\le 3\|v_S\|_1,\\ v^\top \bar H_{\mathcal{A}}(\omega)v &\ge \kappa_{\mathcal{A}}\,\|v\|_2^2 && \text{for all } \omega\in\mathcal{N}(\omega^*,r_{\mathcal{A}}),\ v\in\mathbb{R}^p \text{ with } \|v_{S^c}\|_1\le 3\|v_S\|_1,\end{aligned}$$

where $S=\mathrm{supp}(\omega^*)$.

Assumption 4 (Covariate shift: sparse compatibility)**.** *For $k\in\mathcal{A}$, $\|H_k(\omega^*)-H_0(\omega^*)\|_\infty\le c_H$ and the heterogeneity factor $C_\Sigma(\mathcal{A})=O(1)$. This allows $H_k\ne H_0$ but rules out adversarial directions that would amplify sparse contrasts.*

Here we restate the TransSurv-side estimator and screening rule used in the theoretical analysis, with minor proof-side adjustments that simplify the arguments but do not change the practical implementation or the stated rates.

Alg 1: (**Oracle target-centered Cox estimator**) Given the oracle transferable set $\mathcal{A}\subseteq[K]$, estimate $(\omega,\{\delta^{(k)}\}_{k\in\mathcal{A}})$ by the joint convex program

$$(\hat\omega_{\mathrm{or}},\{\hat\delta^{(k)}\})\in\arg\min_{\omega,\{\delta^{(k)}\}}\Big\{\ell_{\mathcal{A}}(\omega)+\frac{1}{n_0+n_{\mathcal{A}}}\sum_{k\in\mathcal{A}}n_k\big(\ell_k(\omega+\delta^{(k)})-\ell_k(\omega)\big)+\lambda_\omega\|\omega\|_1+\sum_{k\in\mathcal{A}}\lambda_{\delta,k}\|\delta^{(k)}\|_1+\frac{\mu}{2}\Xi\Big\}, \tag{4}$$

where $\Xi=\|\omega\|_2^2+\sum_{k\in\mathcal{A}}\|\delta^{(k)}\|_2^2$ and $(\lambda_\omega,\lambda_{\delta,k})\asymp\big(\sqrt{\log p/(n_0+n_{\mathcal{A}})},\sqrt{\log p/n_k}\big)$. The additional ridge term (with $\mu\lesssim(n_0+n_{\mathcal{A}})^{-1}$) is used only to simplify strict-convexity/uniqueness arguments under covariate shift; it is asymptotically negligible. One may set $\mu=0$ in both theory and practice without affecting the stated rates, provided Assumptions 3 and 4 hold. (For $k\notin\mathcal{A}$, take $\delta^{(k)}\equiv 0$.)

Alg 2: (**Auxiliary-cohort Screening**) We analyze the same practical contrast-based screening rule as in the main text (Algorithm 1); for readers' convenience we restate it here. Given datasets $\{(T^{(k)},\Delta^{(k)},X^{(k)})\}_{k=0}^K$, penalties $\lambda_0\asymp\sqrt{\log p/n_0}$ and $\{\lambda_k\asymp\sqrt{\log p/n_k}\}_{k=1}^K$, and thresholds $c_1>0$, $t_2\in(0,1)$:

(a) *Initial fit:* solve (2) on $\{0\}\cup\{1,\dots,K\}$ to obtain $(\widehat\omega_0,\{\widehat\delta^{(k)}\}_{k=1}^K)$.

(b) *Screening:* compute $D_k=\|\widehat\delta^{(k)}\|_1$ and $I_k=n_k^{-1}\sum_{i=1}^{n_k}\Delta_i^{(k)}$. Define the transferable set $\widehat C=\{\,k:\ D_k\le c_1(\lambda_0+\lambda_k)\ \wedge\ I_k\ge t_2\,\}$

6.2 Main TransSurv Results

We state two TransSurv results. A short proof of Theorem 2 is given inline; the proof of Theorem 1 then follows using the auxiliary lemmas collected in §6.4.

Theorem 1 (Oracle rate for the target-centered Cox estimator underlying TransSurv)**.** *Under Assumptions 1–4, let $(\hat\omega_{\mathrm{or}},\{\hat\delta^{(k)}\})$ solve (4) with tuning $(\lambda_\omega,\lambda_{\delta,k})$ as above and $\mu\lesssim(n_0+n_{\mathcal{A}})^{-1}$.*

If $s\log p/(n_0+n_{\mathcal{A}}) + C_\Sigma(\mathcal{A})\, h\sqrt{\log p/n_0} = o(1)$, then with probability $1-o(1)$,

$$\|\hat{\omega}_{\rm or}-\omega^*\|_2^2 \ \vee\ \frac{1}{n_0}\sum_{i=1}^{n_0}\Big\{\ell_0(\hat{\omega}_{\rm or})-\ell_0(\omega^*)-\nabla\ell_0(\omega^*)^\top(\hat{\omega}_{\rm or}-\omega^*)\Big\} \tag{5}$$
$$\lesssim \frac{s\log p}{n_0+n_{\mathcal{A}}} + \min\left(\frac{s\log p}{n_0},\ C_\Sigma(\mathcal{A})\, h\sqrt{\frac{\log p}{n_0}},\ C_\Sigma(\mathcal{A})^2 h^2\right).$$

Theorem 2 (Selection consistency of the practical TransSurv screening rule)**.** *Assume 1–4. Let the penalties satisfy $\lambda_0 \asymp \sqrt{\log p/n_0}$ and $\lambda_k \asymp \sqrt{\log p/n_k}$. Consider the practical screening rule*

$$\widehat{C} = \big\{\, k\in[K]:\ D_k \le c_1(\lambda_0+\lambda_k)\ \wedge\ I_k \ge t_2 \,\big\}, \qquad D_k := \|\widehat{\delta}^{(k)}\|_1,\quad I_k := n_k^{-1}\sum_{i=1}^{n_k}\Delta_i^{(k)}.$$

Suppose the event–fraction gate is feasible for some nonempty oracle subset, $\inf_{k\in\mathcal{A}} I_k \ge t_2 > 0$, and the marginal separation conditions of Lemma 4 hold. Then there exist constants $0<c_-<c_+<\infty$ and a nonempty subset $\mathcal{A}_o\subseteq\mathcal{A}$ such that, for any $c_1\in(c_-,c_+)$,

$$\Pr\big(\widehat{C}=\mathcal{A}_o\big) \ \longrightarrow\ 1.$$

Consequently, the practical contrast-based screening rule recovers the same oracle transferable subset $\mathcal{A}_o$ identified by the proof-side marginal separation argument in Lemma 4, with probability $1-o(1)$.

Proof. By Lemma 4 (the score–divergence selection lemma), there exists a nonempty $\mathcal{A}_o\subseteq\mathcal{A}$ such that, with probability $1-o(1)$, $\max_{k\in\mathcal{A}_o}\widehat{R}^{(k)} < \min_{k\in\mathcal{A}^c}\widehat{R}^{(k)}$. Lemma 5 (equivalence of diagnostics) then implies the existence of constants $0<c_-<c_+<\infty$ for which, w.p. $1-o(1)$,

$$D_k \ \le\ c_-(\lambda_0+\lambda_k) \quad \text{for all } k\in\mathcal{A}_o, \qquad D_{k'} \ \ge\ c_+(\lambda_0+\lambda_{k'}) \quad \text{for all } k'\in\mathcal{A}^c.$$

Choose any $c_1\in(c_-,c_+)$. The event–fraction condition $\inf_{k\in\mathcal{A}} I_k\ge t_2$ ensures that all $k\in\mathcal{A}_o$ pass the gate $I_k\ge t_2$, while auxiliaries in $\mathcal{A}^c$ are excluded by the D_k threshold (and possibly also by $I_k<t_2$). Hence, $\widehat{C}=\mathcal{A}_o$ w.p. $1-o(1)$, proving the claim. □

Proposition 1 (Correspondence between the threshold rule and the deployed ranking rule)**.** ***[E7]*** *Let the assumptions of Theorem 2 hold, and rank the candidate auxiliary cohorts that pass the event gate $I_k\ge t_2$ in increasing order of a contrast score. Then, with probability tending to one:*

(i) under the penalty-normalized score $D_k/(\lambda_0+\lambda_k)$, every cohort of $\mathcal{A}_o$ precedes every cohort of $\mathcal{A}^c$, so $\mathcal{A}_o$ occupies an initial segment of the ranked list;

(ii) the same holds for the raw score D_k used in practice, provided the penalties are comparable across cohorts in the sense that $(\lambda_0+\lambda_k)/(\lambda_0+\lambda_{k'}) < c_+/c_-$ for all $k\in\mathcal{A}_o$ and $k'\in\mathcal{A}^c$;

(iii) for either score, cutting the ranked list after K elements returns a subset of $\mathcal{A}_o$ whenever $K\le|\mathcal{A}_o|$, and returns $\mathcal{A}_o$ exactly when $K=|\mathcal{A}_o|$. This last cut coincides with the threshold rule of Theorem 2 for every $c_1\in(c_-,c_+)$.

Proof. By Lemmas 4 and 5, with probability tending to one,

$$D_k \ \le\ c_-(\lambda_0+\lambda_k) \quad \text{for all } k\in\mathcal{A}_o, \qquad D_{k'} \ \ge\ c_+(\lambda_0+\lambda_{k'}) \quad \text{for all } k'\in\mathcal{A}^c,$$

and every $k \in \mathcal{A}_o$ passes the event gate, since $\inf_{k\in\mathcal{A}} I_k \geq t_2$. We work on this event throughout.

(i) Dividing each inequality by its own penalty sum places every normalized score of $\mathcal{A}_o$ at or below c_-, and every normalized score of $\mathcal{A}^c$ at or above $c_+ > c_-$. The two groups are therefore separated, and $\mathcal{A}_o$ occupies an initial segment of the ranking.

(ii) Under the penalty-comparability condition, $D_k \leq c_-(\lambda_0 + \lambda_k) < c_+(\lambda_0 + \lambda_{k'}) \leq D_{k'}$ for all $k \in \mathcal{A}_o$ and $k' \in \mathcal{A}^c$, so the raw scores are separated as well and the same initial segment results.

(iii) Both statements about cuts follow from the initial-segment property. For the final claim, a threshold $c_1 \in (c_-, c_+)$ retains exactly the cohorts whose normalized score falls below c_1, which by (i) are exactly those of $\mathcal{A}_o$; this is the event $\widehat{C} = \mathcal{A}_o$ of Theorem 2. □

Read practically, the proposition says that searching over the retained count K replaces the theoretical constants (c_1, t_2) by a single interpretable tuning parameter whose nested search space contains $\mathcal{A}_o$. We do not claim consistency of the data-driven choice of K: the proposition bounds the search space, not the selection made within it, and the stability of the retained sets across replications in the applications provides the corresponding empirical check.

6.3 Proof of Theorem 1

All probability statements are uniform over the parameter space $\Theta(s, h) = \{(\omega^*, \{\delta^{(k)}\}) : \|\omega^*\|_0 \leq s,\ \max_{k\in\mathcal{A}} \|\delta^{(k)}\|_1 \leq h\}$. Recall the joint objective

$$\mathcal{L}(\omega, \delta) \ :=\ \ell_{\mathcal{A}}(\omega) + \frac{1}{n_0 + n_{\mathcal{A}}} \sum_{k\in\mathcal{A}} n_k \{\ell_k(\omega + \delta^{(k)}) - \ell_k(\omega)\} + \lambda_\omega \|\omega\|_1 + \sum_{k\in\mathcal{A}} \lambda_{\delta,k} \|\delta^{(k)}\|_1 + \frac{\mu}{2}\left(\|\omega\|_2^2 + \sum_{k\in\mathcal{A}} \|\delta^{(k)}\|_2^2\right),$$

and let $(\hat{\omega}, \{\hat{\delta}^{(k)}\})$ be any minimizer with the true tuple $(\omega^*, \{\delta^{*(k)}\})$ where ω^* is the target coefficient and $\omega^* + \delta^{*(k)} = w^{(k)}$ is the kth auxiliary-cohort coefficient.

Step 1: Basic inequality and decomposition of Rem. By optimality, $\mathcal{L}(\hat{\omega}, \hat{\delta}) \leq \mathcal{L}(\omega^*, \delta^*)$. Rearranging,

$$\underbrace{\ell_{\mathcal{A}}(\hat{\omega}) - \ell_{\mathcal{A}}(\omega^*)}_{(I)} + \frac{1}{n_0 + n_{\mathcal{A}}} \sum_{k\in\mathcal{A}} n_k \underbrace{\left(\ell_k(\hat{\omega} + \hat{\delta}^{(k)}) - \ell_k(\omega^* + \delta^{*(k)})\right)}_{(II)_k} - \frac{1}{n_0 + n_{\mathcal{A}}} \sum_{k\in\mathcal{A}} n_k \underbrace{\left(\ell_k(\hat{\omega}) - \ell_k(\omega^*)\right)}_{(III)_k}$$
$$+ \lambda_\omega(\|\hat{\omega}\|_1 - \|\omega^*\|_1) + \sum_{k\in\mathcal{A}} \lambda_{\delta,k}(\|\hat{\delta}^{(k)}\|_1 - \|\delta^{*(k)}\|_1) + \frac{\mu}{2}\Big(\|\hat{\omega}\|_2^2 - \|\omega^*\|_2^2 + \sum_{k\in\mathcal{A}} \big(\|\hat{\delta}^{(k)}\|_2^2 - \|\delta^{*(k)}\|_2^2\big)\Big) \ \leq\ 0. \tag{6}$$

By convexity, for any convex ℓ and vectors b, a, $\ell(b) - \ell(a) \geq \langle U(a),\, b - a\rangle$, with $U = \nabla\ell$. Applying this to (I), $(II)_k$, $(III)_k$ gives

$$\begin{aligned}
(I) \ &\geq\ \langle U_{\mathcal{A}}(\omega^*),\, \Delta_\omega\rangle \ +\ \mathsf{Quad}_{\mathcal{A}}(\Delta_\omega),\\
(II)_k \ &\geq\ \langle U_k(\omega^* + \delta^{*(k)}),\, \Delta_\omega + \Delta_\delta^{(k)}\rangle \ +\ \mathsf{Quad}_k(\Delta_\omega + \Delta_\delta^{(k)}),\\
(III)_k \ &\geq\ \langle U_k(\omega^*),\, \Delta_\omega\rangle \ +\ \mathsf{Quad}_k(\Delta_\omega),
\end{aligned}$$

where we write $\Delta_\omega = \hat{\omega} - \omega^*$, $\Delta_\delta^{(k)} = \hat{\delta}^{(k)} - \delta^{*(k)}$, and the quadratic remainders $\mathsf{Quad}_\bullet(\cdot)$ are the second-order terms (Bregman divergences). Plugging into (6) and moving all first-order inner

products to the RHS yields

$$\mathsf{Quad}_{\mathcal{A}}(\Delta_\omega) + \frac{1}{n_0 + n_{\mathcal{A}}} \sum_{k \in \mathcal{A}} n_k \Big(\mathsf{Quad}_k(\Delta_\omega + \Delta_\delta^{(k)}) - \mathsf{Quad}_k(\Delta_\omega) \Big) + \lambda_\omega (\|\hat{\omega}\|_1 - \|\omega^*\|_1) + \sum_{k \in \mathcal{A}} \lambda_{\delta,k} (\|\hat{\delta}^{(k)}\|_1 - \|\delta^{*(k)}\|$$
$$+ \frac{\mu}{2} \Big(\|\hat{\omega}\|_2^2 - \|\omega^*\|_2^2 + \sum_{k \in \mathcal{A}} \big(\|\hat{\delta}^{(k)}\|_2^2 - \|\delta^{*(k)}\|_2^2 \big) \Big) \;\le\; \mathrm{Rem}, \tag{7}$$

with the *remainder*

$$\mathrm{Rem} \;:=\; -\Big\langle U_{\mathcal{A}}(\omega^*),\, \Delta_\omega \Big\rangle - \frac{1}{n_0 + n_{\mathcal{A}}} \sum_{k \in \mathcal{A}} n_k \Big\langle U_k(\omega^* + \delta^{*(k)}),\, \Delta_\omega + \Delta_\delta^{(k)} \Big\rangle + \frac{1}{n_0 + n_{\mathcal{A}}} \sum_{k \in \mathcal{A}} n_k \Big\langle U_k(\omega^*),\, \Delta_\omega \Big\rangle. \tag{8}$$

Step 2: Bounding Rem **by concentration and tuning.** Write $U_{\mathcal{A}}(\omega) = \frac{1}{n_0 + n_{\mathcal{A}}} \big(n_0 U_0(\omega) + \sum_{k \in \mathcal{A}} n_k U_k(\omega) \big)$. For each study k, the Cox score satisfies

$$\mathbb{E}\big[U_k(w^{(k)})\big] = 0, \qquad w^{(k)} = \omega^* + \delta^{*(k)},$$

and under Assumption 1 (bounded/sub-Gaussian covariates) the martingale Bernstein inequality yields [see, e.g., van de Geer, 2000, Chapter 2], for some constants $c, c' > 0$,

$$\Pr\left(\|U_k(w^{(k)})\|_\infty \ge c \sqrt{\frac{\log p}{n_k}} \right) \;\le\; 2p^{-c'}, \qquad \Pr\left(\|U_0(\omega^*)\|_\infty \ge c \sqrt{\frac{\log p}{n_0}} \right) \;\le\; 2p^{-c'}.$$

A union bound over $k \in \{0\} \cup \mathcal{A}$ implies that, with probability $1 - o(1)$,

$$\max_{k \in \{0\} \cup \mathcal{A}} \|U_k(w^{(k)})\|_\infty \;\lesssim\; \sqrt{\frac{\log p}{n_k}}, \qquad \|U_0(\omega^*)\|_\infty \;\lesssim\; \sqrt{\frac{\log p}{n_0}}.$$

Decompose

$$U_{\mathcal{A}}(\omega^*) = \underbrace{\big(U_{\mathcal{A}}(\omega^*) - \mathbb{E}\, U_{\mathcal{A}}(\omega^*) \big)}_{\text{stochastic}} + \underbrace{\mathbb{E}\, U_{\mathcal{A}}(\omega^*)}_{\text{bias from covariate shift}}.$$

Since $E[U_0(\omega^*)] = 0$ (the score is mean-zero at the true parameter), we focus on the auxiliary terms. For $k \in \mathcal{A}$, a first-order Taylor expansion of $E[U_k(\omega)]$ around $w^{(k)} = \omega^* + \delta^{*(k)}$ gives

$$E[U_k(\omega^*)] = E[U_k(w^{(k)})] + H_k(\tilde{\omega}^{(k)})(\omega^* - w^{(k)}) = -H_k(\tilde{\omega}^{(k)})\delta^{*(k)},$$

for some $\tilde{\omega}^{(k)}$ on the line segment between ω^* and $w^{(k)}$, where we used $E[U_k(w^{(k)})] = 0$. Under $\|\delta^{*(k)}\|_1 \le h$ with $h = o(1)$ and bounded covariates (Assumption 1(ii)), the Hessian is Lipschitz in the sense that $\|H_k(\tilde{\omega}^{(k)}) - H_k(\omega^*)\|_\infty = O(h)$. Hence the approximation $E[U_k(\omega^*)] \approx -H_k(\omega^*)\delta^{*(k)}$ introduces only $O(h^2)$ error in each coordinate, which is absorbed into the higher-order terms in the final bound. Hense we have

$$\mathbb{E}\, U_{\mathcal{A}}(\omega^*) = -\frac{1}{n_0 + n_{\mathcal{A}}} \sum_{k \in \mathcal{A}} n_k\, H_k(\omega^*)\, \delta^{*(k)}.$$

Using (8) together with Hölder's inequality and the bounds above, and choosing the tuning

$$\lambda_\omega \;\asymp\; \sqrt{\frac{\log p}{n_0 + n_{\mathcal{A}}}}, \qquad \lambda_{\delta,k} \;\asymp\; \sqrt{\frac{\log p}{n_k}},$$

we obtain, with probability $1 - o(1)$,

$$|\mathrm{Rem}| \;\le\; \lambda_\omega \,\|\Delta_\omega\|_1 + \sum_{k\in\mathcal{A}} \lambda_{\delta,k}\,\|\Delta_\delta^{(k)}\|_1 \;+\; \underbrace{\Big\langle \frac{1}{n_0+n_{\mathcal{A}}} \sum_{k\in\mathcal{A}} n_k\, H_k(\omega^*)\,\delta^{*(k)},\, \Delta_\omega \Big\rangle}_{\text{deterministic bias term}}. \tag{9}$$

We keep the deterministic bias (arising from covariate shift) explicit; it will generate the B_1 term below.

Step 3: Curvature, penalties, and the cone. By Assumption 3 (RSC), the quadratic terms satisfy, for some $\kappa_{\mathcal{A}} > 0$ and all vectors obeying the usual sparsity cone,

$$\mathsf{Quad}_{\mathcal{A}}(\Delta_\omega) \;\ge\; \frac{\kappa_{\mathcal{A}}}{2}\,\|\Delta_\omega\|_2^2, \qquad \mathsf{Quad}_k(\Delta_\omega + \Delta_\delta^{(k)}) - \mathsf{Quad}_k(\Delta_\omega) \;\ge\; -c\,\|\Delta_\omega\|_2\,\|\Delta_\delta^{(k)}\|_2,$$

for a constant $c > 0$ (the cross term can be controlled and absorbed; we suppress it as it only improves curvature due to the ridge). The ridge contributes a nonnegative quadratic in $(\Delta_\omega, \Delta_\delta^{(k)})$ and can be dropped on the LHS (or bounded below by $-\mu(\|\Delta_\omega\|_2^2 + \sum_k \|\Delta_\delta^{(k)}\|_2^2)/2$, which is dominated since $\mu \lesssim (n_0 + n_{\mathcal{A}})^{-1}$). For the penalties, use

$$\begin{aligned}
\lambda_\omega(\|\hat\omega\|_1 - \|\omega^*\|_1) \;&\ge\; -\lambda_\omega\big(\|\Delta_{\omega,S}\|_1 - \|\Delta_{\omega,S^c}\|_1\big)\\
\sum_k \lambda_{\delta,k}(\|\hat\delta^{(k)}\|_1 - \|\delta^{*(k)}\|_1) \;&\ge\; -\sum_k \lambda_{\delta,k}\,\|\Delta_\delta^{(k)}\|_1 - \underbrace{\sum_k \lambda_{\delta,k}\|\delta^{*(k)}\|_1}_{\mathsf{B}_2}
\end{aligned}$$

where $S = \mathrm{supp}(\omega^*)$, and we defined $\mathsf{B}_2 := \sum_{k\in\mathcal{A}} \lambda_{\delta,k}\|\delta^{*(k)}\|_1$.

Insert these bounds and (9) into (7), cancel the stochastic $\|\Delta_\delta^{(k)}\|_1$ terms, and collect like terms to get

$$\frac{\kappa_{\mathcal{A}}}{2}\,\|\Delta_\omega\|_2^2 \;\le\; \lambda_\omega\big(\|\Delta_{\omega,S}\|_1 - \|\Delta_{\omega,S^c}\|_1\big) \;+\; \lambda_\omega\,\|\Delta_\omega\|_1 \;+\; \mathsf{B}_2 \;+\; \Big\langle \bar H_{\mathcal{A}}(\omega^*)\,\bar\delta,\, \Delta_\omega \Big\rangle, \tag{10}$$

where we introduced the weighted average $\bar H_{\mathcal{A}}(\omega^*) := \frac{1}{n_0+n_{\mathcal{A}}} \sum_{k\in\mathcal{A}} n_k H_k(\omega^*)$ and $\bar\delta$ is the block vector whose kth block is $\delta^{*(k)}$. The last inner product is the deterministic bias carried over from (9).

Standard algebra (using $\|\Delta_\omega\|_1 = \|\Delta_{\omega,S}\|_1 + \|\Delta_{\omega,S^c}\|_1$ and $\lambda_\omega \asymp \sqrt{\log p/(n_0+n_{\mathcal{A}})}$) yields the *cone condition*

$$\|\Delta_{\omega,S^c}\|_1 \;\le\; 3\,\|\Delta_{\omega,S}\|_1,$$

so that $\|\Delta_\omega\|_1 \le 4\|\Delta_{\omega,S}\|_1 \le 4\sqrt{s}\,\|\Delta_\omega\|_2$. Substituting this into (10), we obtain

$$\kappa_{\mathcal{A}}\,\|\Delta_\omega\|_2^2 \;\lesssim\; \lambda_\omega\,\sqrt{s}\,\|\Delta_\omega\|_2 \;+\; \mathsf{B}_2 \;+\; \Big\langle \bar H_{\mathcal{A}}(\omega^*)\,\bar\delta,\, \Delta_\omega \Big\rangle, \tag{11}$$

and hence

$$\kappa_{\mathcal{A}}\,\|\Delta_\omega\|_2^2 \;\lesssim\; \lambda_\omega^2\, s \;+\; \mathsf{B}_2 \;+\; \Big\langle \bar H_{\mathcal{A}}(\omega^*)\,\bar\delta,\, \Delta_\omega \Big\rangle.$$

Step 4: Identifying B_1 and handling heterogeneity. Multiply both sides by $\frac{n_0+n_{\mathcal{A}}}{n_0}$ and replace $\bar{H}_{\mathcal{A}}$ by H_0 plus a controllable difference:

$$\frac{n_0+n_{\mathcal{A}}}{n_0}\left\langle \bar{H}_{\mathcal{A}}(\omega^*)\,\bar{\delta},\,\Delta_\omega\right\rangle = \frac{1}{n_0}\sum_{k\in\mathcal{A}} n_k\left\langle H_0(\omega^*)\,\delta^{*(k)},\,\Delta_\omega\right\rangle + \frac{1}{n_0}\sum_{k\in\mathcal{A}} n_k\left\langle (H_k(\omega^*)-H_0(\omega^*))\,\delta^{*(k)},\,\Delta_\omega\right\rangle.$$

Define

$$\mathsf{B}_1 \;:=\; \frac{1}{n_0}\sum_{k\in\mathcal{A}} n_k\left\langle H_0(\omega^*)\,\delta^{*(k)},\,\Delta_\omega\right\rangle.$$

By Assumption 4, the heterogeneity factor $C_\Sigma(\mathcal{A}) = O(1)$ and $\|H_k(\omega^*) - H_0(\omega^*)\|_\infty \le c_H$. Using Hölder and the sparsity cone bound $\|\Delta_\omega\|_1 \lesssim \sqrt{s}\|\Delta_\omega\|_2$ together with $\|\delta^{*(k)}\|_1 \le h$,

$$\left|\frac{1}{n_0}\sum_{k\in\mathcal{A}} n_k\left\langle (H_k - H_0)\,\delta^{*(k)},\,\Delta_\omega\right\rangle\right| \;\lesssim\; C_\Sigma(\mathcal{A})\,h\,\sqrt{\frac{\log p}{n_0}}\;\|\Delta_\omega\|_2,$$

where we used that H_0-weighted coordinates are estimated at the $\sqrt{\log p/n_0}$ scale on the cone (this follows from the same concentration that sets λ_ω). Absorbing the last display into the LHS by RSC (an $\varepsilon\|\Delta_\omega\|_2^2$ argument) yields

$$\kappa_{\mathcal{A}}\,\|\Delta_\omega\|_2^2 \;\lesssim\; \lambda_\omega^2 s \;+\; \mathsf{B}_1 \;+\; \mathsf{B}_2,$$

which is the claimed intermediate inequality.

Step 5: Final bounds for B_2 and B_1. Since $\|\delta^{*(k)}\|_1 \le h$ and $\lambda_{\delta,k} \asymp \sqrt{\log p/n_k}$,

$$\mathsf{B}_2 = \sum_{k\in\mathcal{A}} \lambda_{\delta,k}\|\delta^{*(k)}\|_1 \;\lesssim\; h\sum_{k\in\mathcal{A}}\sqrt{\frac{\log p}{n_k}} \;\lesssim\; h\sqrt{\frac{\log p}{n_0}},$$

after normalization by n_0 (the unbalanced case is handled by the explicit weights). For B_1, by Cauchy–Schwarz,

$$|\mathsf{B}_1| \;\le\; \left\|\frac{1}{n_0}\sum_{k\in\mathcal{A}} n_k\,H_0(\omega^*)\,\delta^{*(k)}\right\|_2 \|\Delta_\omega\|_2 \;\lesssim\; C_\Sigma(\mathcal{A})\,h\sqrt{\frac{\log p}{n_0}}\;\|\Delta_\omega\|_2,$$

and we absorb this linear-in-$\|\Delta_\omega\|_2$ term into the LHS via $ab \le (\kappa_{\mathcal{A}}/4)a^2 + c\,b^2$ to obtain the second bracket term in (5). The first bracket term $\lambda_\omega^2 s \asymp s\log p/(n_0+n_{\mathcal{A}})$ follows from Step 4. The target Bregman risk bound (the other part of (5)) is obtained from the same expansion by keeping the quadratic $\mathsf{Quad}_0(\Delta_\omega)$ term on the LHS. This completes the proof. □

6.4 Auxiliary Lemmas

6.4.1 Estimation and screening lemmas

Lemma 1 (Score concentration and tuning)**.** *Under Assumption 1, for each $k \in \{0\}\cup\mathcal{A}$ and any $t > 0$, $\Pr\left(\|U_k(\omega^*)\|_\infty \ge c\sqrt{\log p/n_k} + t\right) \le 2\exp(-c' n_k t^2)$. Consequently, choosing $\lambda_\omega \asymp \sqrt{\log p/(n_0+n_{\mathcal{A}})}$ and $\lambda_{\delta,k} \asymp \sqrt{\log p/n_k}$ ensures the basic inequality with high probability.*

Lemma 2 (RSC for Cox under bounded covariates)**.** *Under Assumptions 1 and 3, the local quadratic approximation of ℓ_k satisfies the restricted strong convexity on $\mathcal{N}(\omega^*, r)$ with high probability: $v^\top H_k(\omega)v \ge \kappa_k\|v\|_2^2$ for $\|v_{S^c}\|_1 \le 3\|v_S\|_1$; similarly for $\bar{H}_{\mathcal{A}}$ with parameter $\kappa_{\mathcal{A}}$[Huang et al., 2013, Kong and Nan, 2014].*

Lemma 3 (Consistency of the proof-side marginal divergence statistic)**.** *Let $\widehat{R}^{(k)}$ be the proof-side marginal divergence statistic defined in §7.1. With $t^* = \lceil n_*^{\alpha} \rceil$, $\alpha \in (0, 1)$, and the separation conditions in Lemma 4, the SURE-type screening step recovers H_k for all $k \in \mathcal{A}^c$ with probability $1 - o(1)$ and excludes most noise coordinates for $k \in \mathcal{A}$. Consequently, $\widehat{R}^{(k)}$ concentrates around $\|\Delta m^{(k)}_{\widehat{T}_k}\|_2^2$ within $O_p((\log p)/n_*^{1-\alpha})$. This is a proof-side screening device adapted from the SURE-screening framework of Fan and Lv [2008].*

Proofs of auxiliary lemmas. We provide proof sketches here; complete arguments paralleling those in Huang et al. [2013] and Fang et al. [2017] can be found in a supplementary document.

Proof of Lemma 1. The Cox partial-likelihood score $U_k(\omega)$ evaluated at the true study-k coefficient $\omega^{(k)}$ is a sum of mean-zero martingale increments with respect to the filtration generated by the counting process $N_i^{(k)}(t) = \mathbf{1}\{T_i^{(k)} \le t, \Delta_i^{(k)} = 1\}$. Under bounded covariates $\|X^{(k)}\|_\infty \le K_X$, each increment has bounded range $O(K_X)$. Applying the Bernstein inequality for sums of martingale differences [e.g., van de Geer, 2000, Chapter 2], we obtain, for each coordinate j,

$$P\big(|U_{k,j}(\omega^{(k)})| \ge t\big) \le 2\exp\Big(-\frac{c\, n_k t^2}{K_X^2}\Big).$$

A union bound over $j \in [p]$ and the choice $t = c'\sqrt{(\log p)/n_k}$ yields the stated high-probability bound $\|U_k(\omega^{(k)})\|_\infty \lesssim \sqrt{(\log p)/n_k}$.

Proof of Lemma 2. The Hessian $H_k(\omega) = \nabla^2 \ell_k(\omega)$ of the negative Cox partial log-likelihood equals the risk-set–weighted variance of the covariates at event times. Specifically,

$$H_k(\omega) = \frac{1}{n_k} \sum_{i:\Delta_i^{(k)}=1} \mathrm{Var}_{R_i}\big(X^{(k)}\big),$$

where Var_{R_i} denotes the variance under the distribution that places mass proportional to $\exp(X_j^{(k)\top}\omega)$ on each subject j in the risk set at time $T_i^{(k)}$. Under bounded covariates and Assumption 3, the minimum eigenvalue of $H_k(\omega)$ restricted to s-sparse directions is bounded below by $\kappa_k > 0$ with high probability; see Huang et al. [2013, Lemma 3] for a detailed argument. The pooled RSC for $\bar{H}_{\mathcal{A}}$ follows by convexity.

Proof of Lemma 3. SURE-screening selects the $t^* = \lceil n_*^{\alpha} \rceil$ coordinates with largest marginal statistics. Under the separation condition that incompatible auxiliary cohorts have at least $|H_k| \gtrsim n_*^{1-\alpha}$ coordinates with population marginal score difference exceeding a threshold, standard sub-Gaussian tail bounds ensure that the empirical ranking preserves the population ranking with probability $1 - o(1)$. The argument follows Fan and Lv [2008], adapted to the partial-likelihood score setting.

6.4.2 Practical screening lemmas

Lemma 4 (Consistent transferable-set selection for the target-centered Cox model)**.** *Assume 1, and that for each non-informative auxiliary $k \in \mathcal{A}^c$ there exists a set of strong marginals $H_k \subset [p]$ with $|H_k| \gtrsim n_*^{1-\alpha}$ such that*

$$\sum_{j \in H_k} \Big| \big(\mathbb{E}[\Delta X_j \mid k] - \mathbb{E}[\Delta X_j \mid 0]\big) \Big|^2 \;\ge\; c\, \frac{\log p}{n_*^{1-\alpha}},$$

for some constant $c > 0$, while for $k \in \mathcal{A}$, $\|H_0(\omega^*)\,\delta^{(k)}\|_2^2 \le c' \frac{\log p}{n_*^{1-\alpha}}$ for a sufficiently small c'. Then, with $\widehat{R}^{(k)}$ constructed as in §7.1 and any finite grid of t^* values, there exists a nonempty $\mathcal{A}_o \subseteq \mathcal{A}$ such that

$$\Pr\Big(\max_{k\in\mathcal{A}_o} \widehat{R}^{(k)} \;<\; \min_{k\in\mathcal{A}^c} \widehat{R}^{(k)} \Big) \to 1.$$

Consequently, the proof-side marginal-divergence ranking separates $\mathcal{A}_o$ from $\mathcal{A}^c$ with probability $1 - o(1)$.

Proof. Proof of Lemma 4
Let $\Delta m^{(k)} := \mathbb{E}[\Delta X \mid k] - \mathbb{E}[\Delta X \mid 0]$ denote the population marginal partial-score difference. For $k \in \mathcal{A}^c$, by assumption there exists H_k so that $\|\Delta m^{(k)}_{H_k}\|_2^2 \gtrsim (\log p)/n_*^{1-\alpha}$. For $k \in \mathcal{A}$, $\|\Delta m^{(k)}\|_\infty \lesssim \|H_0(\omega^*)\delta^{(k)}\|_\infty \le c' n_*^{-(1-\alpha)/2}\sqrt{\log p}$. SURE-screening ensures that with probability $1 - o(1)$ the set $\widehat{T}_k$ contains H_k for $k \in \mathcal{A}^c$ and includes only $O(t^*)$ indices for $k \in \mathcal{A}$. Standard maximal inequalities for (sub-)Gaussian processes and union bounds over p coordinates yield

$$\big|\widehat{R}^{(k)} - \|\Delta m^{(k)}_{\widehat{T}_k}\|_2^2\big| \;=\; O_p\Big(\frac{\log p}{n_*^{1-\alpha}}\Big).$$

Thus, for $k \in \mathcal{A}^c$, $\widehat{R}^{(k)} \ge c\,(\log p)/n_*^{1-\alpha} + o_p((\log p)/n_*^{1-\alpha})$, while for $k \in \mathcal{A}$, $\widehat{R}^{(k)} \le c'\,(\log p)/n_*^{1-\alpha} + o_p((\log p)/n_*^{1-\alpha})$ with $c' < c$. Hence the marginal-divergence ranking separates some nonempty $\mathcal{A}_o \subseteq \mathcal{A}$ from $\mathcal{A}^c$ with probability $1 - o(1)$. □

Lemma 5 (Equivalence of auxiliary-cohort screening diagnostics)**.** *Let $\widehat{C}$ be the set selected by the practical screening rule $\widehat{C} = \{\, k : \;\; D_k \le c_1(\lambda_0 + \lambda_k) \;\wedge\; I_k \ge t_2 \,\}$ with $D_k := \|\widehat{\delta}^{(k)}\|_1$ from the initial joint fit (2), and $I_k := n_k^{-1}\sum_{i=1}^{n_k} \Delta_i^{(k)}$. Let $\widehat{R}^{(k)}$ be the proof-side marginal-divergence statistic defined in §7.1. Suppose Assumptions 1–4 hold, the penalties satisfy $\lambda_0 \asymp \sqrt{\log p/n_0}$ and $\lambda_k \asymp \sqrt{\log p/n_k}$, and the event-fraction gate obeys $\inf_{k\in\mathcal{A}} I_k \ge t_2 > 0$. Then there exist constants $0 < c_- < c_+ < \infty$ and a nonempty oracle subset $\mathcal{A}_o \subseteq \mathcal{A}$ such that, with probability $1 - o(1)$,*

$$\max_{k\in\mathcal{A}_o} \widehat{R}^{(k)} < \min_{k\in\mathcal{A}^c} \widehat{R}^{(k)} \quad\Longrightarrow\quad \max_{k\in\mathcal{A}_o} D_k \le c_-(\lambda_0 + \lambda_k) \;\; \text{and} \;\; \min_{k\in\mathcal{A}^c} D_k \ge c_+(\lambda_0 + \lambda_k).$$

In particular, if Lemma 4 holds for $\{\widehat{R}^{(k)}\}$, then the practical screening rule recovers the same transferable subset $\mathcal{A}_o$ w.p. $1 - o(1)$.

Proof. Proof of Lemma 5
Step 1 (KKT for the initial joint fit). For the optimization (2) on $\{0\} \cup \{1, \ldots, K\}$, the subgradient condition for the block $\delta^{(k)}$ is

$$U_k(\widehat{\omega}_0 + \widehat{\delta}^{(k)}) + \lambda_k\,\widehat{z}^{(k)} + \mu\,\widehat{\delta}^{(k)} = 0, \qquad \|\widehat{z}^{(k)}\|_\infty \le 1,$$

with equality on the support of $\widehat{\delta}^{(k)}$; for coordinates with $\widehat{\delta}^{(k)}_j = 0$ this yields $\|U_k(\widehat{\omega}_0)\|_\infty \le \lambda_k$ (up to the vanishing μ term). Similarly, the KKT condition for ω_0 gives $\|U_0(\widehat{\omega}_0)\|_\infty \le \lambda_0$.

Step 2 (Link between $U_k - U_0$, divergence, and shift). By a first–order expansion around ω^* and the RSC/compatibility assumptions,

$$U_k(\widehat{\omega}_0) - U_0(\widehat{\omega}_0) = \underbrace{H_0(\omega^*)\,\delta^{(k)}}_{\text{population shift}} \;+ r_k,$$

where $\|r_k\|_\infty = O_p\big(\sqrt{\frac{\log p}{n_k}} + \sqrt{\frac{\log p}{n_0}} + C_\Sigma(\mathcal{A})\, h\sqrt{\frac{\log p}{n_0}}\big)$. Moreover, $\widehat{R}^{(k)}$ concentrates (Lemma 3) around the squared norm $\|\Delta m^{(k)}_{\widehat{T}_k}\|_2^2$, and $\Delta m^{(k)}$ is the marginal version of $U_k(\omega^*) - U_0(\omega^*)$, hence scales with $\|H_0(\omega^*)\,\delta^{(k)}\|$.

Step 3 (From divergence separation to D_k–separation). On the event in Lemma 4, there is a subset $\mathcal{A}_o \subseteq \mathcal{A}$ such that $\max_{k\in\mathcal{A}_o} \widehat{R}^{(k)} < \min_{k\in\mathcal{A}^c} \widehat{R}^{(k)}$. By Step 2, this implies a separation of the gradients $\|U_k(\widehat{\omega}_0) - U_0(\widehat{\omega}_0)\|_\infty$ between $\mathcal{A}_o$ and $\mathcal{A}^c$, up to stochastic errors of order $\lambda_0+\lambda_k$. Using the KKT bounds $\|U_0(\widehat{\omega}_0)\|_\infty \le \lambda_0$ and the triangle inequality, we obtain constants $0 < c_- < c_+$ such that w.h.p.

$$\max_{k\in\mathcal{A}_o} \|U_k(\widehat{\omega}_0)\|_\infty \le c_-(\lambda_0 + \lambda_k), \qquad \min_{k\in\mathcal{A}^c} \|U_k(\widehat{\omega}_0)\|_\infty \ge c_+(\lambda_0 + \lambda_k).$$

Invoking the blockwise KKT condition for $\delta^{(k)}$ (Step 1) and the event–fraction gate $I_k \ge t_2$ (which guarantees curvature so that small gradient implies small $\widehat{\delta}^{(k)}$), we translate the gradient separation into the stated D_k separation. Finally, choosing $c_1 \in (c_-, c_+)$ yields the stated equivalence between the proof-side marginal separation event and the practical D_k-based screening rule. □

Remark (Heterogeneity penalty). When $C_\Sigma(\mathcal{A})$ is not $O(1)$, the second bracket in (5) acquires factors $C_\Sigma(\mathcal{A})$ and $C_\Sigma(\mathcal{A})^2$ accordingly. The practical screening theorem retains the same form, but larger heterogeneity makes beneficial transfer harder to realize in finite samples.

7 Algorithms and computational details for TransSurv

This section records the computational details needed to reproduce the TransSurv fitting procedure. To keep the distinction between theory and implementation clear, we first describe the proof-side marginal divergence statistic used only in the screening argument, and then present the practical solver used to fit the joint target-centered Cox objective after auxiliary cohorts have been retained. This organization mirrors the main TransSurv pipeline: compatibility is assessed first, and target-centered joint estimation is performed only after harmful borrowing has been filtered out.

7.1 Proof-side marginal divergence statistic for screening

The screening proof in §6 uses a marginal divergence statistic to separate compatible from incompatible auxiliary cohorts. This statistic is a proof-side device rather than a deployed component of the TransSurv pipeline. In practice, retained auxiliary cohorts are selected by the fitted contrast diagnostic D_k together with the event-information threshold; the construction below is included only to make the selection argument explicit.

For each auxiliary k, let $\Delta\widehat{m}^{(k)} \in \mathbb{R}^p$ be the difference between auxiliary and target *marginal partial scores* (evaluated at a target-only pilot $\tilde{\omega}$),

$$\Delta\widehat{m}^{(k)} := \frac{1}{n_k}\sum_{i=1}^{n_k}\Big\{\Delta_i^{(k)} X_i^{(k)} - \hat{\mathbb{E}}\big[X^{(k)} \,\big|\, T^{(k)} \ge T_i^{(k)}\big]\Delta_i^{(k)}\Big\} - \frac{1}{n_0}\sum_{i=1}^{n_0}\Big\{\Delta_i^{(0)} X_i^{(0)} - \hat{\mathbb{E}}\big[X^{(0)} \,\big|\, T^{(0)} \ge T_i^{(0)}\big]\Delta_i^{(0)}\Big\}.$$

For this proof-side construction, apply a SURE-type screening step: for a threshold $t^* = \lceil n_*^\alpha \rceil$ with $n_* := \min_k n_k$ and fixed $\alpha \in (0,1)$, pick the t^* largest absolute components to form $\widehat{T}_k$, and define

$$\widehat{R}^{(k)} := \big\|\Delta\widehat{m}^{(k)}_{\widehat{T}_k}\big\|_2^2, \quad k = 1, \ldots, K.$$

The ranked statistics $\widehat{R}^{(k)}$ are used only to connect marginal score separation with the practical fitted-contrast diagnostic D_k in the screening proofs; they are not part of the deployed TransSurv fitting routine.

7.2 Target-centered Cox model solver in TransSurv

After the retained auxiliary set has been chosen, TransSurv fits the joint penalized Cox objective by alternating updates of the shared target-centered coefficient vector and the auxiliary-specific contrast vectors. This decomposition is the computational counterpart of the model design in the main text: the shared block borrows information across the target and retained auxiliary cohorts, whereas the contrast blocks allow sparse cohort-specific correction and thereby avoid naive pooling. The algorithm below summarizes the routine used in our implementation.

Algorithm 2 Target-centered Cox model Solver (proximal coordinate descent)

Require: Datasets $\{(T^{(k)}, \Delta^{(k)}, X^{(k)})\}_{k=0}^{K}$; penalties λ and $\{\lambda_k\}_{k=1}^{K}$ (with $\delta^{(0)} \equiv 0$); tolerance $\varepsilon > 0$; maximum passes $M_{\max}$; curvature floor $\epsilon_{\text{curv}} > 0$

Ensure: $\widehat{\omega}_0$, $\{\widehat{\delta}^{(k)}\}_{k=1}^{K}$, and per–study $\widehat{\omega}^{(k)} = \widehat{\omega}_0 + \widehat{\delta}^{(k)}$

Preprocess: Sort by decreasing time within each study k; store order $\pi^{(k)}$ and tie sets $\mathcal{G}^{(k)}(t) = \{i : T_i^{(k)} = t,\ \Delta_i^{(k)} = 1\}$ with $d_k(t) = |\mathcal{G}^{(k)}(t)|$.

Initialize: $\widehat{\omega}_0 \leftarrow 0$, $\widehat{\delta}^{(k)} \leftarrow 0$ for $k \geq 1$. For each k: $\eta^{(k)} \leftarrow X^{(k)}\widehat{\omega}_0 + X^{(k)}\widehat{\delta}^{(k)} = 0$.

for $m = 1$ **to** $M_{\max}$ **do**

  Update shared block ω_0

  for $j = 1$ **to** p **do**

    Offsets: for each k, set $o^{(k)} \leftarrow X^{(k)}\widehat{\delta}^{(k)}$ and $\eta^{(k)} \leftarrow o^{(k)} + X^{(k)}\widehat{\omega}_0$.

    Risk-set aggregates (Breslow ties): for each k and event time t,

$$S_{0k}(t) = \sum_i Y_i^{(k)}(t)\, e^{\eta_i^{(k)}}, \quad S_{1k,j}(t) = \sum_i Y_i^{(k)}(t)\, e^{\eta_i^{(k)}} X_{ij}^{(k)}, \quad S_{2k,j}(t) = \sum_i Y_i^{(k)}(t)\, e^{\eta_i^{(k)}} \big(X_{ij}^{(k)}\big)^2.$$

    Gradient and curvature for w_{0j}:

$$g_{0j} \;=\; \sum_{k=0}^{K} \left[-\frac{1}{n_k} \sum_{i:\,\Delta_i^{(k)}=1} \left(X_{ij}^{(k)} - \frac{S_{1k,j}(T_i^{(k)})}{S_{0k}(T_i^{(k)})} \right) \right], h_{0j} \;=\; \sum_{k=0}^{K} \left[\frac{1}{n_k} \sum_t d_k(t) \left(\frac{S_{2k,j}(t)}{S_{0k}(t)} - \left(\frac{S_{1k,j}(t)}{S_{0k}(t)} \right)^2 \right) \right].$$

  Set $h_{0j} \leftarrow \max\big(h_{0j}, \epsilon_{\text{curv}}\big)$.

    Proximal Newton update:

$$\tilde{z} \leftarrow \widehat{w}_{0j} - g_{0j}/h_{0j}, \qquad \widehat{\omega}_{0j}^{\text{new}} \leftarrow \operatorname{sign}(\tilde{z})\,\big(|\tilde{z}| - \lambda/h_{0j}\big)_+.$$

    Rank-1 refresh: $\Delta\omega \leftarrow \widehat{\omega}_{0j}^{\text{new}} - \widehat{\omega}_{0j}$; set $\widehat{\omega}_{0j} \leftarrow \widehat{\omega}_{0j}^{\text{new}}$ and for each k update $\eta^{(k)} \leftarrow \eta^{(k)} + X_{\cdot j}^{(k)}\,\Delta\omega$.

  end for

  Update each auxiliary block $\delta^{(k)}$

  for $k = 1$ **to** K **do**

    for $j = 1$ **to** p **do**

      Offset and predictor: $o^{(k)} \leftarrow X^{(k)}\widehat{\omega}_0$, $\eta^{(k)} \leftarrow o^{(k)} + X^{(k)}\widehat{\delta}^{(k)}$.

      Risk-set aggregates: compute $S_{0k}(t)$, $S_{1k,j}(t)$, $S_{2k,j}(t)$ as above.

      Gradient and curvature for $\delta_j^{(k)}$:

$$g_{k,j} \;=\; -\frac{1}{n_k} \sum_{i:\,\Delta_i^{(k)}=1} \left(X_{ij}^{(k)} - \frac{S_{1k,j}(T_i^{(k)})}{S_{0k}(T_i^{(k)})} \right), h_{k,j} \;=\; \frac{1}{n_k} \sum_t d_k(t) \left(\frac{S_{2k,j}(t)}{S_{0k}(t)} - \left(\frac{S_{1k,j}(t)}{S_{0k}(t)} \right)^2 \right).$$

  Set $h_{k,j} \leftarrow \max\big(h_{k,j}, \epsilon_{\text{curv}}\big)$.

      Proximal Newton update:

$$\tilde{z} \leftarrow \widehat{\delta}_j^{(k)} - g_{k,j}/h_{k,j}, \qquad \widehat{\delta}_j^{(k)\,\text{new}} \leftarrow \operatorname{sign}(\tilde{z})\,\big(|\tilde{z}| - \lambda_k/h_{k,j}\big)_+.$$

      Rank-1 refresh (study k): $\Delta\delta \leftarrow \widehat{\delta}_j^{(k)\,\text{new}} - \widehat{\delta}_j^{(k)}$; set $\widehat{\delta}_j^{(k)} \leftarrow \widehat{\delta}_j^{(k)\,\text{new}}$ and $\eta^{(k)} \leftarrow \eta^{(k)} + X_{\cdot j}^{(k)}\,\Delta\delta$.

    end for

  end for

  Stopping criteria: if $|\mathcal{L}^{(m)} - \mathcal{L}^{(m-1)}| \leq \varepsilon\,(1 + \mathcal{L}^{(m-1)})$.

end for

Return: $\widehat{\omega}_0$, $\{\widehat{\delta}^{(k)}\}_{k=1}^{K}$, and $\{\widehat{\omega}^{(k)}\}_{k=0}^{K}$ with $\widehat{\omega}^{(k)} = \widehat{\omega}_0 + \widehat{\delta}^{(k)}$ and $\widehat{\delta}^{(0)} \equiv 0$.

Operationally, the routine behaves like a target-centered stratified Cox fit with offsets: when the shared block is updated, the current auxiliary-specific deviations are treated as fixed offsets,

and when a auxiliary-specific contrast block is updated, the shared target coefficient is held fixed.

Remark 1 (Implementation notes for Algorithm 2)**.**

1. ***Computational complexity.*** *Let $\widehat{\mathcal{A}}$ denote the retained auxiliary set and let*

$$n_{\text{ret}} = n_0 + \sum_{k \in \widehat{\mathcal{A}}} n_k$$

be the total number of observations entering the final TransSurv fit. After sorting event times once within each study, a full sweep of Algorithm 2 is dominated by repeated formation of study-wise risk-set sums and coordinate updates. The shared-block update for ω_0 costs approximately

$$O(p\, n_{\text{ret}})$$

per sweep, since it uses the target cohort together with all retained auxiliary cohorts. The contrast-block updates contribute an additional

$$O\left(p \sum_{k \in \widehat{\mathcal{A}}} n_k\right),$$

because each retained auxiliary cohort is revisited to update its sparse deviation vector. Thus, up to modest implementation-dependent constants, the overall per-sweep cost is

$$O\left(p\Big(n_0 + 2\sum_{k \in \widehat{\mathcal{A}}} n_k\Big)\right).$$

This clarifies the comparison with the two most natural baselines. A target-only penalized Cox fit has per-sweep cost $O(pn_0)$, so TransSurv pays the expected additional cost of processing the retained auxiliary cohorts. A naive pooled fit over the same retained data has per-sweep cost roughly $O(p\, n_{\text{ret}})$, and is therefore somewhat cheaper than TransSurv because it estimates only one shared coefficient vector rather than a shared target effect together with sparse cohort-specific corrections. In the applications considered here, however, the retained set is small after screening, so the extra computation is typically modest relative to the gain in robustness against harmful transfer.

2. ***Unpenalized baseline covariates.****Algorithm above only displays updates for the penalized genomic component. Unpenalized baseline covariates can be incorporated by augmenting the linear predictor with a low-dimensional term $B_i^{(k)\top}\alpha^{(k)}$ and alternating between Newton–Raphson updates for the unpenalized coefficients and the same proximal coordinate updates for ω_0 and $\{\delta^{(k)}\}$, treating whichever block is held fixed as an offset.*

3. ***Offsets and target anchoring.*** *The updates are exactly those of a stratified Cox model with per-subject offsets: when updating w_0, use offset $o^{(k)} = X^{(k)}\widehat{\delta}^{(k)}$; when updating $\delta^{(k)}$, use $o^{(k)} = X^{(k)}\widehat{w}_0$. The target study remains anchored by $\delta^{(0)} \equiv 0$ so $w^{(0)} = w_0$.*

4. ***Efficient risk-set sums.*** *With subjects sorted by decreasing time, compute $S_{0k}, S_{1k,j}, S_{2k,j}$ via cumulative sums along $\pi^{(k)}$; handle ties with Breslow's approximation as written. Cache $e^{\eta^{(k)}}$ and refresh incrementally after each rank-1 update.*

5. ***Penalty calibration and standardization.*** *Standardize covariates within study. Use* $\lambda \asymp \sqrt{\log p/n_0}$ *and* $\lambda_k \asymp \sqrt{\log p/n_k}$ *as baseline rates, with constants chosen by cross-validation. Auxiliary-specific penalties may be calibrated asymmetrically when expected auxiliary–target mismatch differs across data sources. In the FUSCC TNBC application, the TCGA deviation penalty was set fourfold larger than the METABRIC deviation penalty, reflecting the stronger measurement mismatch of the microarray-derived METABRIC cohort.*

6. ***Numerical safeguards.*** *The curvature floor* ϵ_{curv} *(e.g.,* 10^{-8}*) prevents division by near-zero Hessians; clipping very large step proposals early can improve stability. If overflow occurs in* e^{η}*, shift predictors by subtracting a per-time maximum (stabilizes softmax-like sums).*

7. ***Warm starts, paths, and screening.*** *A first pass with* $\widehat{\delta}^{(k)} \equiv 0$ *is a strong warm start. Fit along a decreasing penalty path with warm starts for speed. SAFE/strong rules can pre-screen coordinates with* $|g|$ *below their penalty, substantially reducing work.*

8. ***Parallelization and convergence.*** *The auxiliary blocks* $\{\delta^{(k)}\}$ *can be updated in parallel after each* w_0 *sweep (their gradients depend only on study-k data given the current* w_0*). Use the KKT residual test in the algorithm; optionally add a duality-gap check if implemented.*

7.3 Practical tuning and implementation details for real-data analyses

This subsection records implementation choices that are specific to the real-data TransSurv analyses and are omitted from the main text for readability. The target-centered model, practical screening rule, and solver are given in Methods Section 4.4, Sections 1 and 7.2. The purpose of the present subsection is to document how the regularization grids and retained auxiliary cohorts were tuned in practice.

Training-side tuning principle. All tuning decisions were made strictly within the target training split. The held-out target test split was never used to define the candidate penalty ranges, select retained auxiliary cohorts, choose the final tuning parameters, or modify solver settings. To stabilize the tuning search and avoid excessive per-replication optimization, we used a two-stage strategy. First, for each application, we selected one representative target training split using a fixed random seed and carved out an internal validation subset from that training data. This pilot run was used only to identify a feasible range of regularization values for TransSurv. Second, once the candidate grids had been fixed for that application, retained auxiliary cohorts and penalty values were selected using five-fold cross-validation on the target training data only. For the TCGA analyses, this five-fold cross-validation step was carried out separately within each target train–test split, so that the retained set and final penalty values were allowed to adapt to the split-specific target signal. For the JAVELIN Renal 101 and FUSCC TNBC analyses, by contrast, we first performed five-fold cross-validation on one random target training split to identify a stable set of penalty values, and then fixed those values across the remaining train–test replications. This choice was motivated by the greater empirical stability of the retained auxiliary cohorts in these two applications, the larger amount of information available in both the target and auxiliary data, and the desire to avoid over-fitting the tuning procedure to repeated splits of the same cohort.

Candidate penalty grids. For the JAVELIN Renal 101 clinical-trial analysis, candidate values for the TransSurv shared penalty λ_ω and the auxiliary-specific penalties $\{\lambda_{\delta,k}\}$ were taken from a fine linear grid on $[0.01, 1]$. We rounded candidate values to two decimals, and considered 15 equally spaced values. For the target-only and naive-pooling comparators in this application, we

widened the corresponding grid range to $[0.01, 2]$ to allow stronger shrinkage in view of the smaller effective sample size.

For the TCGA and FUSCC analyses, candidate values were taken from the logarithmically spaced multiplier grid

$$\{0.01,\ 0.03,\ 0.1,\ 0.3,\ 1,\ 3,\ 10,\ 30,\ 100,\ 300\}.$$

Similarly, we widened the corresponding grid range to 3000 for the target-only and naive-pooling comparators in this application. These quantities are not the raw penalty values themselves, but multiplicative factors applied to the corresponding theoretical penalty rates; see Section 6.2 for the precise rate definitions. The shared penalty λ_ω and the auxiliary-specific penalties $\{\lambda_{\delta,k}\}$ were tuned over the corresponding candidate grids. For the FUSCC TNBC application, the auxiliary-specific penalties were further constrained to satisfy $\lambda_{\delta,\text{TCGA}} = 4\,\lambda_{\delta,\text{METABRIC}}$.

Selection of retained auxiliary cohorts. **[E8]** Within each replication, candidate auxiliary cohorts were ranked by the fitted compatibility statistic D_k; the event-information condition of Section 1 excluded no candidate in the applications. This ranking induced a sequence of nested retained sets, formed by including auxiliaries sequentially from the closest onward. The retained-set size K was then selected jointly with the penalty parameters on the target training split: we increased K only when adding the next auxiliary improved the training-side selection criterion, and otherwise stopped at the current selected value of K. Operationally, this replaces direct tuning of the theoretical thresholds (c_1, t_2) by a data-driven retained-set choice (Proposition 1).

Final refitting. After the retained auxiliary set and penalty values had been chosen, the target-centered Cox model within TransSurv was refit on the full target training split together with the retained auxiliary cohorts. The fitted coefficients from this final refit were then used to compute the held-out target risk scores reported in the main text. No additional tuning was performed after this final refit step.

Optimization details. The target-centered Cox model was fitted by the joint proximal-gradient solver in Section 7.2, with Breslow handling of ties in the Cox partial likelihood. Continuous predictors were preprocessed as described in Methods Section 4.3 before model fitting. The implementation used an ℓ_2 stabilization parameter $\mu = 10^{-6}$, initial step size 1.0, and backtracking line search. Convergence was assessed by the relative change in the full parameter vector $(\beta, \delta_1, \ldots, \delta_K)$, with stopping tolerance 10^{-5} and at most 400 outer iterations. Numerical safeguards included dynamic log-sum-exp stabilization in the Breslow likelihood evaluation, lower bounds on risk-set denominators and relative-norm calculations, and removal of zero-variance columns after split-specific preprocessing.

Comparator implementation. The target-only and pooled penalized Cox comparators used the same preprocessed inputs, and the same held-out target evaluation protocol as TransSurv. The target-only and pooled gene models were then fit with the same proximal-gradient ℓ_1-penalized Cox solver, using $\mu = 10^{-6}$, initial step size 1.0, tolerance 10^{-5}, and at most 600 iterations. MetaSurv was run using the original authors' public implementation, with the hyperparameter strategy described in the original paper and only minimal feasibility adjustments for the size of the target training split. **[E11]** The $\ell_{2,1}$-regularized transfer comparator implements the two-domain shared-support objective of Li et al. [2016]: the per-domain mean Breslow negative log partial likelihoods of a source domain and the target are combined with the row-wise penalty

$\lambda \sum_j \|(\beta_{s,j}, \beta_{t,j})\|_2$ over the shared gene coordinates and a small ridge term ($\mu = 10^{-6}$); target-side clinical covariates are ridge-penalized only, never $\ell_{2,1}$-penalized. Because the method is single-source, the within-disease auxiliary cohorts of each application form its source domain: the stage-stratified KIRC cohorts pooled for JAVELIN Renal 101, the same-cancer alternative stage-defined cohort for each TCGA target, and the TCGA and METABRIC TNBC cohorts pooled for FUSCC. The objective is minimized by the same composite proximal-gradient scheme as the TransSurv solver (Section 7.2) with the row-wise group soft-thresholding proximal step, backtracking line search, Breslow tie handling, convergence tolerance 10^{-5}, and at most 400 iterations, warm-started along a decreasing penalty path. The penalty was parameterized as $\lambda = c\sqrt{\log p/(n_s + n_t)}$ with the multiplier c selected per replication over the grid $\{0.1, 0.3, 1, 3, 10\}$ on the evaluation metrics (the mean of the C-index and the two horizon-specific AUCs), a selection favorable to the comparator; all grid results were retained.

8 TransSurv compatibility report as an inspectable borrowing-disclosure artefact

This section documents the structure of the compatibility report that TransSurv produces by construction for every fitted model, and maps its components to the dataset-representativeness, training–test independence, and user-information principles emphasized in recent clinical-AI deployment guidance [Sendak et al., 2020, U.S. Food and Drug Administration et al., 2021]. The intent is to make explicit that the borrowing operation is inspectable whenever a TransSurv-derived risk score is reported: a downstream user can read, from the report alone, which auxiliary cohorts contributed to the target-centered fit, how strongly each contributed, on which genes the borrowing operated, and which candidate cohorts were screened out.

Components of the compatibility report. A TransSurv compatibility report consists of three components produced from the fitted model:

1. **Auxiliary-cohort retention table.** For each candidate auxiliary cohort $k \in \{1, \ldots, K\}$, the table reports the cohort identifier, sample size n_k, observed event count, event-fraction I_k, fitted contrast size $D_k = \|\widehat{\delta}^{(k)}\|_1$, the screening decision (retained / screened out), and the screening decision reason (contrast criterion, event-information gate, or both). This makes the retain/exclude decision inspectable on a per-cohort basis.

2. **Per-cohort borrowing summary.** For each *retained* auxiliary cohort, the report lists the number of genes on which the auxiliary deviation is non-zero, the sparsity ratio of $\widehat{\delta}^{(k)}$, and the maximum absolute deviation. This quantifies how much cohort-specific correction TransSurv applied, separately for each retained auxiliary cohort.

3. **Coefficient–deviation visualization.** The radial coefficient–deviation plot (main text) and the rectangular reference heatmap (Sections 2.4.2 and 2.4.3) display the estimated target-cohort gene-expression effects $\widehat{\omega}_0$ and the retained auxiliary-specific deviations $\widehat{\delta}^{(k)}$ jointly, so that the dominant target-associated signals and the localized cohort-specific corrections can be inspected together.

Mapping to GMLP guiding principles. The compatibility report corresponds to specific principles in the FDA / Health Canada / MHRA Good Machine Learning Practice guidance [U.S. Food and Drug Administration et al., 2021]. The mapping is summarized in Supplementary Table 12.

Supplementary Table 12: Mapping between TransSurv compatibility-report components and selected FDA/Health Canada/MHRA Good Machine Learning Practice guiding principles [U.S. Food and Drug Administration et al., 2021].

GMLP principle	TransSurv compatibility-report component
Principle 3: Clinical study participants and data sets are representative of the intended patient population.	The retention table makes explicit which candidate auxiliary cohorts entered the target-centered fit and which were screened out, so a user can inspect whether the data feeding the fit are representative of the intended target population.
Principle 4: Training data sets are independent of test sets.	All TransSurv data-adaptive steps (preprocessing, screening, retained-set selection, tuning, fitting) are restricted to the target training split; held-out target patients enter only at evaluation. The retention table makes this separation inspectable.
Principle 7: Focus is placed on the performance of the human–AI team.	The per-cohort borrowing summary and the coefficient–deviation visualization let a clinician or biostatistician inspect the borrowing source and magnitude before clinical interpretation of the risk score.
Principle 9: Users are provided clear, essential information.	The compatibility report can accompany a fitted risk score released to downstream users, providing dataset-level, gene-level, and cohort-level transparency analogous to the "model facts label" framework proposed in Sendak et al. [2020].

References

Domenica Lorusso et al. Pembrolizumab or placebo with chemoradiotherapy followed by pembrolizumab or placebo for high-risk locally advanced cervical cancer (engot-cx11/gog-3047/keynote-a18): overall survival results from a randomised, double-blind, placebo-controlled, phase 3 trial. *The Lancet*, 404(10462):1514–1524, 2024. doi: 10.1016/S0140-6736(24)01808-7. PMID: 39288779.

Matthew McCormack et al. Induction chemotherapy plus chemoradiotherapy versus chemoradiotherapy alone in locally advanced cervical cancer (interlace): an international, multicentre, randomised, phase 3 trial. *The Lancet*, 404(10462):1525–1535, 2024. doi: 10.1016/S0140-6736(24) 01438-7. PMID: 39419054.

Francesco Guerrera et al. Early and long-term survival predictors in stage i lung cancer patients: a decision tree model. *European Journal of Cardio-Thoracic Surgery*, 47(6):1037–1043, 2015. doi: 10.1093/ejcts/ezu410. PMID: 25391390.

Hongchao Pan, Richard Gray, Jeremy Braybrooke, et al. 20-year risks of breast-cancer recurrence after stopping endocrine therapy at 5 years. *New England Journal of Medicine*, 377(19):1836–1846, 2017. doi: 10.1056/NEJMoa1701830. PMID: 29117498.

Joseph A Sparano, Robert J Gray, Della F Makower, et al. Adjuvant chemotherapy guided by a 21-gene expression assay in breast cancer. *New England Journal of Medicine*, 379(2):111–121, 2018. doi: 10.1056/NEJMoa1804710. PMID: 29860917.

National Cancer Institute, Surveillance Research Program. Kidney and renal pelvis cancer — cancer stat facts. `https://seer.cancer.gov/statfacts/html/kidrp.html`, 2025. Accessed 2026-01-25.

Krishnansu S Tewari, Robert A Burger, Danielle Enserro, et al. Final overall survival of a randomized trial of bevacizumab for primary treatment of ovarian cancer. *Journal of Clinical Oncology*, 37(25):2317–2328, 2019. doi: 10.1200/JCO.19.01009. PMID: 31216226.

Rebecca Dent, Maureen Trudeau, Kathleen I Pritchard, et al. Triple-negative breast cancer: clinical features and patterns of recurrence. *Clinical Cancer Research*, 13(15 Pt 1):4429–4434, 2007. doi: 10.1158/1078-0432.CCR-06-3045. PMID: 17671126.

T. Powles et al. Renal cell carcinoma: ESMO clinical practice guideline for diagnosis, treatment and follow-up. *Annals of Oncology*, 35(8):692–706, 2024. doi: 10.1016/j.annonc.2024.05.537.

Deborah K. Armstrong, Ronald D. Alvarez, Floor J. Backes, et al. NCCN guidelines® insights: Ovarian cancer, version 3.2022. *Journal of the National Comprehensive Cancer Network*, 20(9): 972–980, 2022. doi: 10.6004/jnccn.2022.0047.

Nadeem R. Abu-Rustum, Catheryn M. Yashar, Rebecca Arend, et al. NCCN guidelines® insights: Cervical cancer, version 1.2024. *Journal of the National Comprehensive Cancer Network*, 21(12): 1224–1233, 2023. doi: 10.6004/jnccn.2023.0062.

P. E. Postmus, K. M. Kerr, M. Oudkerk, et al. Early and locally advanced non-small-cell lung cancer (NSCLC): ESMO clinical practice guidelines for diagnosis, treatment and follow-up. *Annals of Oncology*, 28(suppl_4):iv1–iv21, 2017. doi: 10.1093/annonc/mdx222.

Per Kragh Andersen and Richard D Gill. Cox's regression model for counting processes: A large sample study. *The Annals of Statistics*, 10(4):1100–1120, 1982. doi: 10.1214/aos/1176345976.

Thomas R Fleming and David P Harrington. *Counting Processes and Survival Analysis*. Wiley, New York, 1991.

Sara A van de Geer. *Empirical Processes in M-Estimation*. Cambridge University Press, Cambridge, 2000.

Jian Huang, Tingni Sun, Zhiliang Ying, Yi Yu, and Cun-Hui Zhang. Oracle inequalities for the lasso in the Cox model. *The Annals of Statistics*, 41(3):1142–1165, 2013. doi: 10.1214/13-AOS1098.

Shengchun Kong and Bin Nan. Non-asymptotic oracle inequalities for the high-dimensional Cox regression via lasso. *Statistica Sinica*, 24(1):25–42, 2014. doi: 10.5705/ss.2012.240.

Jianqing Fan and Jinchi Lv. Sure independence screening for ultrahigh dimensional feature space. *Journal of the Royal Statistical Society: Series B (Statistical Methodology)*, 70(5):849–911, 2008. doi: 10.1111/j.1467-9868.2008.00674.x.

Ethan X Fang, Yang Ning, and Han Liu. Testing and confidence intervals for high dimensional proportional hazards models. *Journal of the Royal Statistical Society: Series B (Statistical Methodology)*, 79(5):1415–1437, 2017. doi: 10.1111/rssb.12224.

Yan Li, Lu Wang, Jie Wang, Jieping Ye, and Chandan K. Reddy. Transfer learning for survival analysis via efficient l2,1-norm regularized cox regression. In *2016 IEEE 16th International Conference on Data Mining (ICDM)*, pages 231–240. IEEE, 2016. doi: 10.1109/ICDM.2016.0034.

Mark P. Sendak, Michael Gao, Nathan Brajer, and Suresh Balu. Presenting machine learning model information to clinical end users with model facts labels. *npj Digital Medicine*, 3:41, 2020. doi: 10.1038/s41746-020-0253-3.

U.S. Food and Drug Administration, Health Canada, and Medicines and Healthcare products Regulatory Agency. Good machine learning practice for medical device development: Guiding principles. https://www.fda.gov/medical-devices/software-medical-device-samd/good-machine-learning-practice-medical-device-development-guiding-principles, 2021.
